# Manananggal - a novel viewer for alternative splicing events

**DOI:** 10.1186/s12859-017-1548-5

**Published:** 2017-02-21

**Authors:** Matthias Barann, Ralf Zimmer, Fabian Birzele

**Affiliations:** 1 Roche Pharma Research & Early Development, Roche Innovation Center Munich, Nonnenwald 2, 82377 Penzberg, Germany; 2 Practical Informatics and Bioinformatics Group, Department of Informatics, Ludwig-Maximilians-Universität München, Amalienstrasse 17, D-80333 Munich, Germany; 3 Roche Pharma Research & Early Development, Roche Innovation Center Basel, Grenzacherstraße 124, 4052 Basel, Switzerland

**Keywords:** Web application, Visualization, Alternative splicing, RNASeq

## Abstract

**Background:**

Alternative splicing is an important cellular mechanism that can be analyzed by RNA sequencing. However, identification of splicing events in an automated fashion is error-prone. Thus, further validation is required to select reliable instances of alternative splicing events (ASEs). There are only few tools specifically designed for interactive inspection of ASEs and available visualization approaches can be significantly improved.

**Results:**

Here, we present Manananggal, an application specifically designed for the identification of splicing events in next generation sequencing data. Manananggal includes a web application for visual inspection and a command line tool that allows for ASE detection. We compare the sashimi plots available in the IGV Viewer, the DEXSeq splicing plots and SpliceSeq to the Manananggal interface and discuss the advantages and drawbacks of these tools. We show that sashimi plots (such as those used by the IGV Viewer and SpliceSeq) offer a practical solution for simple ASEs, but also indicate short-comings for highly complex genes.

**Conclusion:**

Manananggal is an interactive web application that offers functions specifically tailored to the identification of alternative splicing events that other tools are lacking. The ability to select a subset of isoforms allows an easier interpretation of complex alternative splicing events. In contrast to SpliceSeq and the DEXSeq splicing plot, Manananggal does not obscure the gene structure by showing full transcript models that makes it easier to determine which isoforms are expressed and which are not.

**Electronic supplementary material:**

The online version of this article (doi:10.1186/s12859-017-1548-5) contains supplementary material, which is available to authorized users.

## Background

Eukaryotic transcripts share features with a vampire-like creature of Philippine mythology: the Manananggals, nocturnal creatures that prey on pregnant women and feed on the blood and hearts of fetuses. These creatures have the ability to split their torso into two parts, which allows the upper part to fly into the night to go hunting while the vulnerable lower part remains stationary. Whereas transcripts do not share the Manananggal’s lust for blood and hearts, they are able to reshape themselves by losing parts of their substance. This process, called “splicing”, rips out some introns and exons to generate new transcripts that translate into proteins with potential distinct functions. Aside from some exceptions, most transcripts depend on additional proteins (splice factors) for efficient splicing. We call the ability to generate a multitude of isoforms from a single genomic locus *alternative splicing* (AS). It is accomplished by skipping whole exons, using alternative splice acceptor and donor sites or retaining introns.

Splicing allows cells to increase the number of potentially functional RNAs and proteins without increasing the size of the genome. The current GENCODE [1] gene annotation (v23) for the human genome includes more than three times as many transcripts than genes (60,498 genes; 198,619 transcripts). Evidence has been gathered for the involvement of alternative splicing in neurological disorders (e.g. autism [2], Huntington’s disease [3], spinal muscular atrophy [4]), autoimmune diseases (e.g. multiple sclerosis [5], systemic lupus erythematosus [6], Kawasaki disease [7]) and tumorigenesis [8–11]. Understanding this aberrant splicing behavior could translate into a health benefit for patients.

The advent of next-generation sequencing (NGS), and in particular RNA sequencing (RNASeq), simplified the detection and quantification of splicing events in a broad range of genes in a single experiment and a number of tools have been developed for this task.

Some assess alternative splicing by assigning reads to complete isoforms based on statistical models (Cufflinks [12], MMSEQ [13]), while other tools focus on single exon coverage (e.g. DEXSeq [14]) or a combination of junction-spanning reads and exon coverage (e.g. MATS [15]). To circumvent the problem of incomplete annotation, tools like Cufflinks, Trinity [16] and Trans-ABySS [17] perform a genome-guided or *de novo* assembly of transcripts. This also allows these tools to identify completely novel isoforms. Manananggal visualizes novel splicing events (in known genes) as incomplete isoforms that are reduced to the putative exon start and exon end surrounding the novel splice junction(s). However, it cannot detect completely new genes.

All tools usually generate vast lists of potential alternative splicing events between conditions. However, in many cases these events represent false positives, a finding that is supported by previously published tool comparisons that detected little overlap between the result lists of these tools [18]. Hence, it is strongly advisable to validate alternative splicing events by other means. Visual inspection of the data can already strengthen the evidence for an alternative splicing event without the necessity to validate a large number of events in the wet lab.

Unfortunately, the few available interactive tools for visual inspection of alternative splicing in RNASeq data are scarce and in most cases not flexible enough to get a good visualization of the splicing events and the involved isoforms. Therefore, we developed Manananggal, a web application designed to facilitate the visual inspection of alternative splicing events.

## Implementation

Manananggal was implemented in Java using the freely available community edition of the ZK framework. A server is required to deploy the application and a configuration file for the server must be prepared to specify where Manananggal finds reference and project data. Each sample of a project requires two input files: a bigwig file and a junction count file that must be specified in a project file along with some metadata. The user manual that comes with Manananggal explains how these files can be obtained. For internal calculations, Manananggal relies on size factors to adjust for differences in the library size. The size factors can be generated using the command line tool that is included in the Manananggal.jar file (also explained in the user manual). Alternatively, users may add their own size factor estimates (e.g. from DEXSeq [14]) to the project file.

**Fig. 1:**
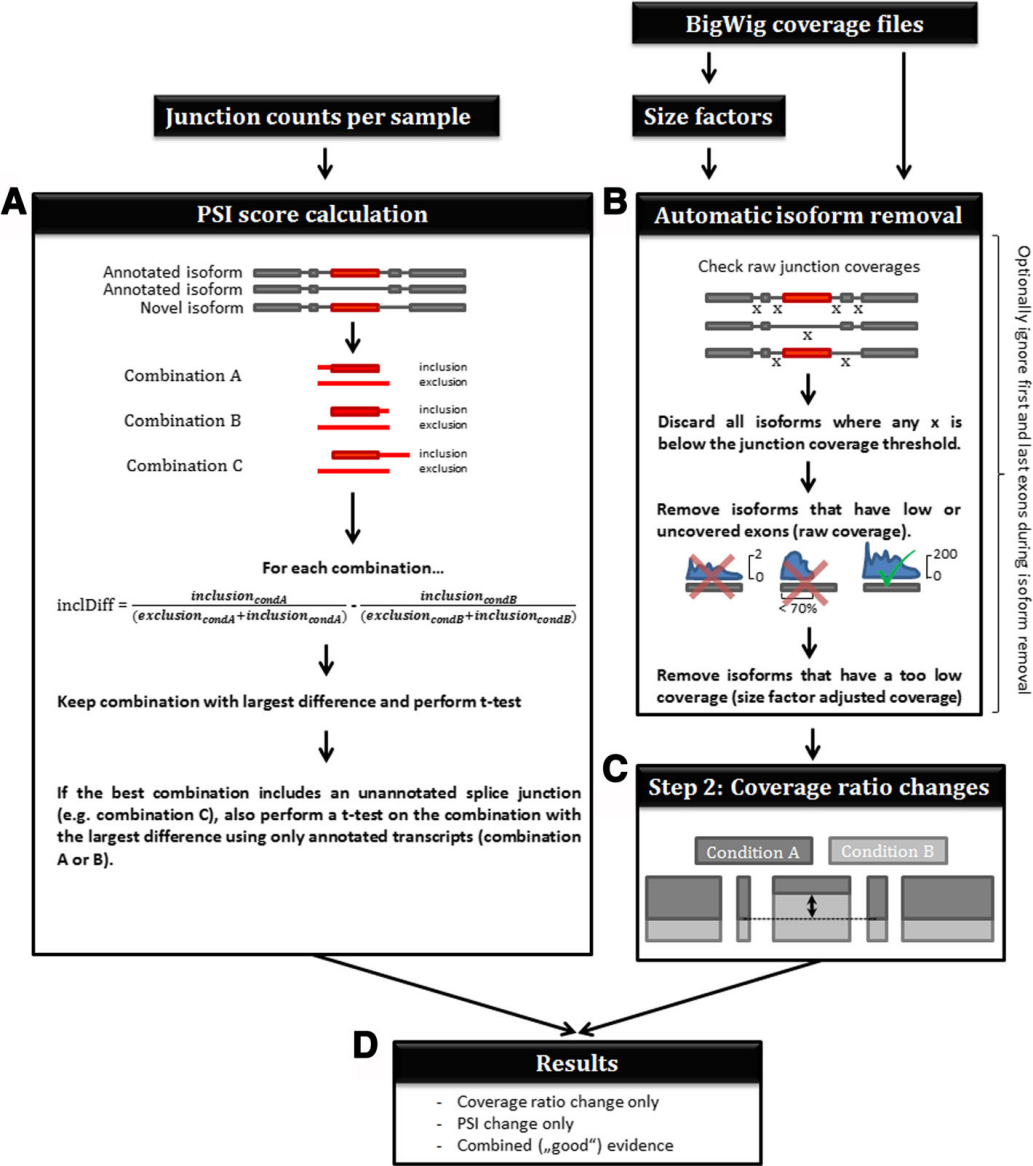
Workflow of the Manananggal method. Junction counts are merged to generate one large table with all possible junctions. The table is indexed for random access and used to identify splice junction pairs that differ between conditions (**a**). One junction in each pair corresponds to a junction arguing for the inclusion and another for the exclusion of the exon. All possible inclusion/exclusion junction combinations for an exon are used to identify the combination that shows the largest difference between conditions. This combination is subsequently used in a *t*-test to provide a *p*-value for sorting of the results. If the best junction pair includes read counts of an unannotated junction we also report the best available result using only known junctions. The right side of the image illustrates the detection of coverage ratio changes. BigWig files are used to calculate size factors for each sample that are used to adjust for sequencing depth differences. **b** An automated isoform selection discards isoforms that contain exons with insufficient coverage from the analysis. **c** The arrow indicates an alternatively spliced exon that shows a large change in the coverage ratio (depicted by dark grey and light grey color for each condition) compared to the remaining exons (or exon groups). Changes in the coverage ratios between different conditions are identified by testing the coverage ratios of each nucleotide of the query exon against the mean coverage ratios of the remaining exons/exon groups. **d** Finally, a result list is generated that contains ASE candidates that are supported by PSI scores only, ratio changes only, or both

When a data set is opened and a gene is selected in the web application, the Manananggal method tries to identify all alternative splicing events in the gene. Candidate events are added to a result list in the top right corner of the web interface. A short overview on how this algorithm works is shown in Fig. 1.

**Fig. 2:**
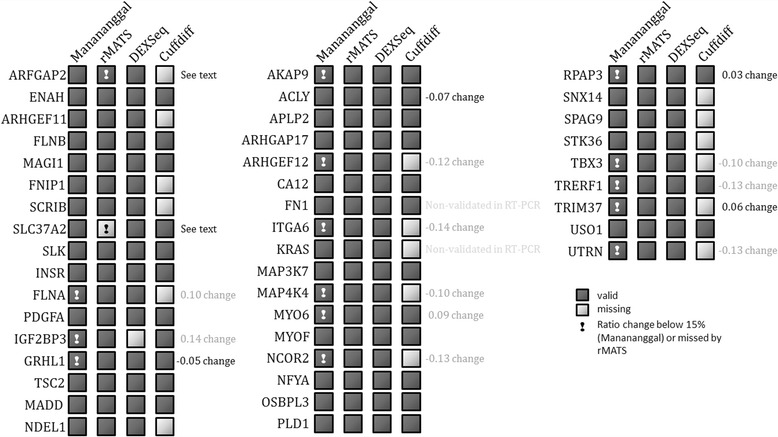
Comparison of detected AS candidates to 41 RT-PCR confirmed AS events. rMATS was originally used by Lu et al. (2014) [24] and Shen et al. (2014) [19] for the detection of the AS events in the PC-3E and GS689.Li prostate cell lines. They chose 43 AS events and validated 41 by RT-PCR. For two genes (FN1 and KRAS) the RT-PCR failed and, thus, it is unclear whether they represent real events. All events were identified by Manananggal. However, several events showed only very small changes (<15%, numbers to the right of rows with exclamation mark). rMATS detected all events but the exon skipping in SLC37A2 and reported a high FDR for the event in ARFGAP2. DEXSeq detected all events but the one in IGF2BP3 and Cuffdiff failed to detect 17 of these exon skipping events

In brief, the algorithm works as follows: junction count files are used to calculate PSI scores for each pair of conditions specified in the project file and bigwig files are used to identify changes in the coverage ratio of exons. We used a greedy implementation of the PSI score that uses only a single junction for measuring the inclusion count of an exon. The reason behind this is, that terminal exons (e.g. start or end exons) of a transcript are only supported by a single junction and exon skipping events might refer to exons that are connected to more than one exon, resulting in an imbalanced count value for neighboring junctions that could yield wrong results. We compared our algorithm to other tools such as rMATS, Cuffdiff and DEXSeq. In our evaluation it performs comparable to rMATS and DEXSeq and outperformed Cuffdiff with respect to the detection of alternative splicing candidates (Fig. 2, see Additional file 1: Supplementary Material chapters III to IV for a detailed comparison of the methods). However, Manananggal is significantly faster than rMATS and Cuffdiff (Additional file 1: Table S4) and can thus be used on the fly. Please refer to the user manual if you would like to run the Manananggal stand-alone console application to identify alternative splicing events in your project.

**Fig. 3:**
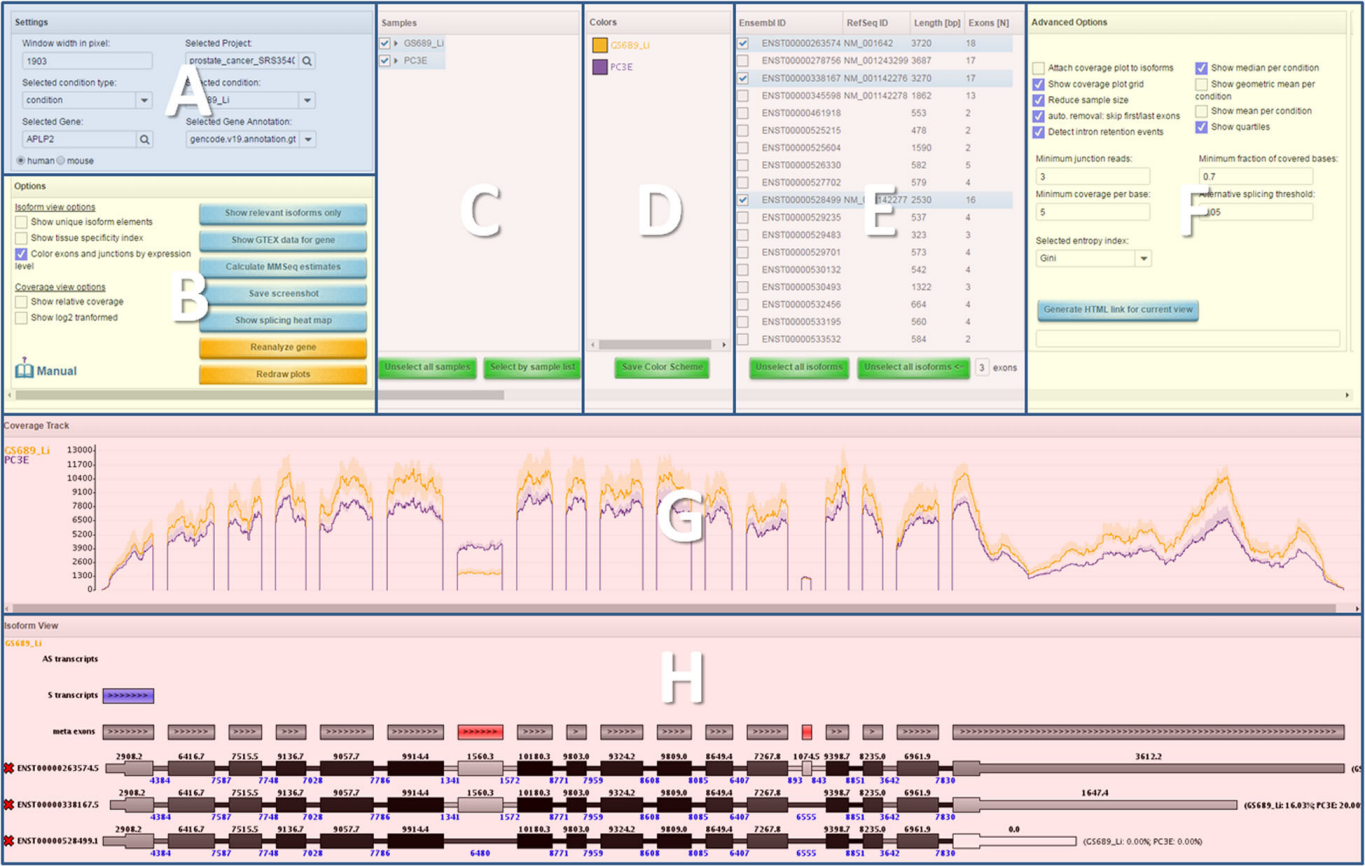
Overview of the Manananggal user interface. **a** Project specific selections, e.g. what condition type should be used for grouping samples. **b** options that affect the visual output shown in **g** and **h**, also includes a link to the user manual. **c** Specific samples can be selected here. **d** Colors for each condition can be defined here. **e** Isoforms may be selected here. **f** Additional options that affect the visual output and detection of alternatively spliced exons. Also includes a button to create a HTML link to the current view. **g** Coverage plots that by default show one coverage line per condition. **h** The isoform plot indicates overlapping transcripts in sense and antisense direction, highlights potential exon skipping events and shows each of the selected isoforms. Please note that the image is not showing the complete Manananggal interface. A multi-column table of alternative splicing candidates is located in the top right corner of the web interface that we omitted for reasons of clarity

The Manananggal user interface, shown in Fig. 3, offers a wide range of options. Usually, users do not have to worry about most of them and can just use the default settings. However, genes with a very large number of exons might for example require that users define a larger window width to plot them correctly. A larger window width can also be used to zoom into the gene. The interface also offers ways to select or unselect certain samples (e.g. outliers) and isoforms (e.g. if they are unexpressed). For each sample group users may select their own color that is a helpful feature for people with color deficiency or when certain colors are generally associated with a certain phenotype. Further, an automatically generated list of predicted alternative splicing events, based on the algorithm described above, provides a comfortable way to focus on these events. Exon skipping events that show differences in the exon coverage ratios and PSI score are also indicated on the meta exon track in the isoform view, where meta exons are chromosomal regions defined by the minimum start and maximum end position of all overlapping exons. Other types of ASEs are not highlighted because they tend to include more false positives if unexpressed isoforms are selected (see Additional file 1: Figure S8 for a more detailed explanation).

Another feature provided by Manananggal is the ability to share your results with others. In the advanced options window is a button generates HTML links for the current selections, which includes the selected data set, gene reference, samples and isoforms. Adding the keyword “&screenshot” to the URL facilitates sharing of results when many samples or very large genes are involved. The viewer will generate a screenshot the first time the link is accessed and load this screenshot for every subsequent use of the URL. Further, users can rate and save interesting alternative splicing events to a list that is automatically loaded whenever someone opens the same project. This list is located in the top-right corner of the web interface.

**Fig. 4:**
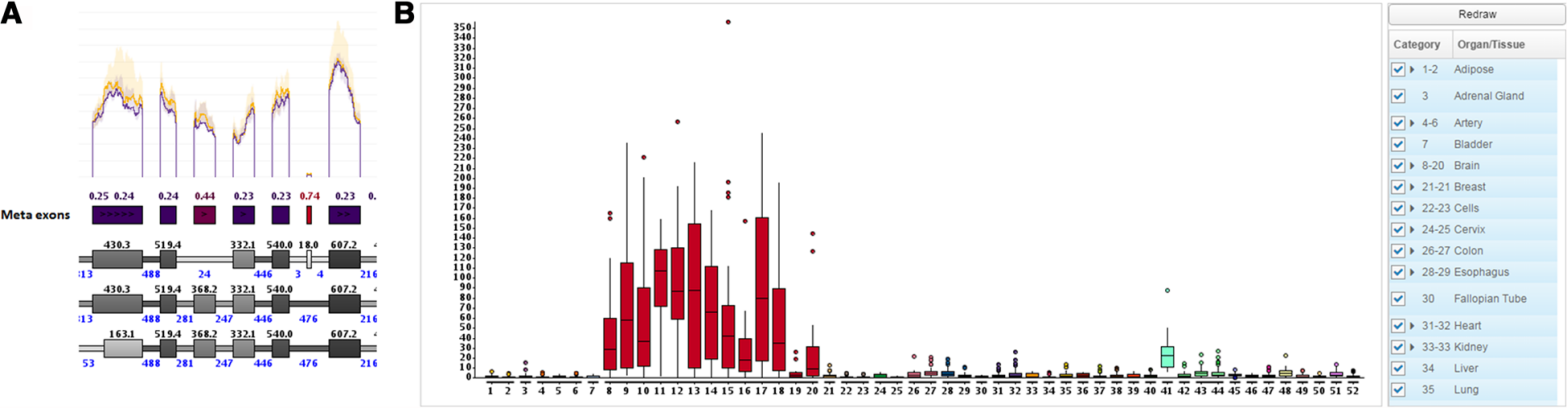
Tissue specific expression. **a** shows the tissue specificity for exonic parts on the meta exon track. One of the meta exons is colored in red with a specificity of 0.74. The specificity ranges from 0 (unspecific) to 1 (very specific). **b** shows the popup window that opens after clicking the red colored exonic part. It becomes apparent that the exon is almost exclusively expressed in different brain tissues (*red boxes*)

Sometimes it might be important to know in which tissues a gene or exon is expressed, e.g. when searching for very specific ASEs. To visualize this information we provide multiple options, but all of them require that users have access to tissue specific gene and exon expression data (e.g. GTEX). Option one opens a boxplot that shows the expression of the whole gene in all tissues. Option two uses the meta exon track to highlight tissue specific exonic parts (Fig. 4a) that can be clicked to open a popup window that shows a boxplot for the exons expression in all tissues (Fig. 4b).

A more detailed explanation of all the functions is given in the user manual that can be accessed by clicking on the “Manual” button located in section B of Fig. 3.

## Results

In the following, we will show how Manananggal can be used to inspect ASEs and discuss its advantages over other tools. If possible, we tried to use the same data set and gene for the comparison. We used the prostate cancer data set published with the rMATS publication [19] (Accession number: SRS354082). The data includes three samples for each of two prostate cancer cell lines (GS689_Li and PC3E).

**Fig. 5:**
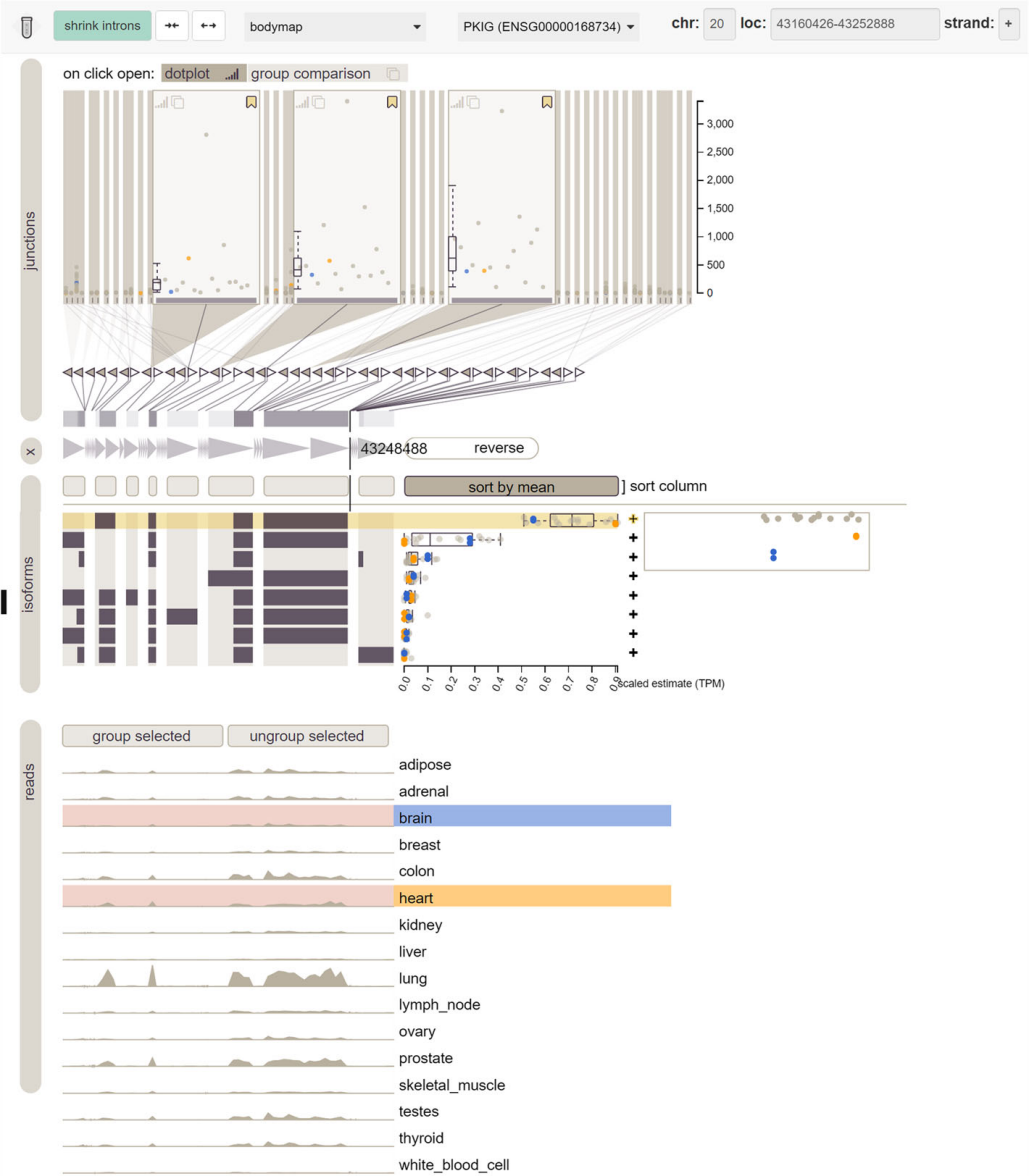
Vials Visualization of an alternative splicing event in PKIG that uses different promoters in hear and brain. Junction frequencies are shown at the top, isoforms and MISO isoform expression estimates in the middle and coverage tracks at the bottom

One tool that was developed to visualize alternative splicing is Vials [20]. We used the publically available online installation of Vials (http://www.vials.io/vials) to compare its features to Manananggal’s. Since the tool includes bodymap data, we decided to go for an alternative splicing event in PKIG between heart and brain that we will also use for the comparison of another tool later. According to the GTEX portal and also the tissue specific data stored in SpliceSeq, different isoforms of PKIG are expressed in brain and heart that use two different promoters. Figure 5 shows PKIG in the Vials web interface. The top view shows the frequency of all junctions in all samples. For demonstration purposes, we selected the first isoform, which shows all junctions of the isoform in wider columns. A larger difference between brain (blue) and heart (orange) can be observed for the first junction, which appears to be more frequently expressed in heart than brain. This data is supported by the isoform track below that shows isoform expression estimates to the right. As shown, the first isoform is more often expressed in heart and the second isoform is more often expressed in brain. The difference is not so obvious in the coverage tracks at the bottom, because the coverage for all samples is shown relative to the maximum coverage of all tissues, similar to Manananggal. However, Manananggal has an option to unselect groups or single samples dynamically, while Vials relies on different source files that define the groups. Therefore, users can unselect high coverage tissues that are not of interest in Manananggal and get a clearer picture of the coverage tracks, which requires additional effort in Vials. The dot and boxplots of the junction coverage track are helpful, but also a bit tedious because you have to compare each isoform to each other and then decide where the differences are. Instead, one will usually rely on the isoform expression estimates by MISO to detect alternatively spliced isoforms and then check the junctions for these. While this works very well in this example, isoform expression estimates are often very wrong for complex genes or when using Gencode, which includes also incomplete isoforms. Imagine a gene with multiple alternative splicing events that don’t allow for unambiguous isoform expression quantification. In this scenario MISO estimates are less informative and it is necessary to identify the alternative splicing change manually by examining all junctions one by one, which is very time consuming for large genes. Manananggal on the other hand is focused on single splicing events and provides a list of potential splicing events each time a new gene is opened. This facilitates the identification of splicing events even for complex genes if you don’t have prior knowledge of the events of interest. If desired, isoform expression estimates can also be shown behind each isoform in the isoform view of Manananggal that are generated using MMSeq. Compared to Vials, Manananggal also offers additional features that Vials lacks, such as: dynamic coloring of sample groups, direct comparison of isoform specific junction counts for identified alternative splicing events, interactive sample selection, merged coverage plots, ability to freely choose isoforms, saving and sharing the current view via HTML links, log2 transformation of the coverage, and some other features.

**Fig. 6:**
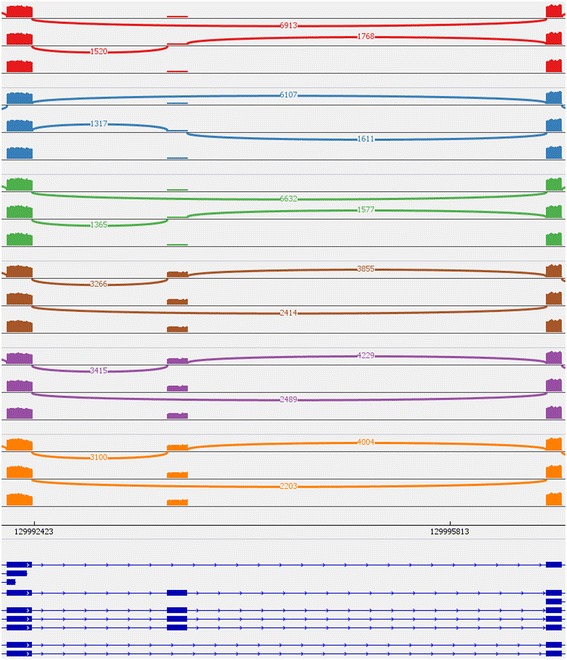
Sashimi plot taken from the IGV Viewer that shows an ASE in the gene APLP2. Each different color represents a different sample (the last track is the gene annotation track). The first three samples represent GS689_Li samples and the last three samples belong to the prostate cancer cell line PC3E. The plot shows that GS689_Li samples express the middle exon less frequent than PC3E samples. Only junctions with a minimum read coverage of 100 are shown

One very popular tool for visualization of Next-Generation-Sequencing data is the IGV Viewer. It is a platform independent application that can visualize a broad range of data types. For the inspection of ASEs it includes an option to visualize the data as so called sashimi plot [21]. Figure 6 shows an example of such a plot for an ASE in *APLP2*. The first three samples (each sample has a different color) refer to the GS689_Li samples and the last three to the PC3E samples. For genes with few isoforms sashimi plots are easy to interpret. In the example, it is clear that the middle exon is lower expressed in the GS689_Li samples than in the PC3E samples, and the count number of the exclusion junctions supports this as well. However, imagine you have four different conditions with 10 samples each. This would result in an enormous plot that would be much more difficult to interpret. The inability of the IGV viewer to group samples into a single plot is a big disadvantage for larger projects. Further, introns are shown to scale, resulting in very small exons. The list of isoforms at the bottom is also fixed and removing unexpressed isoforms is only possible by editing the gene annotation file.

**Fig. 7:**
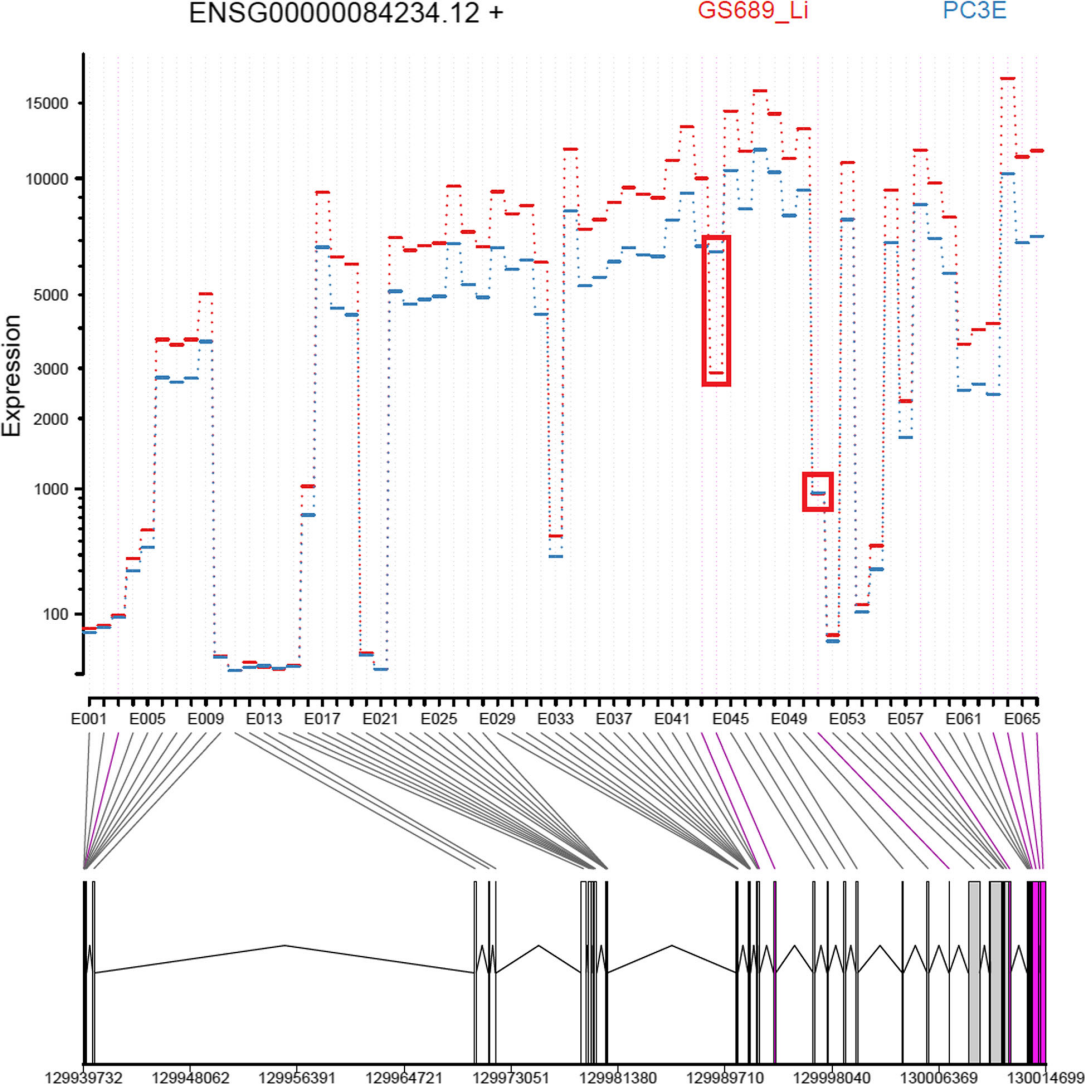
DEXSeq’s plot for the visualization of alternative splicing events. Shown at the top is the coverage for both sample groups separately. Each line corresponds to a single exonic part. The outlined exonic parts are two strong ASEs. The bottom part of the image shows the gene structure and depicts differentially expressed exonic parts by adding color to the exonic part and the line connecting the exonic part to the x-axis of the coverage plot. Two alternative splicing events were framed with red rectangles that represent the major ASEs in this gene

DEXSeq comes with a plot function that could be combined with web frameworks (such as Shiny) to create a somewhat interactive web interface that produces splicing images for single genes on demand. Figure 7 shows such a plot using the same data set and gene as before. For easier interpretation we marked two alternative splicing events that are present in *APLP2* by red rectangles. The first event corresponds to the event shown for the IGV Viewer. The top of the plot shows the coverage of exonic parts and the lower part shows a flattened gene model. The gene track at the bottom indicates differentially spliced exonic parts by adding color to them. Especially the terminal exons are indicated as differentially expressed. The advantage of this plot over the IGV sashimi plot is that it combines the coverage of all samples within a group and, thus, it can be effectively used to visually inspect a large number of samples. Another plus is that the plot shows all exonic parts at once, thus, multiple events may be investigated at the same time. The largest disadvantage is the use of exonic parts. This obscures the true gene structure and makes it very hard to tell which exonic part belongs to which exon. Further, the DEXSeq plot does not provide information on overlapping transcripts that could be the reason of false positive ASEs.

**Fig. 8:**
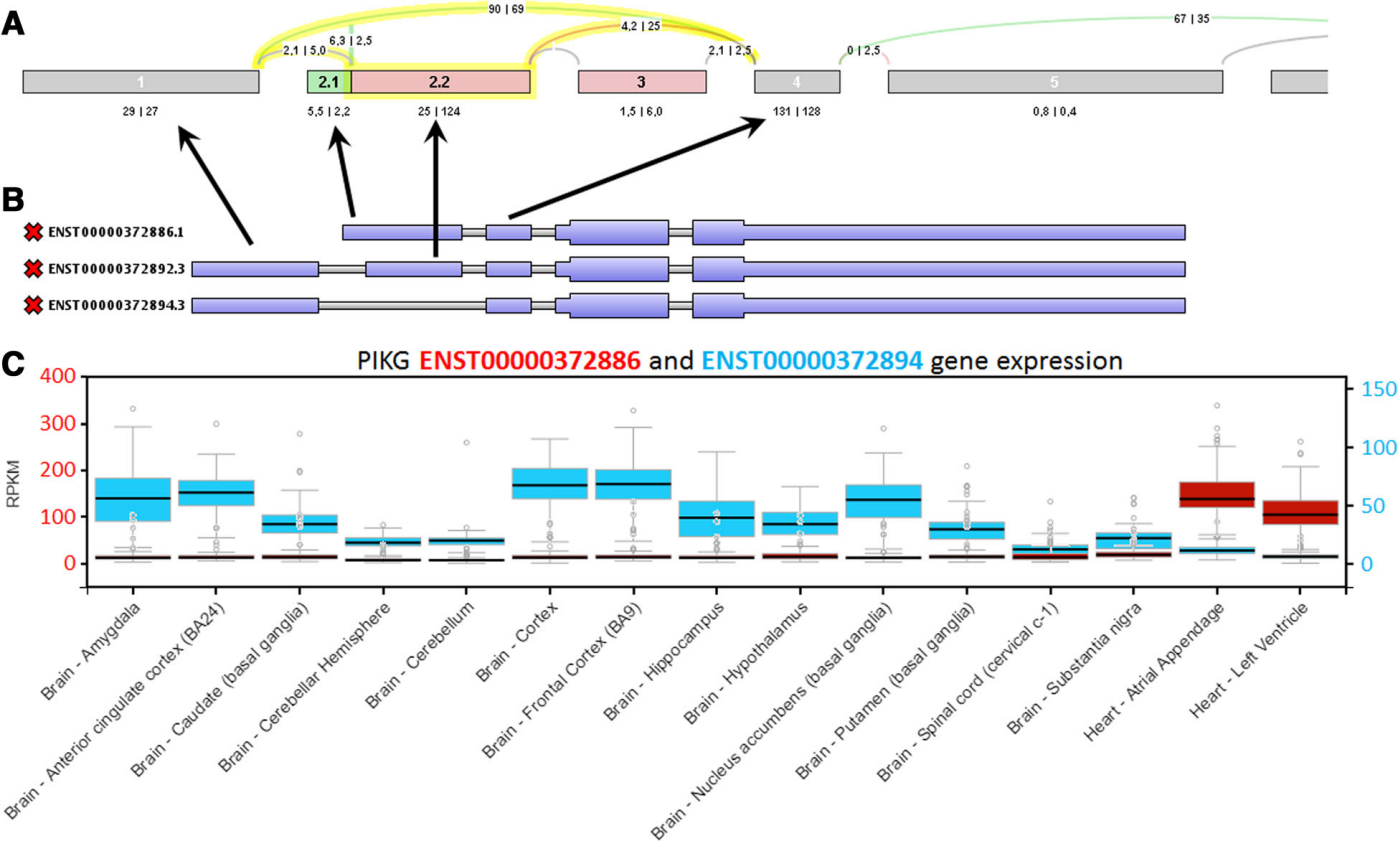
Visual representation of an alternative splicing event in SpliceSeq. **a** shows the visual representation inside the SpliceSeq viewer. All exons of the gene are shown in a single line. Overlapping exons are merged into meta exons that indicate which part of the meta exon belong to which exon. Expression numbers are given for each exonic part and junction. The ASE is highlighted in yellow by SpliceSeq to make users aware of the elements involved in the splicing event. **b** shows some of the structure of three *PKIG* transcripts that were copied from Manananggal to emphasize that exon 2.1 represents an alternative transcript start. **c** shows a combined image of the expression of the first and last transcript shown in **b** in multiple brain and heart tissues that was obtained from the GTEX portal (http://www.gtexportal.org/). It demonstrates that ENST00000372886 is expressed in heart and ENST00000372894 in brain

Next, we tried to produce images for the same gene using SpliceSeq [22]. Compared to the other tools SpliceSeq cannot use previously mapped data and, thus, requires fastq files that are then mapped using bowtie. On a windows computer this process failed for the whole data set. Using a reduced sequence file (only reads mapping to the *CD44* gene locus) we were able to successfully map the data and import it into the SpliceSeq database. Unfortunately, the program fails at the isoform generation step for an unknown reason. Without the source code we could not dig deeper into the problem and, therefore, decided to discuss an example using the data set that is provided with the tool. Figure 8a shows an alternative splicing event in *PKIG* using data from brain and heart.

The graphical representation is very similar to the IGV sashimi plots with three important differences. First, there is only a single graph showing the read counts for each group that allows the comparison of a large number of samples. Second, introns are drawn with a fixed length allowing for the investigation of a much larger part of the gene at once, and third, alternative splicing events are highlighted. Disadvantageous are the lack of coverage plots and a missing indication of overlapping transcripts that make it difficult to spot problems that arise from differential expression or antisense transcription. Further, the example also shows how this representation can be very misleading. The highlighted event has been classified as an ES (exon skipping) event by SpliceSeq and the visual representation also suggests that this is an exon skipping event. However, considering the read numbers it becomes clear that the major event might not be exon skipping. Another disadvantage of SpliceSeq is that it is not possible to hide exons that belong to isoforms that are either absent or very lowly expressed (e.g. exon 3), thus giving the sashimi plot a more complicated look than would be necessary.

**Fig. 9:**
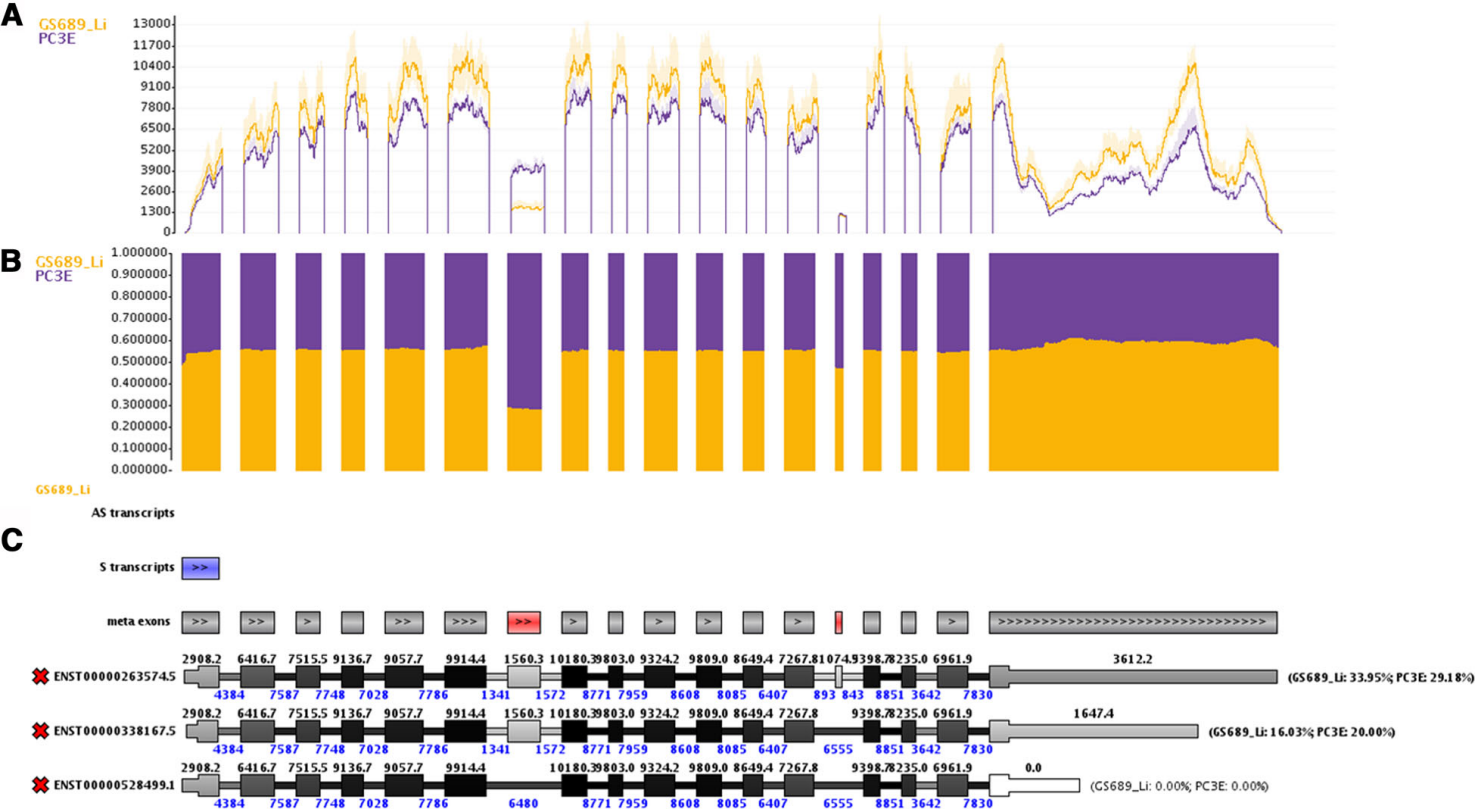
Visual output for *APLP2* from Manananggal. **a** shows the standard coverage plot for both sample groups. The light shaded regions around each line represent the upper and lower quartile of the coverage. **b** shows the coverage ratio representation of the coverage plot. Using ratios flattens the coverage for all exons and makes it easier to spot the alternatively spliced exons that appear at ‘bumps’ in the coverage ratio that otherwise remains very constant across all exons. **c** shows the isoforms that are likely involved in the ASEs. The antisense transcript track is empty and the sense transcript track only shows one exon of APLP2 that overlaps an intronic region of another transcript on the same strand (indicated by the light blue box). Therefore, it is unlikely that overlapping transcripts influence the coverage of *APLP2*, especially at the ASEs. Further, the automatic ASE detection algorithm of Manananggal identified both ASEs and indicates them by coloring the respective meta exons in red. The percent numbers at the end of each transcript correspond to MMSEQ isoform expression estimates that can be generated if Manananggal has access to MMSEQ. However, this also shows that MMSEQ assigned a large fraction of the coverage to other transcripts that are probably less important or not expressed at all

We implemented several improvements over the other tools in Manananggal. Similar to the DEXSeq plot and SpliceSeq we combine the data of multiple samples into a single plot. DEXSeq also showed the per group coverage for each exonic part but only provides a single coverage value for each exonic part and does not indicate the range of the expression. In contrast, Manananggal also shows the upper and lower quartile of the coverage at each base position and users can choose between mean or median representation. Coverage differences between conditions may be large and, thus, we also provide options to show the log2 coverage and coverage ratios. Coverage ratio plots proofed to be very helpful for spotting ASEs. Figure 9 shows the *APLP2* splicing event from the prostate cancer in the Manananggal viewer.

A standard coverage plot is shown at the top, the alternative coverage ratio plot in the middle and the isoforms at the bottom. Colors for each condition may be freely changed to consider the needs of people with color deficiency. The isoform plot indicates overlapping antisense (AS) and sense (S) transcripts. AS transcripts, which are absent in this case, are shown in orange (for non-exonic overlap) and red (exonic overlap). Overlapping transcripts in sense direction are shown in light blue (non-exonic) and dark blue (exonic). While overlap to exonic regions of other genes is a frequent origin of false positive alternative splicing results, overlap with non-exonic regions usually does not result in false positive alternative splicing events, but we believe it is helpful to make the user aware of a potential overlapping transcript. The meta exon track indicates potentially alternatively spliced exons (highlighted in red) and indicates the orientation of the gene. In the picture shown, uninformative isoforms (i.e. isoforms that are not providing additional expressed junction paths) were unselected. If available to the server, Manananggal can run MMSEQ to produce isoform quantifications that will be displayed at the end of each isoform for each condition. For large sample sets this can take a long time and might even exhaust the resources of the server, thus, it should be carefully considered whether to enable MMSEQ or not (e.g. working with projects that have only some dozens of samples should be fine). In contrast to the DEXSeq plot and SpliceSeq, Manananggal preserves the full transcript structure, thus, making it easier to tell which exon is actually involved in an ASE.

**Fig. 10:**
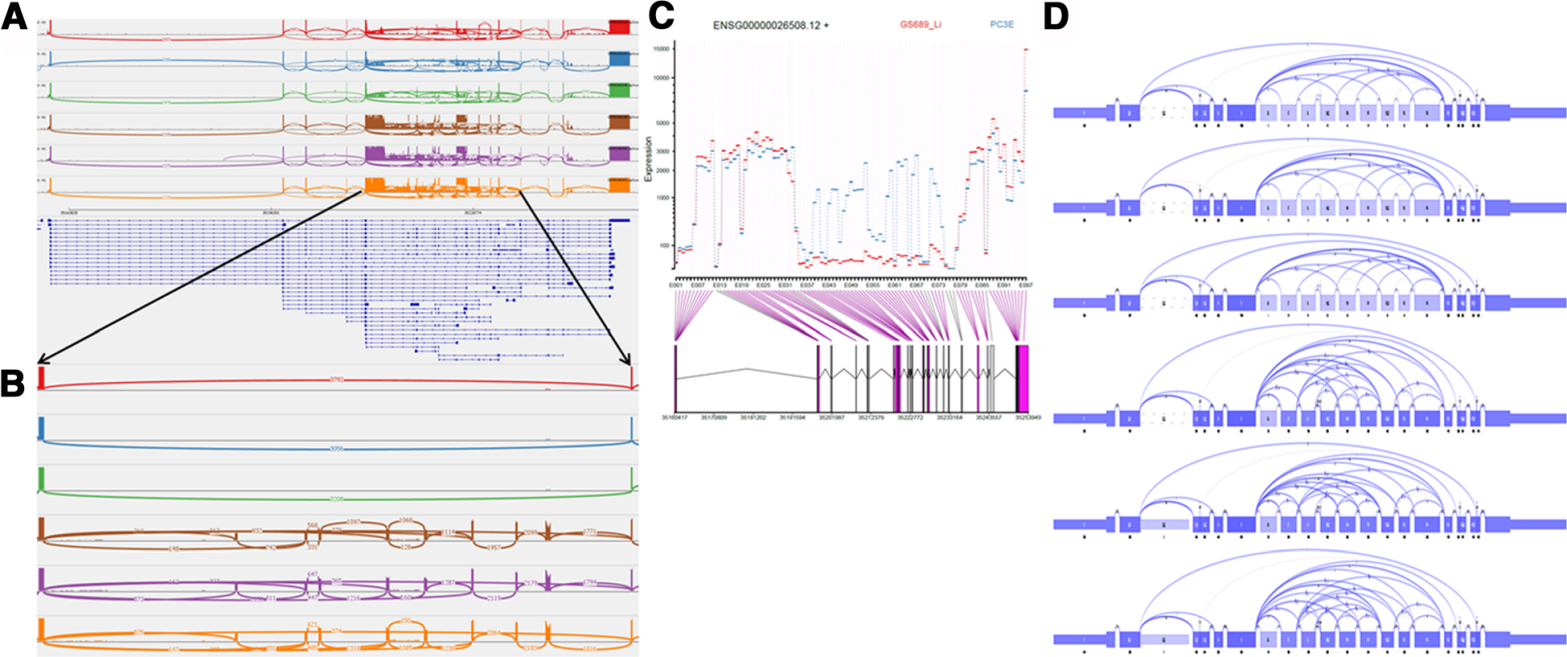
Visual representation of CD44 in the IGV Viewer, DEXSeq and SpliceSeq. **a** shows a sashimi plot of the *CD44* gene with all junctions visible. A difference between the first three (GS689_Li) and last three samples (PC3E) is visible, but it is very challenging to identify which junctions belong to the ASEs or what ASEs are actually present in the data set. **b** shows a sashimi plot that was zoomed to the region of interest. Junctions with a coverage below 100 were hidden from the picture to get a cleaner view of the region. It becomes clear that the GS689_Li samples mostly skip all optional exons in this region, while *CD44* expresses multiple of them. However, it remains difficult to tell which isoforms are expressed in *CD44*. **c** shows the DEXSeq plot for *CD44*. A large number of exonic parts appear to be differentially expressed. **d** shows the sashimi plots for all 6 samples in SpliceSeq. A coverage difference is visible for all optional exons that are lower expressed in the GS689_Li samples. The PC3E samples show a large number of junctions

Although Manananggal provides many abilities that were tailored to inspect alternative splicing event, all of the above mentioned tools might be sufficient to visually inspect simple splicing events, such as the one shown in *APLP2*. However, the real strength of Manananggal is the ability to investigate highly complex genes. Figure 10a shows the CD44 locus using the IGV Viewer and the prostate cancer data set (see Additional file 2: Figure S1 for higher resolution images).

By looking at the image it becomes clear that there is some difference between the GS689_Li samples and PC3E samples, but it is not possible to decipher what that difference is. The IGV viewer offers to hide junctions based on a count threshold. Figure 10b (see Additional file 3: Figure S2.png for higher resolution images) shows the *CD44* region after exon 5, a region with multiple optional exons, using a junction count threshold of 100. Now it’s much more obvious that the GS689_Li samples mostly express an isoform that skips all of the optional exons, while for the PC3E samples it remains less clear which isoforms are expressed.

Similar, the DEXSeq plot (Fig. 10c, see Additional file 4: Figure S3 for higher resolution images) shows nicely that the GS689_Li samples skip all the optional exons, but it does not help to identify the isoforms that may be expressed in PC3E. Figure 10d (see Additional file 5: Figure S4 for higher resolution images) shows the SpliceSeq representation for *CD44* for each sample separately (as mentioned before, we were unable to run the isoform generation step and, thus, cannot show the per group estimates). However, as SpliceSeq does not offer an option to hide lower expressed junctions, we expect that the combined graph for all samples would look equally complicated. However, similar to the other tools, also this example indicates a difference between the two sample groups, but it just does not provide enough information to identify the most important isoforms.

**Fig. 11:**
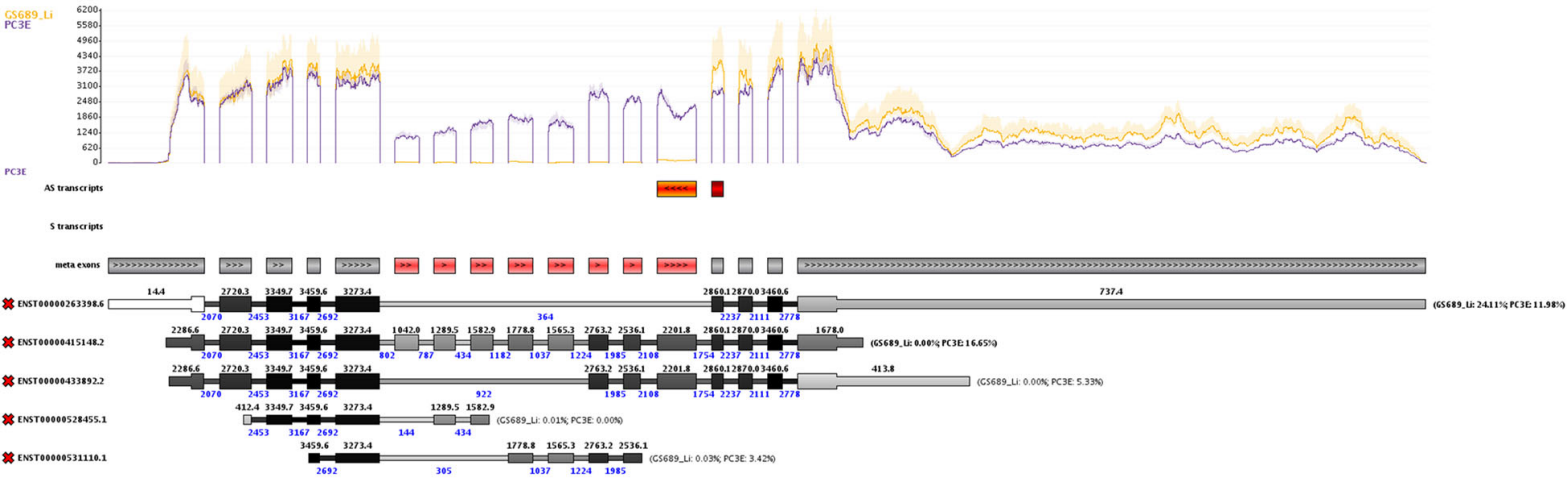
Manananggal’s visual representation of alternative splicing events in CD44. The coverage plot at the top shows that multiple exons are only expressed in the PC3E samples. These alternatively spliced exons are also highlighted on the meta exon track. One exon appears to have a higher expression in GS689_Li samples. However, this exon overlaps an exon of an antisense transcript (indicated by the red box on the AS transcripts track) and thus does not represent a valid alternative splicing event. The isoforms shown at the bottom were selected in a way that represents the coverage plot best. Please note that some of these isoforms (especially the last two) might represent incomplete transcripts that are included in the gene reference

With Manananggal we generated the image shown in Fig. 11. By removal of probably unexpressed isoforms (= no read evidence) we limited the number of isoforms that are obviously expressed in the data set. Compared to the other tools this image appears much cleaner without losing information. On the contrary, additional information becomes visible. The optional exons have different expression heights, thus, multiple isoforms must be expressed in the PC3E data set, and one exon appears to have a larger coverage in the GS689_Li group. However, an exon of an antisense transcript (indicated by a red box) is overlapping this exon and, thus, this coverage difference is very likely not related to an alternative splicing event. Further, the isoforms depicted show the most important splicing events that are necessary to explain the coverage pattern. However, one should bear in mind that not all isoforms included in GENCODE represent full transcripts, thus, some of the shorter isoforms shown here probably lack exons.

## Discussion

Existing viewers like the Integrative Genomics Viewer (IGV) [23] provide ways, e.g. Sashimi plots, to investigate alternative splicing, but the representation becomes very difficult to interpret once multiple samples are investigated or transcript models are complicated. SpliceSeq [22] employs a visualization similar to IGV and, additionally, provides functionality to compare sample groups. Other programs, like DEXSeq provide single exon expression charts. While this also works well for multiple samples, it does not provide the user with information regarding the junction coverage or known transcript models.

## Conclusions

We developed Manananggal, a novel tool for the visualization of alternative splicing events that comes with its own method for AS detection. Compared to the other tools with similar functions, Manananggal provides additional features that facilitate this process (Table 1). Additional features tailored to specific problems are available in Manananggal (not discussed here). These features are thoroughly explained in the user manual that also includes a tutorial section. With Manananggal we provide the community with a freely available web application that can be used by non-experts and experts alike to get more information on their data.

**Table 1:** Comparison of Manananggal to other visualization methods for alternative splicing events

	Manananggal	IGV	SpliceSeq	DEXSeq
Input	BIGWIG + COUNT_FILE	BAM	fastq	BAM
Organism	Human/Mouse^a^	All	All?	All
Interactive	✓	✓	✓	(✗)
Unexpressed isoforms can be hidden	✓	✗	✗	(✗)
Coverage plots are available to spot differential expression	✓	✓	✗	✓
Indication of overlapping antisense transcripts	✓	✓	✗	(✗)
gene/transcript structure	full	full	reduced	reduced
introns	compressed	to scale	compressed	to scale
Indicates AS exons	✓	✗	✓	✓
Allows group comparisons	✓	✗	✓	✓

^a^In principle Manananggal should also work with other organisms. Some functions, such as the cross-reference table are not available for other organisms and thus users would need to use gene IDs rather than symbols to query genes

The DEXSeq plot is an R function that could be modified to add some of the missing features

## Availability and requirements

**Project name:** Manananggal

**Project home page:** https://github.com/barannm/Manananggal

**Operating system(s):** Web application running in any recent browser

**Programming language:** Java

**Other requirements:** Installation requires a Tomcat Web Server where the application can be hosted

**License:** GNU AGPLv3

## Additional files

Additional file 1: Details about the algorithm behind Manananggal and detailed comparisons of the method to other tools. (DOC 4064 kb)
Additional file 2: Figure S1. Additional figure showing the example of CD44 alternative splicing visualized in IGV. (PNG 430 kb)
Additional file 3: Figure S2. Additional figure showing the example of CD44 alternative splicing visualized in IGV (zoom in). (PNG 125 kb)
Additional file 4: Figure S3. Additional figure showing the example of CD44 alternative splicing visualized using SpliceSeq. (PNG 212 kb)
Additional file 5: Figure S4. Additional figure showing the example of CD44 alternative splicing visualized in DEXSeq. (PNG 303 kb)

## Abbreviations

AS: Alternative splicing
ASE: Alternative splicing event
NGS: Next-Generation-Sequencing
RNASeq: RNASequencing
