# Supplementary material for: Manananggal - a novel viewer for alternative splicing events
Source: BMC Bioinformatics. 2017 Feb 21;18:120. doi: 10.1186/s12859-017-1548-5 (PMC5319012; doi:10.1186/s12859-017-1548-5)
Supplement: Additional file 1: — Details about the algorithm behind Manananggal and detailed comparisons of the method to other tools. (DOC 4064 kb) [file 12859_2017_1548_MOESM1_ESM.doc]

***Manananggal - A Novel Viewer For Alternative Splicing Events – Supplementary Material***

**Supplementary chapter I – general statistics and run parameters**

**1.1 Installation and Compilation**

Manananggal is implemented in Java 1.7 and requires multiple 3rd party software packages to work. These include the commons-math3 and commons-lang3 (v3.4) libraries and the Samtools API from PICARD (sam-1.55.jar or higher). For the graphical user interface we used the community edition of the ZK framework library (<http://www.zkoss.org/>). Manananggal is using the IGV API to access bigwig files (downloaded on May 22nd 2015 from <https://github.com/igvteam/igv/>), which we included in the JAR and WAR file for simplicity.

Supplementary Table S1 Junction count data format. Columns marked with an asterix (*) are currently not used and dummy values may be inserted. The file needs to be in tab separated format.

| **Column** | **Information type** | **Example value** |
| --- | --- | --- |
| 1* | Junction type | Novel_Junction |
| 2 | Gene ID | ENSG00000227232.4 |
| 3 | Strand | true or false |
| 4* | Position string | 17364-17498 |
| 5 | Reference name | chr1 |
| 6 | Junction start position | 17364 |
| 7 | Junction end position | 17498 |
| 8 | read count | 15 |

**1.2 Visualization options by different tools**


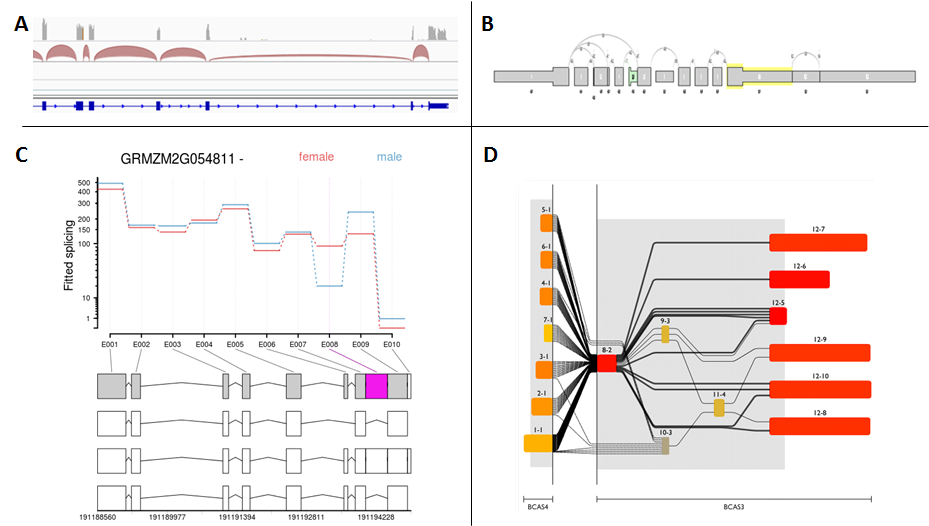


Supplementary Figure S1 - Visual representations of alternative splicing events provided by A the IGV Viewer, B SpliceSeq, C DEXSeq/R and D Genomatics Transcriptome Viewer.


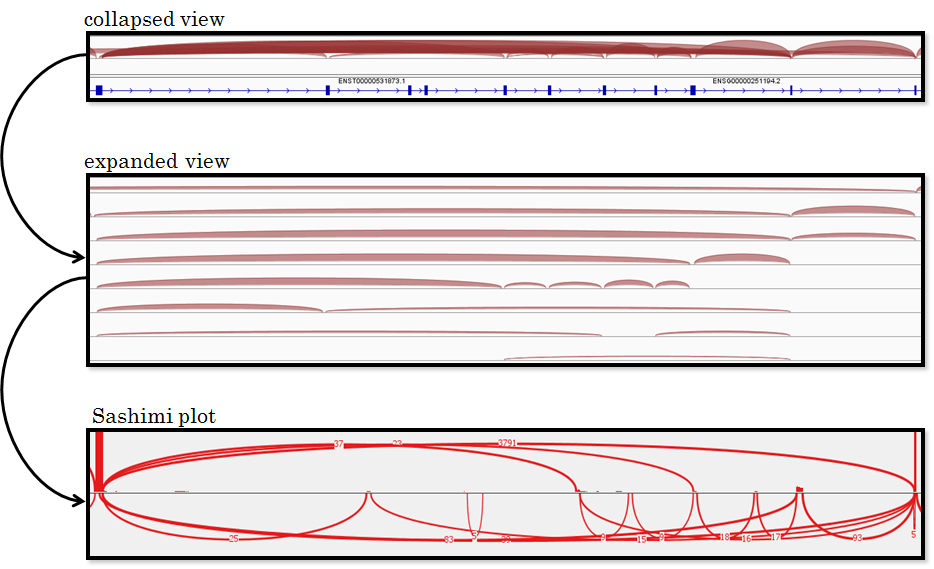


expanded view

Supplementary Figure S2 - IGV representation of junction spanning reads for a gene with a large number of possibly alternatively spliced exons shown in condensed view (top), expanded view (middle) and Sashimi plot (bottom). The collapsed and expanded views are difficult to use for visual inspection of alternative splicing events. The Sashimi plot provides a better representation, but it is still challenging to find differences between multiple samples.

**1.3 Alternative splicing class support**


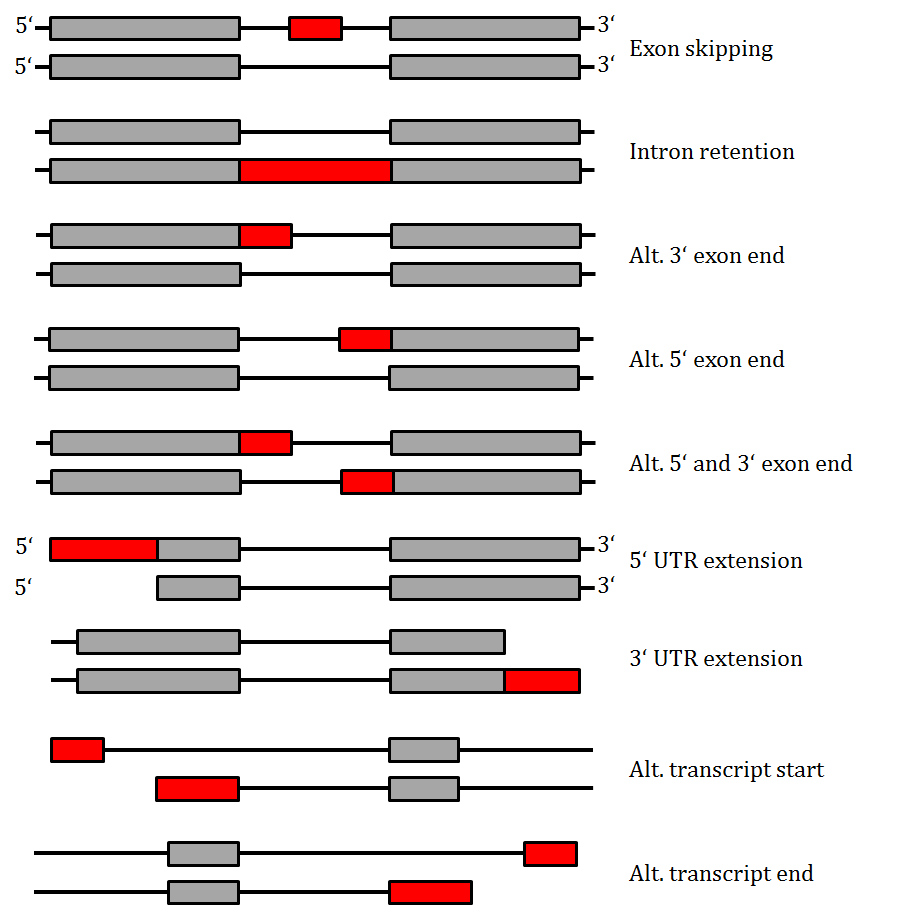


Supplementary Figure S3 – Types of alternative splicing. In general, alternative splicing events can be divided into four major types: exon skipping, modification of exons (e.g. exon extensions), alternative terminal exons and intron retention events. Please note that exon extensions can also affect terminal exons. In this case no junction spanning reads supporting the event exist and the commonly used PSI score calculation cannot be used to identify it.

Supplementary Table S2: Alternative splicing classes supported by the investigated tools.

|  | Exon skipping | Alternative terminal exons | Alternative splice acceptor/donor sites | Retained introns | UTR extensions |
| --- | --- | --- | --- | --- | --- |
| Cuffdiff | **🗸** | **🗸** | **🗸** | **🗸** | ? |
| DEXSeq | **🗸** | **🗸** | **🗸^1^** | **x**[^2^](#_ENREF_1) | **🗸** |
| Manananggal | **🗸** | **🗸** | **🗸**^3^ | **🗸** | **x** |
| rMATS | **🗸** | **x**^4^ | **🗸** | **🗸** | **x** |

^1^ Will depend on the size of the exon extension (tandem repeats will likely be missed)

^2^ The Gene annotation file can be manipulated to include introns as exons, which would allow DEXSeq to process introns as well.

^3^ Subtle (e.g. very short) changes in alternative 3’ and 5’ ends can only be reliably detected by split reads. Therefore Manananggal does not use coverage ratios to identify this type of events.

^4^ The MATS homepage claims that MATS can “automatically detect and analyze alternative splicing events corresponding to all major types of alternative splicing patterns”, however, we could not find any results regarding alternative first or last exons.

1.4 Parameters used and output statistics for all tools

**1.4.0 Mapping**

# single line command

gsnap

--gunzip

-A sam

-s Homo_sapiens.GRCh37.75.gsnap.splicesites.iit

--nthreads=12

--batch=5

-D *REFERENCE_DIR*

-d Human_Genome_HG19

*READ_ONE*

*READ_TWO*

> *ALIGNER_OUTPUT*

GSNAP version 2014-10-22

1.4.1 rMATS

# single line command

python /MATS/rMATS.3.0.9/RNASeq-MATS.py

-b1 ${file_SRR536342},${file_SRR536344),${file_SRR536346}

-b2 ${file_SRR536348},${file_SRR536350},${file_SRR536352}

-gtf gencode.v19.annotation.gtf

-o $output_dir

-t paired

-len 101

-analysis U

**1.4.2 DEXSeq**

#sample description (prostate_cancer_DEXSeq.ini)

Sample condition

SRR536342 GS689_Li

SRR536344 GS689_Li

SRR536346 GS689_Li

SRR536348 PC3E

SRR536350 PC3E

SRR536352 PC3E

#cross reference file

gene_id gene_type gene_symbol gene_desc tm_domain MIM

ENSG00000000003 protein_coding TSPAN6 tetraspanin 6 [Source:HGNC Symbol;Acc:HGNC:11858] Tmhmm

#Prepare annotation

python dexseq_prepare_annotation.py

–r

gencode.v19.annotation.gtf

gencode.v19.no_aggregate.gtf

#Counting

samtools view $file | python dexseq_count_1.6.py

-p yes

-s no

$gencode.v19.no_aggregate.gtf

-

$output_file

# all of the above are single line commands

#DEXSeq

library(DEXSeq)

print(Sys.time())

setwd("/prostate_cancer_cell_lines/DEXSeq/output")

input_dir <- ***[input_dir]***

countFiles = list.files(input_dir, pattern="*.txt$", full.names=TRUE)

flattenedFile = ***[no_aggregate.gtf]***

sample_desc <- read.table(prostate_cancer_DEXSeq.ini", header=T, row.names=1)

design <- ~ sample + exon + condition:exon

## create DEXSeq count data set

print("loading data")

dxd <- DEXSeqDataSetFromHTSeq(countFiles, sample_desc, design, flattenedfile=flattenedFile)

## estimate size factors

print("estimating size factors")

dxd = estimateSizeFactors(dxd)

## prepare worker threads

BPPARAM = SnowParam(workers=8)

print("estimating dispersion")

dxd = estimateDispersions(dxd, BPPARAM=BPPARAM)

print("performing test")

dxd = testForDEU(dxd, BPPARAM=BPPARAM)

## estimate fold changes

dxd = estimateExonFoldChanges(dxd, BPPARAM=BPPARAM)

save(dxd, file="DEXSeq_results.rData")

## get results

dxr = DEXSeqResults(dxd)

## get significant results

mtch <- dxr$pvalue <= 0.05

mtch[is.na(mtch)] <- FALSE

dxr <- dxr[mtch,]

## sort results

dxr <- dxr[order(dxr$padj),]

dxr <- dxr[,-13]

## load xref

fileXref <- “gencode_v19_x_ref_unique_genes.txt"

xref <- read.table(fileXref, header=T, sep="\t")

## add gene symbols

dxr$gene_id=(gsub("\\..*", "", dxr$groupID))

mtch <- match(dxr$gene_id, xref$gene_id)

dxr$gene_symbol <- xref$gene_symbol[mtch]

## write table

write.table(dxr, "DEXSeq_out.txt", sep="\t", row.names=F, quote=F)

print(Sys.time())

We first used the prepare_annotation.py script that comes with DEXSeq without additional parameters. However, the subsequent DEXSeq analysis included mostly false positive results due to aggregated genes. Therefore we repeated this step and used the optional parameter to disable gene aggregation for DEXSeq. Despite a subsequent error (“glmnb.fit() - Too much damping - convergence tolerance not achievable”), we proceeded with the results because this results only in a slight loss of power and the alternative (gene aggregation) is much worse. As we show later, we did not find a large number of false negatives for DEXSeq.

**1.4.3 Tuxedo**

#cufflinks

/cufflinks-2.2.1.Linux_x86_64/cufflinks

-o /cufflinks_out/$file_out

-g gencode.v19.annotation.gtf

-u

-q

-p 8

$file_in

#cuffmerge

/cufflinks-2.2.1.Linux_x86_64/cuffmerge

-g gencode.v19.annotation.gtf

-o $output_dir

-s hg19.fa

-p 8

cuffmerge_list.txt (includes all sample.transcripts.gtf files)

#cuffdiff

/cufflinks-2.2.1.Linux_x86_64/cuffdiff

-o $output_dir

**--no-effective-length-correction**

-p 12

${merged.gtf}

SRR536342.bam,SRR536344.bam,SRR536346.bam

SRR536348.bam,SRR536350.bam,SRR536352.bam

# all of the above are single line commands

We ran Cuffdiff once with and once without effective length correction to reduce inflation of short transcript expression values. We obtained 456 significant results with correction and 476 without correction. The shorter list was, besides some few exceptions, completely contained in the longer list. Therefore we proceeded with the data generated without effective length correction.

1.4.4. Manananggal

#project file

sample condition size_factors bigwig_files junction_count_files

SRR536342 GS689_Li 1.079 SRR536342.bw SRR536342.junction_cnts.tsv

…

#merging of count files

java -cp /lib/*:Manananggal.jar Manananggal.SplicingAnalyzer merge prostate_cancer_SRS354082.project

# calculate size factors

java -cp lib/*:Manananggal.jar Manananggal.SplicingAnalyzer calculate_size_factors gencode.v19.annotation.gtf prostate_cancer_SRS354082.project

#data analysis example for the parameter set 3_5_0.7_0.05_true

java -cp /lib/*:Manananggal.jar Manananggal.SplicingAnalyzer

gencode.v19.annotation.gtf

prostate_cancer_SRS354082.project

condition

3

5

0.7

0.05

biomart_cross_ref.txt

-skipFirstAndLast

-threads=8

# all of the above are single line commands

Supplementary Table S3 - List of parameter sets used for Manananggal. Two different thresholds were chosen for the minimum number of junction spanning reads (3 and 5) and minimum coverage per base (5 and 10) each. The minimum coverage fraction per exon was set to 70% and coverage ratio thresholds ranged from 0.05 to 0.15. Additionally, we ran each combination with and without removal of first and last exons.

| **Setting** | **min. jun.**  **read coverage** | **min. coverage** | **min.**  **covered bases** | **cov. ratio threshold** | **Removal of first and last exons** |
| --- | --- | --- | --- | --- | --- |
| **A** | 3 | 5 | 0.7 | 0.05 | false |
| **B** | 5 | 5 | 0.7 | 0.05 | false |
| **C** | 5 | 5 | 0.7 | 0.10 | false |
| **D** | 5 | 5 | 0.7 | 0.15 | false |
| **E** | 5 | 10 | 0.7 | 0.05 | false |
| **F** | 3 | 5 | 0.7 | 0.05 | true |
| **G** | 5 | 5 | 0.7 | 0.05 | true |
| **H** | 5 | 5 | 0.7 | 0.10 | true |
| **I** | 5 | 5 | 0.7 | 0.15 | true |
| **J** | 5 | 10 | 0.7 | 0.05 | true |

Supplementary Table S4 – Run time of the used tools. Manananggal and DEXSeq were the fastest tools by a large margin. For the 6 samples investigated in this study it took only about 1.5 to 2 hours to identify alternative splicing events. Even when including another 4-6 hours for the generation of count files and BigWig files they are much faster than the next fastest tool: rMATS. rMATS required already more than two days, but also produces a more comprehensive output than Manananggal that includes alternative splicing events other than skipped exons. The tuxedo pipeline (Cufflinks/Cuffmerge/Cuffdiff) was the slowest of all and required roughly a week of processing time to finish. Results from larger experiments (63 samples) show increasing sample numbers do not lead to a vast increase of processing time of Manananggal.

| **Tool** | **Runtime**  **dd:hh:mm:ss** | **Settings** | **Notes** |
| --- | --- | --- | --- |
| **Manananggal** | 00:02:23:53*  to  00:03:13:26* | Multiple | for more details see |
| **Manananggal** | 00:11:12:54* | 5_5_0.7_0.1_true | Different experiment, comprising 63 samples. |
| **DEXSeq** | 00:00:39:40* | Without gene aggregation |  |
| **rMATS** | 02:18:17:00 | Default | Run time can be decreased by running the pipeline manually and then process all variant calling steps as separate threads. This would have reduced the run-time by about an hour. |
| **Cufflinks**  **(8 samples)** | 06:09:04:00  05:10:42:00  03:11:53:00  05:10:05:00  04:08:19:00  05:08:55:00 | Default | 8 threads per sample. |
| **Cuffmerge** | 00:05:31:00 | Default | 8 threads |
| **Cuffdiff** | 00:13:59:00 | Default | 8 threads |
| **Cuffxxxx (total)** | 07:04:34:00 |  |  |

*This processing time does not include the time required to produce the input files (count files and in case of Manananggal also BigWig files), which takes typically a few hours per sample. As this can be done for all samples in parallel it does not increase processing time substantially for larger data sets.

**1.1 Number of identified AS**

Depending on the parametrization, Manananggal reported between 1,045 and 6,705 exon skipping (SE) candidates and between 1,274 and 9,007 alternative terminal exons (ATEs). Exon extensions and retained introns were detected using only one source of information (split reads or coverage ratio) and are thus more prone to errors. The results included between 38,028 and 89,195 exon extension events and 1,569 to 6,939 retained introns. Filtering for EEs that were flagged as ‘significant’ or EEs that use only known junctions dramatically reduce the number of EEs. As expected, the choice of threshold for the coverage ratio change has the largest impact on the number of reported events. Therefore, a coverage ratio change threshold of 0.05 (5% difference) reports a significantly larger number of potential AS candidates than a threshold of 0.15. The filter for junction coverage has moderate effects (and gets rid of events supported by only few reads) while changing requirements for exon coverage had little effect on the overall results. Skipping first and last exons during the isoform removal step increases the number of identified AS events by 20 to 25%. It seems reasonable to use moderately strict settings for the minimum junction and exon coverage and low coverage ratio thresholds (the user can still filter the results for the strongest changes afterwards).

DEXSeq reported a very large number of potential AS events (63,158 candidates, p-value <=0.05,). More than half of these ASEs had an adjusted p-value <= 0.1 (34,110 events). Compared to DEXSeq, rMATS reported many fewer potential AS events (8,557 candidates, p-value <= 0.05). Less than half of them had a FDR <= 0.05 (3,079 candidates). Cuffdiff had the lowest number of potential AS events (1,665 candidates, p-value <= 0.05) of which about 25% (467) were tagged as “significant” results. Further, we noticed Cuffdiff sometimes reported the same locus multiple times, reducing the number of unique results.

Supplementary Table S5 - Run time and number of results for each parameter set used for Manananggal. The number of detected exon skipping events (SE) that are supported by coverage and PSI score ranges from 1,045 to 6,705 in the different combinations. As expected, the *coverage ratio change* threshold has the largest impact, e.g. fewer results show larger changes and more results show small changes. The second largest impact on the number of SE events had the *minimum number of junction spanning read*s threshold, resulting in 5,166 and 5,986 AS events using a threshold of 3 reads and 3,755 and 4,513 using a threshold of 5 reads. Increasing the required *coverage per base value* from 5 to 10 had little impact on the number of SE events, reducing the number of events from 3,755 to 3,475 and from 4,513 to 4,248. The optional parameter to ignore first and last exons when deciding which isoforms should be dropt before analysis increases the number of SE events detected by both measurements by 20-25%. The numbers for the other AS types behave very similar. However, their numbers are much higher for several reasons. The number of alternative terminal exons (ATEs) is only slightly higher, which might reflect that one differentially expressed alternative start or end exon can be combined with multiple other start or end exons , thus leading to several results that are actually pointing to the same event. The very high number of exon extension events can be explained by the fact that these events are (currently) only detected via junction spanning reads. A higher *minimum number of junction spanning read*s threshold will help to reduce the number of false positives in this category. Similar, retained introns are (currently) only based on coverage ratio changes. Lacking a second source of information these two categories include more results, including more false positives.

| **parameter set** | **runtime** | **#SEs (“combined”)** | **#ATEs (“combined”)** | **#EEs** | **#RIs** |
| --- | --- | --- | --- | --- | --- |
| **3**_5_0.7_0.05_false | 02:49:10 | 5,166 | 4,828 | 83,194 | 5,271 |
| **3**_5_0.7_0.05_true | 02:27:28 | 5,986 | 9,007 | 89,195 | 5,560 |
| 5_5_0.7_**0.01**_false | 03:09:02 | 6,705 | 7,703 | 41,362 | 6,939 |
| 5_5_0.7_**0.05**_false | 02:23:53 | 3,755 | 4,446 | 38,418 | 5,247 |
| 5_5_0.7_**0.05**_true | 02:29:17 | 4,513 | 8,284 | 38,436 | 5,552 |
| 5_5_0.7_**0.10**_false | 03:11:34 | 1,891 | 2,335 | 38,456 | 2,983 |
| 5_5_0.7_**0.10**_true | 02:26:49 | 2,443 | 4,579 | 41,500 | 3,232 |
| 5_5_0.7_**0.15**_false | 03:11:47 | 1,045 | 1,274 | 38,472 | 1,569 |
| 5_5_0.7_**0.15**_true | 03:00:48 | 1,378 | 2,641 | 41,520 | 1,755 |
| 5_**10**_0.7_0.05_false | 03:10:25 | 3,475 | 3,479 | 38,028 | 4,234 |
| 5_**10**_0.7_0.05_true | 03:13:26 | 4,248 | 8,094 | 41,362 | 4,489 |

Supplementary Table S6 - Number of alternative splicing events detected by Manananggal, DEXSeq, rMATS and Cuffdiff.

| **Tool** | **Detected AS events** | **Detected AS events**  **(conservative)** |
| --- | --- | --- |
| **DEXSeq** | 63,158^1^ | 34,110^2^ |
| **rMATS (SE)** | 8,557^1^ | 3,079^3^ |
| **rMATS (A3SS)** | 588^1^ | 215^3^ |
| **rMATS (A5SS)** | 452^1^ | 148^3^ |
| **rMATS (MXE)** | 2730^1^ | 961^3^ |
| **rMATS (RI)** | 1195^1^ | 879^3^ |
| **rMATS (total)** | 13,522^1^ | 5283^3^ |
| **Cuffdiff** | 1,665^1^ | 476^4^ |

^1^ p-value < 0.05, ^2^ adjusted p-value < 0.1, ^3^ FDR < 0.05; ^4^ p-value <0.05 and significant flag

Supplementary chapter II – The algorithm

**2.1 Preprocessing of junction count files and calculation of size factors**

Junction counts are first merged into a large table for all samples and then indexed to allow random access of the data. The junction counts are used for display for junction read count visualization in the viewer and to calculate scores for different splice-paths (splice-paths will be explained in more detail later). BigWig files are used to calculate size factors in a similar way to DESeq[^1^](#_ENREF_1), and exon coverages. To calculate size factors we first determine the exon groups of a gene. An exon group contains a list of all overlapping exons. Next we calculate the mean expression value for all positions covered by exons for the given gene using the BigWig files. The rest of the calculation works like the estimateSizeFactor function of DESeq: Imagine the data is organized in a large table where the columns correspond to the samples and each row represents one gene. Each sample has one expression value per gene. We then divide each value in a column by the geometric mean of each gene (row), skipping genes with a geometric mean of 0. The size factor for a given sample is then calculated as the median of all quotients per column.

**2.2 Calculation of percent-spliced-in (PSI) scores**

Information on splice-junction usage is processed to calculate “percent-spliced-in” scores. Different to the commonly used formula for PSI score calculation[_ENREF_20](#_ENREF_20)^2^, we use either the read count for the upstream or downstream inclusion splice-junction. The reason for this are highly complex splicing events that may have multiple upstream splice-junctions but only one downstream splice-junction (or *vice versa*). You can think of an example where there is a splicing cassette with two exons that are alternatively spliced (Supplementary Figure S4A). Either both exons are discarded or only the second. This leads to two different paths connecting to the second cassette exon, but only one possible path downstream of it. Hence the downstream junction has a count equally to the sum of the two upstream exons. This leads to a large bias in PSI calculation if one of the two upstream junctions has a low read count and the other is highly covered.

Therefore, we are only considering either an upstream or downstream junction for the PSI score calculation. To be more precise, we search for the inclusion/exclusion junction combination that shows the largest difference between two conditions (Supplementary Figure S4B-D) and calculate a p-value using a two-sided heteroscedastic t-test assuming that different sample conditions or tissues might have different variances depending on the affinity of the splicing factors involved in a particular splicing event in the given tissue/condition to be on the conservative side.


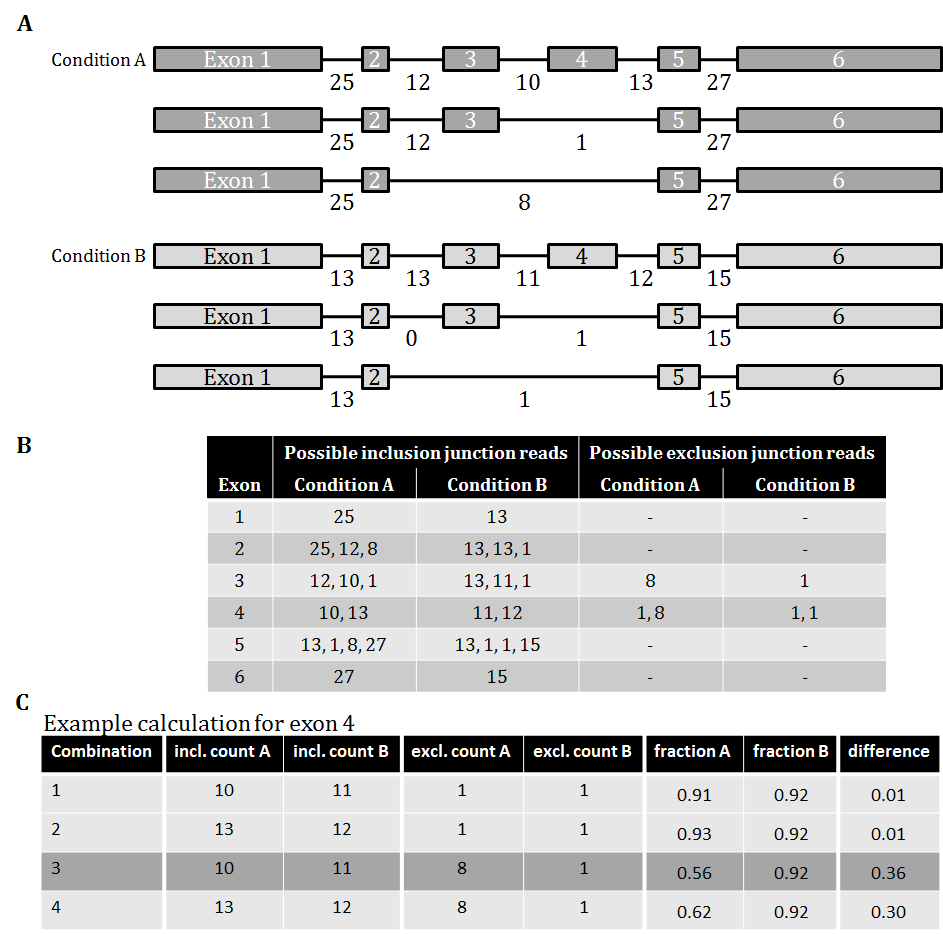


Supplementary Figure S4 - Example PSI score calculation. A A schematic representation of a gene with 3 different isoforms for two different conditions. The numbers below the introns correspond to the junction read count sum adjusted for library size for the respective condition. B For each exon a list of split reads supporting the inclusion and exclusion can be generated. C The combination of inclusion/exclusion junction with largest difference between the conditions is selected from this list and a two-sided heteroscedastic t-test is used to calculate a p-value.

**2.3 Automated isoform selection**

To improve results based on coverage ratio differences, Manananggal discards isoforms that are unlikely to be expressed. This process is divided into three stages. First, isoforms with insufficient splice junction coverage are excluded based on the median coverage per condition (Supplementary Figure S5). Second, isoforms with insufficient exon coverage are discarded. This is based on raw coverage values obtained from the BigWig files. We consider the coverage per base and the fraction of covered bases per exon in this stage. Third, adjusted exon coverages are calculated (see explanation below; Supplementary Figure S6) and isoforms including exons with insufficient coverage per base are discarded.


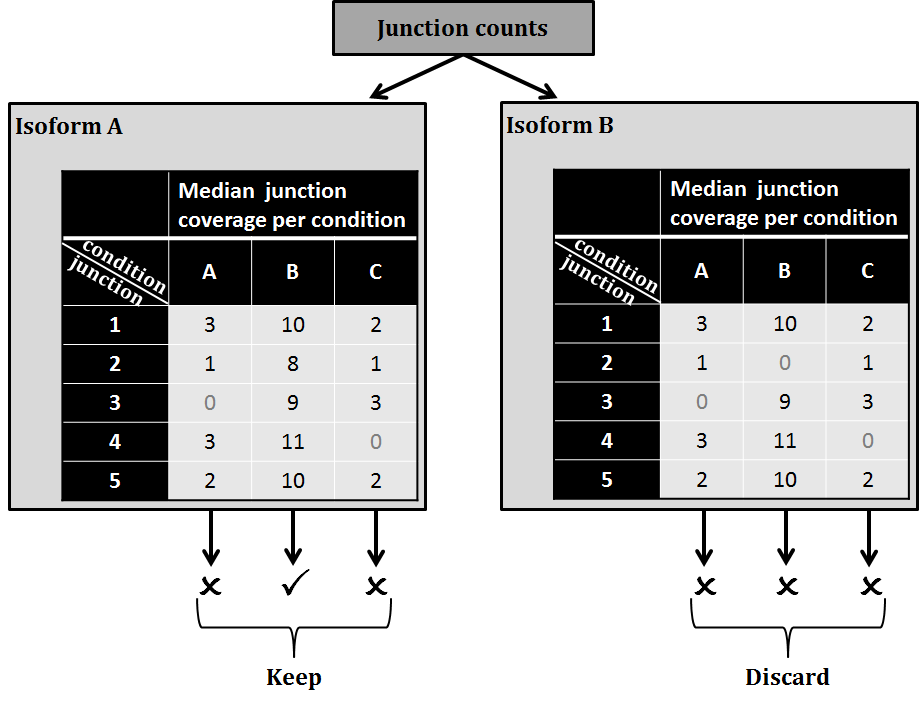


Supplementary Figure S5 – First stage of the automatic isoform filtering. The first stage discards all isoforms that include junctions where the median for all conditions is below a user specified threshold (e.g. 3 reads). Isoforms that were not discarded are subject to the second filtering stage that removes isoforms based on raw exon coverages. First, exon groups are recalculated to adjust for any discarded isoforms and raw coverage values (not modified by size factors) for the new exon groups are obtained from BigWig files. First and last exons of transcripts are often not annotated correctly. Therefore, we combine the coverage of overlapping first and last exon groups. Isoforms are then discarded based on two values: the coverage per base and the number of covered bases. If both values are below user defined thresholds (e.g. 3 reads and 70% coverage) in all conditions, the isoforms including the exon are discarded. In the last stage, exon groups are recalculated based on the remaining isoforms and exon coverages are calculated (see below for the calculation of exon coverages). The adjusted coverage values are then used to remove isoforms with exon coverages below the previously defined coverage per base threshold. Again, coverage for all overlapping first and all overlapping last exons is combined.

**2.4 Calculation of exon coverages**

**Supplementary Figure S6**A summarizes the calculation of adjusted exon coverages for an example exon group illustrated in Supplementary Figure S6B. First, raw coverage for the exon group is obtained and exon coverage per base values are calculated and modified by the previously calculated size factors. Exons are considered too similar if they differ in 50 bp or less and are treated as one exon. These exons get the same coverage value (ambiguous exons). Next, each position in the exon group is assigned to exactly one exon, starting with the exon with the highest coverage per base value (Supplementary Figure S6C). Exon coverages are then recalculated based on the unique positions assigned to the exon. Final exon coverage values are then calculated by interrogating each position in the exon group (Supplementary Figure S6D). Coverages for each exon overlapping the given position are sequentially subtracted from the total coverage, beginning with the lowest covered exon at that position. The subtracted value corresponds to the fraction of the exon’s unique coverage to the maximum coverage of the exon group. While unique exon coverage could be calculated based on bases unique to the exon, this also works when there are no unique regions. However, our approach is still not perfect and might not always return the correct expression value for an exon. Especially in cases where there is a retained intron and multiple exon variants for the flanking exons. In these cases, coverage is more likely to be assigned to the exons than to the retained intron because it is biased by the, usually, higher exonic coverage.


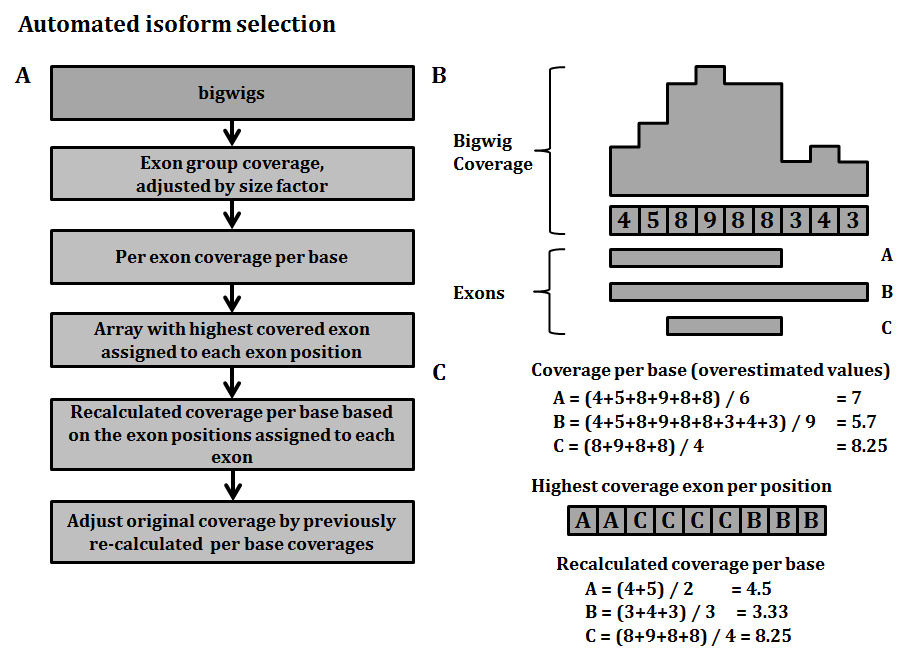


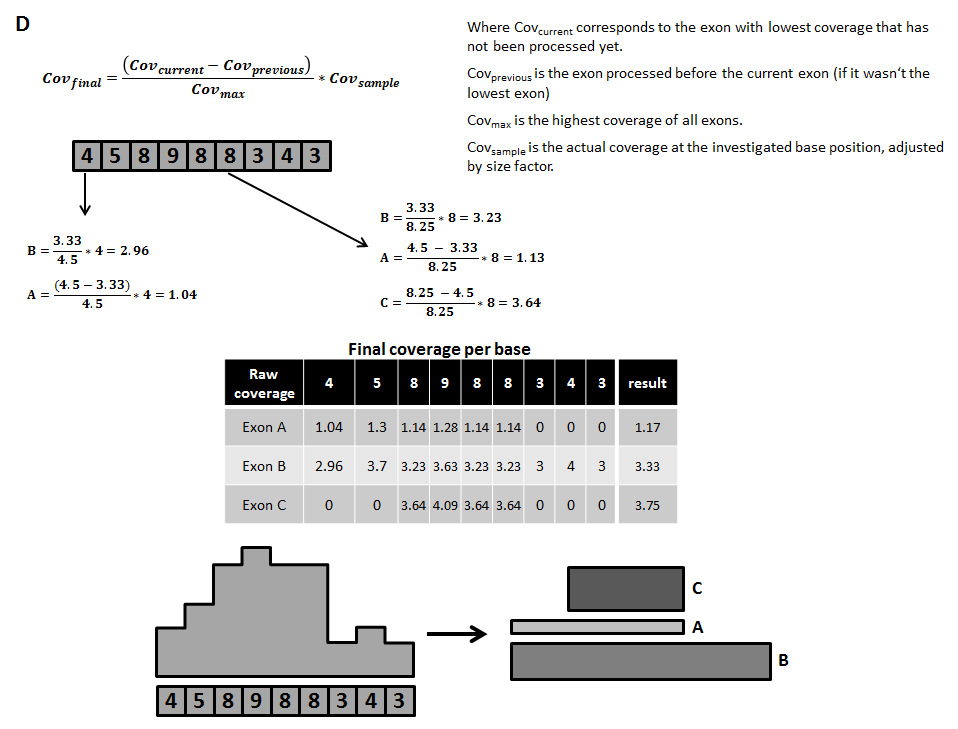


Supplementary Figure S6 - Workflow of the automated isoforms selection part 1: Generating unique expression values per exon. A Overview of the workflow. B Bigwigs are used for obtain the coverage across the whole exon group (an exon group contains all overlapping exons at a given locus). C A simple coverage per base value is calculated and used for ranking each exon. This value is usually an overestimation of the expression. Subsequently each position in the exon group is assigned to exactly one exon, starting with the highest expressed exon. Exon coverages are then re-calculated based on the positions assigned to each exon. D Final coverage is then obtained by multiplying the coverage at a given position by the proportion of each isoform to the total coverage at that position.

**2.5 Detection of alternatively spliced exons**

All remaining isoforms are tested for alternative splicing exploiting two different features of alternatively spliced exons. First, we expect alternatively spliced exons to show changes in the coverage and second, alternative splicing should be supported by the number of split reads supporting the exon inclusion and exclusion.

The coverage ratio test is performed for all condition combinations. However, conditions that do not express any of the exon groups (defined by the median coverage value per exon group and the coverage per base threshold) are omitted as this would only report differential expression rather than alternative splicing. For all exons and exon groups coverage ratios are calculated for each base (Supplementary Figure S7). Exon group ratios are stored as mean coverage ratio values. The coverage ratios obtained for each exon are then tested against the mean coverage ratios of all exon groups using a two-sided heteroscedastic t-test, excluding the exon groups that contain the exon of interest.


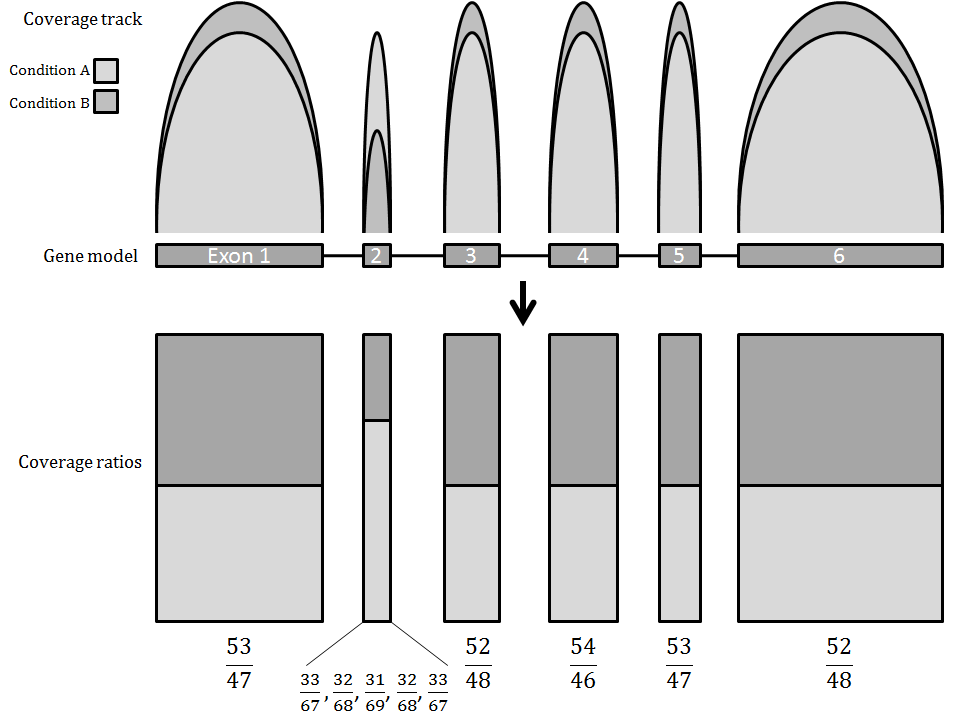


Supplementary Figure S7 – Calculation of coverage ratio changes. Shown at the top is a schematic illustration of the expression profile of a 6 exon gene in two different conditions A (light grey) and B (dark grey). Below is a representation of the coverage ratio between the two conditions. Numbers correspond to mean exon coverage ratios, except for the (query) exon 2 where coverage ratios for each base are shown. The second exon shows reduced expression in the condition A that is reflected by a coverage ratio change shifting the ratio towards a higher inclusion in condition B**.** Coverage ratios are calculated for each nucleotide of all exons and exon groups. Afterwards, a two-sided heteroscedastic t-test is used to assess the significance of the coverage ratio difference of a specific exon to the mean coverage ratios of all exon groups that do not contain this exon.

In a last step we combine the results from the coverage ratio and PSI score calculation to report the most likely candidates of alternative splicing. We always use the PSI score with the best p-value. However, if any junction used to calculate the best PSI score does not fit the gene annotation file a second entry will be generated based on the best PSI score that includes known junctions only. Manananggal will also report alternative splicing events that are supported by coverage ratio or PSI score only, however, these events have a higher chance of being false positives. Potential exon skipping events that show changes in the coverage ratio and PSI score are highlighted in red on the meta exon track in the viewer. Other types of alternative splicing events are not highlighted, but still appear in the temporary result list in the viewer. The example below explains why we chose not to highlight other types of alternative splicing events (e.g. alternative terminal exons).


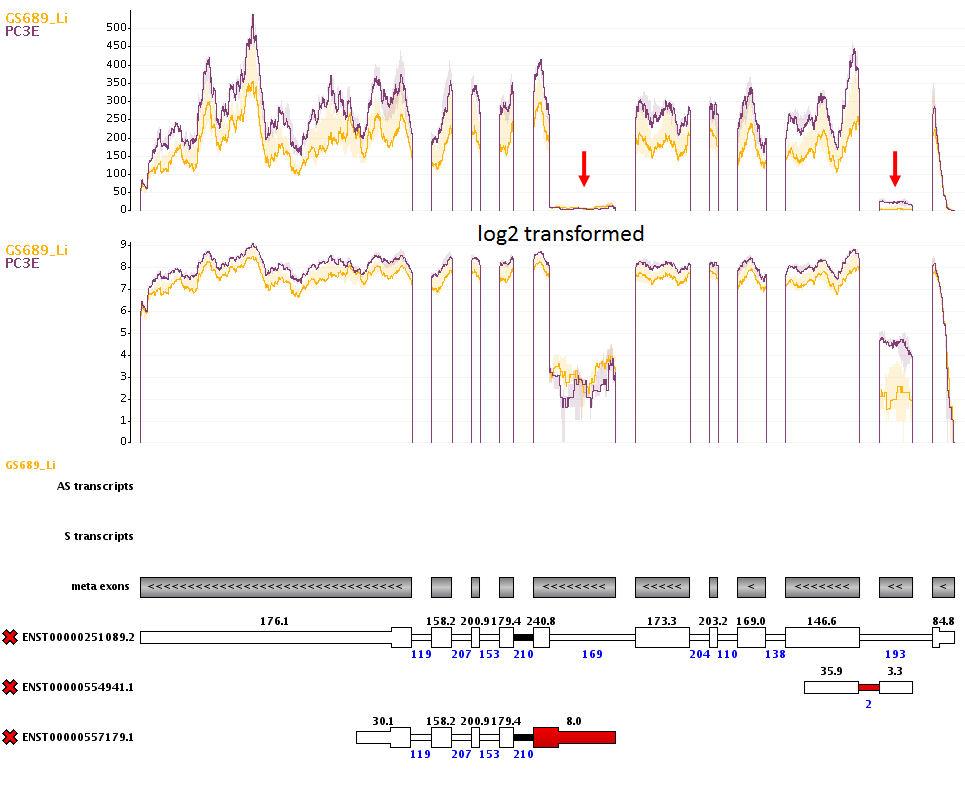


Supplementary Figure S8 - The picture above shows the gene ANGEL1, which might have a (weak) exon skipping event affecting the second exon. However, due to incomplete annotation of the second isoform and very low expression of the first exon of isoform three, Manananggal also reports an alternative transcript start event. It is an inherent problem of first and last exons that may act as central exons for other isoforms. These exons might appear expressed due to unprocessed transcripts or other sources of noise and can have high junction read counts. Therefore, genes with many incompletely annotated transcripts and lowly expressed transcripts with non-exclusive start exons tend to generate many false positive alternative splicing events that would result in many highlighted exons. Since this would not be very informative, we decided to highlight only exon skipping events that are easier to detect.

**2.6 Alternative terminal exons (ATEs)**

Alternative start or end exons are identified in a similar fashion to exon skipping events with some changes in the PSI score calculation. Instead of an inclusion and exclusion junction, Manananggal uses two junctions linked to different annotated start or end exons to calculate PSI score changes.

Further, Manananggal discriminates between events that identified an alternative start or end exon via a junction that is specific to that start or end exon and junctions that are shared with middle exons (Supplementary Figure S9).


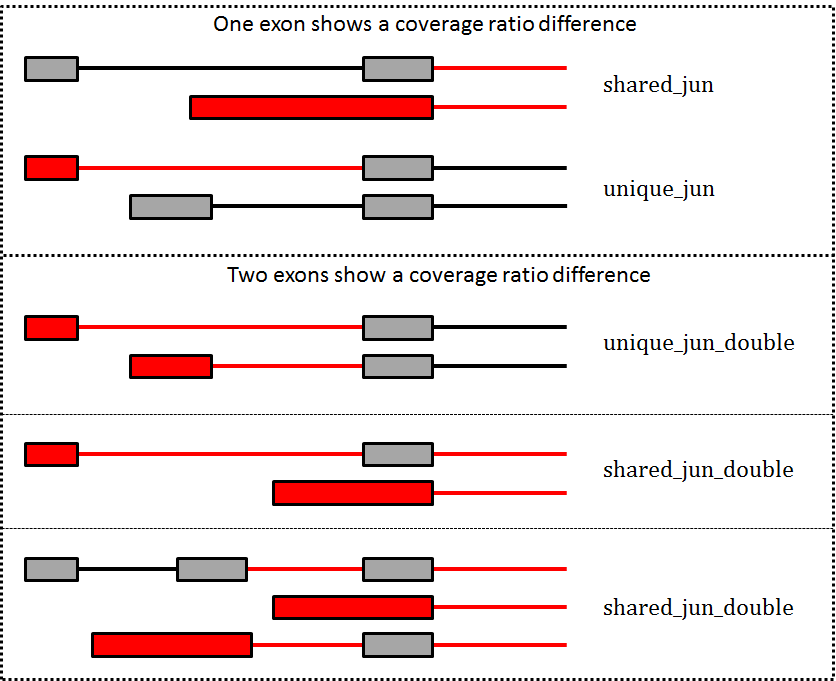


Supplementary Figure S9 – Sub types of alternative terminal exons. ATEs are discriminated by available evidence. All ATEs require a PSI difference between two annotated start of end exons. These exons either have a unique junction that only connects to that particular start or end exon (categorized as ‘unique’) or they share a junction with other exons that are not terminal exons (‘shared’). If only one exon shows are coverage ratio change, the event is categorized as either ‘shared-jun’ or ‘unique-jun’. If both start or both end exons show a coverage ratio change the ATEs are classified as ‘double’.

**2.7 Exon extensions (EEs) and retained introns (RIs)**

Exon extensions are currently only detected via PSI changes (Supplementary Figure S10). Manananggal screens for two junctions that connect to two different positions in the same exon. Due to mapping errors, the number of results in this category can be very high for low *junction coverage* thresholds. To improve the results, we plan to discriminate EEs by length and add information of coverage ratio changes to larger exon extensions. However, small EEs (~10-20 bp differences) will probably still rely on the PSI changes alone.

Retained introns are solely detected via coverage ratio changes. Additional information of exon-intron spanning reads is currently not used. In a future version we would like to include this information to improve the results.


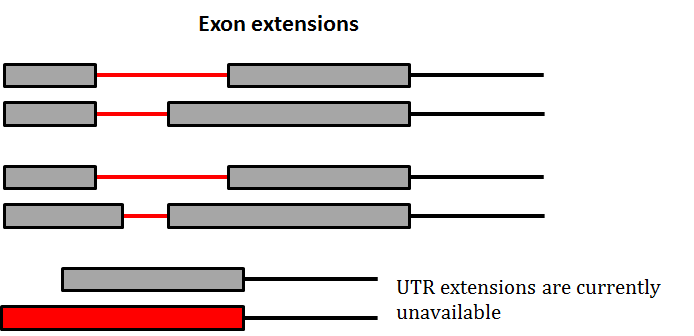


Supplementary Figure S10 – Exon extension events. Currently, Manananggal relies on PSI changes to identify exon extensions. Consequently, detection of UTR extensions is current unavailable.

Supplementary chapter III – In-depth Analysis of exon skipping events

3.1 Introduction

In this chapter we focus on the analysis of exon skipping event. Please note that only rMATS and Manananggal allow filtering for these events. For Cuffdiff and DEXSeq we used the whole lists that also contain other types of alternative splicing events. The advantage of looking at exon skipping events is that they should be the easier to detect than other types of splicing. Most exon skipping events should show a distinct difference in the coverage relative to other exons of the gene and each exon that is or is not skipped is often supported by two junctions (inclusion junctions). Alternative start or end exons for example are only supported by a single junction and sometimes share this junction with a non-terminal exon (e.g. in the gene *SIGIRR*).

3.2 Data sets

We used two different sets of genes for each tool that we investigated. The first set uses lenient criteria and thus probably includes a larger number of false positives. It consists of all results from rMATS, Cuffdiff and DEXSeq with a simple p-value < 0.05. For Manananggal we used exon skipping events with ‘combined’ evidence (i.e. they were identified by PSI changes and coverage ratio changes) and a small minimum coverage ratio of 0.05 (i.e. the coverage ratio of an accepted alternative exon must differ by 5% compared to the remaining exons of the gene).

For the second set we used more stringent criteria. Only results with a FDR < 0.05 were included from rMATS, results with an adjusted p-value < 0.1 from DEXSeq and all results that were flagged as “significant” from Cuffdiff. For Manananggal we increased the minimum coverage ratio threshold to 0.15 to focus on results with larger changes.

3.3 Venn diagrams

We calculated different Venn diagrams for the overlap of all genes in the lenient result lists and all genes in the stringent result lists. Regarding the lenient result lists (Supplementary Figure S11 A), most exon skipping candidate genes identified by rMATS and Manananggal were also included in the result list of at least one other tool. Keep in mind that Cuffdiff and DEXSeq might include the gene for a different reason (e.g. because they identified an alternative transcript start or end exon). More than 79% of genes reported by Manananggal were also detected by at least two other tools, whereas only 36% of genes reported by rMATS were also reported by at least two other tools. However, 55% of genes reported by rMATS were also included in the DEXSeq result lists but in none of the other tools. While being a little smaller, these numbers did not change substantially for the stringent result lists (Supplementary Figure S11 B and C). The difference between **B** and **C** is that we ran Manananggal once without (**B**) and once with (**C**) the optional parameter to exclude first and last exons in the step where it decides whether it should discard an isoform due to insufficient coverage. Numbers indicate that using the optional parameter is beneficial to the results.


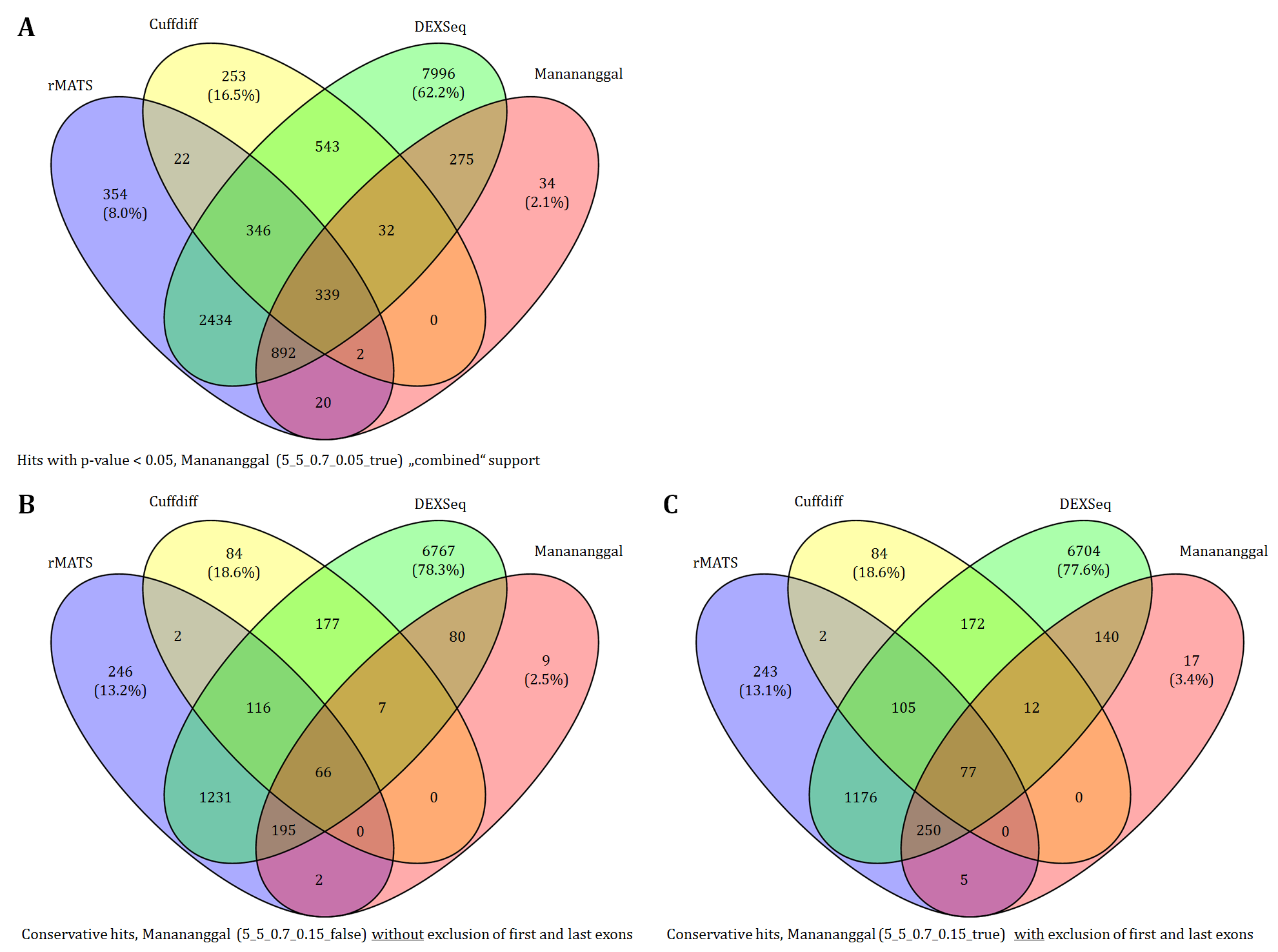


Supplementary Figure S11 - Venn diagrams showing the overlap of genes identified by Manananggal, DEXSeq, rMATS and Cuffdiff by to be alternatively spliced. A Comparison of genes that were reported to have AS events by DEXSeq, rMATS or Cuffdiff (p-value < 0.05) to AS events reported by Manananggal (parameters: 5_5_0.7_0.05_true) with combined support of coverage changes and PSI score. The overall overlap for all tools is small considering the total number of events each tool reported, but most AS candidates identified by Manananggal, rMATS and Cuffdiff were also detected by at least one other tool. About 3% of candidates detected by Manananggal were not detected by any other tool. DEXSeq is on the other extreme end of the scale (>60% of the results were only reported by DEXSeq), but this can partially be explained by the fact that the results from DEXSeq and Cuffdiff cannot be filtered for exon skipping events specifically. Therefore, their result lists include other types of alternative splicing events that are not included in the “skipped exon” results from rMATS and Manananggal. B+C Comparison of conservative AS calls from DEXSeq (p-adjusted < 0.1), rMATS (FDR < 0.05), Cuffdiff (flagged as significant) and Manananggal (5_5_0.7_0.15) including (B) or discarding first and last exons (C). Counterintuitively, the fraction of AS candidate genes that were detected by only one tool increased for all tools and the number of AS events reported by all tools decreased when using more strict criteria. However, Manananggal, rMATS and DEXSeq still have a substantial overlap. Most AS candidate genes reported by Manananggal were also detected by rMATS and/or DEXSeq. Exclusion of first and last exons had a beneficial effect on the number of shared AS events, but also increased the number of Manananggal exclusive events slightly (+0.9%).

**3.4 False positive exon skipping events based on gene symbols**

To assess the number of false positive exon skipping events we picked the top 100 candidate genes from each tool and compared this list to the lenient result lists of all other tools (Supplementary Figure S12). All candidate genes from rMATS and Manananggal were also included in the result lists of the other tools and 97 of 100 genes were also reported by either all tools (rMATS: 69; Manananggal: 38) or rMATS, DEXSeq and Manananggal (rMATS: 28; Manananggal: 41). Even though Cuffdiff and DEXSeq included other types of alternative splicing events as well and we only included exon skipping events in the rMATS and Manananggal result lists, a larger number of their best 100 candidate genes were also included by at least two other tools (Cuffdiff: 65; DEXSeq: 68).

Because there were no genes among the top 100 candidates exclusively reported by rMATS or Manananggal we used the list of genes from the previous Venn diagrams that were reported by only a single tool as potential false positives (Supplementary Figure S11 C).

3.4.1 rMATS false positive exon skipping events

We visually inspected the best 20 (of 243) candidate genes for exon skipping that were exclusively detected by rMATS (Supplementary Table S7, Supplementary Figure S13). Most of the results point towards exon skipping events that are mainly supported by small differences in junction spanning read counts.

3.4.2 Cuffdiff false positive alternative splicing events

The top 20 (of 84) genes (excluding hits with multiple gene identifiers and sorted by p-value) were manually investigated (Supplementary Table S8, Supplementary Figure S14). Most results did not pass visual inspection, with many events pointing to genes with only one or two exons and differentially expressed genes.

3.4.3 Manananggal false positive alternative splicing events

We visually inspected all 17 exon skipping candidate genes detected exclusively by Manananggal (Supplementary Table S10, Supplementary Figure S15). Most results point towards small changes in the splicing pattern, similar to the potential false positives of rMATS. Because the coverage and read numbers supporting these events are low, we expect most of them to be indeed false positives.

3.4.4. DEXSeq false positive alternative splicing events

Most of the visually inspected top 20 (of 6704) alternative splicing events (ordered by adjusted p-value) detected exclusively by DEXSeq turned out to be good alternative splicing candidates (Supplementary Table S9). However, none of them represented an exon skipping event. Some false positives are included that can be attributed to expression of overlapping transcripts in antisense direction.

3.4.5 Summary

Most exon skipping candidate genes that were exclusively detected by rMATS or Manananggal showed only small changes and many of them require a close look at the read counts of junction spanning reads to identify the difference. Considering the low sample number (3 vs. 3) we expect many of these results to be real false positives. However, only a small fraction of genes were exclusive to either rMATS (243 genes; 13.1% of total) or Manananggal (17; 3.4%). Cuffdiff had relatively more false positive candidates (84; 18.6%) and in contrast to rMATS and Manananggal most of them did not even show weak signs of alternative splicing. This approach is not adequate to assess the false positive rate of DEXSeq. Therefore it is not too surprising that most of the DEXSeq potential false positives were actually true positives referring to other types of alternative splicing. Some results were influenced by overlapping antisense transcripts and were thus indeed false positives.


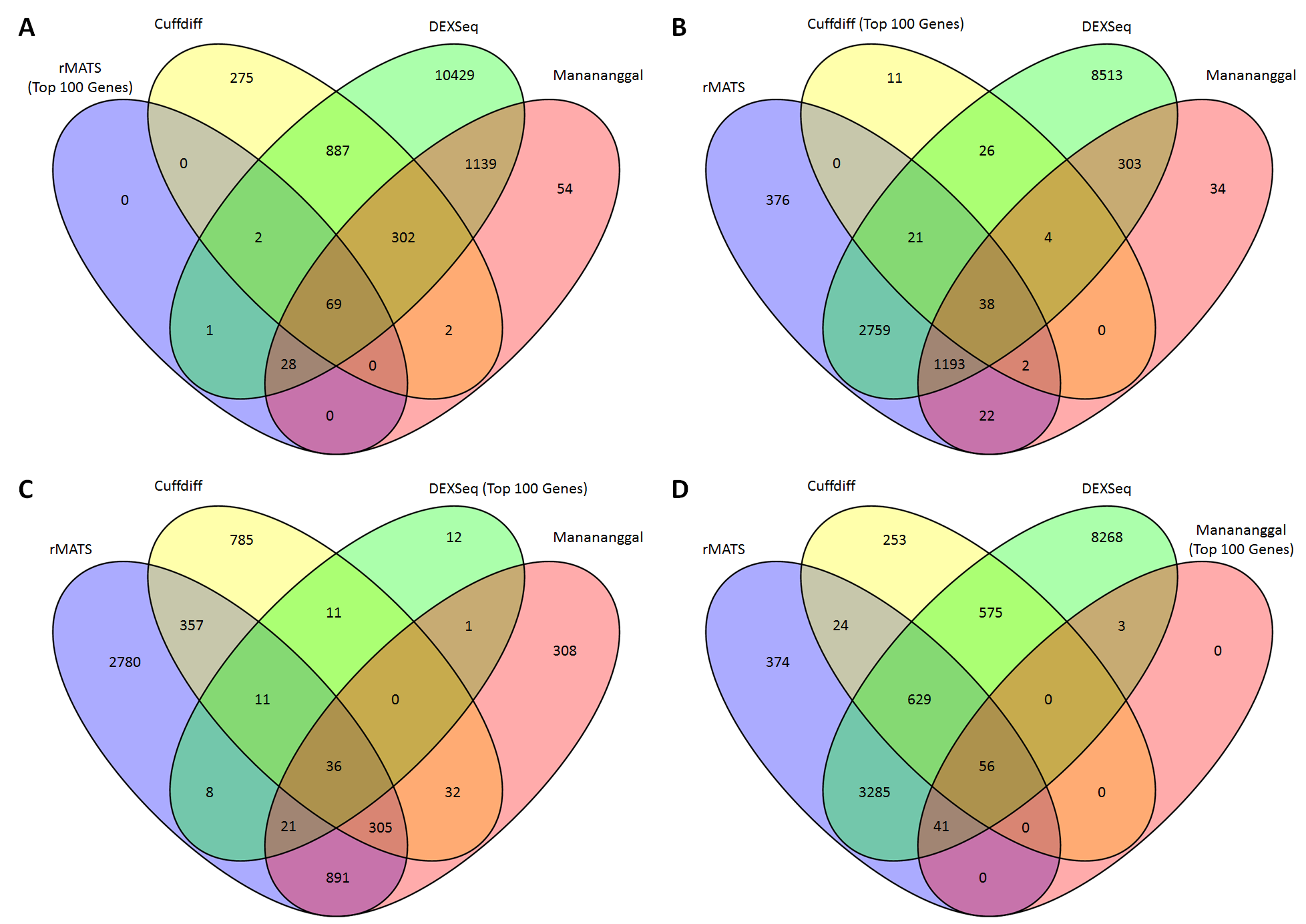


Supplementary Figure S12 - Comparison of the top 100 genes with alternative splicing events reported by rMATS, Cuffdiff, DEXSeq and Manananggal to all alternative splicing events of the other tools. A p-value cutoff < 0.05 was used for DEXSeq, rMATS and Cuffdiff to get the list of ‘all’ alternative splicing events. For Manananggal we used the result list with lower ratio cutoff value (5_5_0.7_0.05_true) for the ‘all’ alternative splicing events list and higher cutoff value (5_5_0.7_0.15_true) for the top100 genes. rMATS (A) and Manananggal (D) had no exclusive genes in their top 100 gene candidates for alternative splicing and shared 69% (rMATS) and 56% (Manananggal) with all other tools, and 97% with at least two other tools. B Eleven genes were exclusively reported by Cuffdiff to be subject of alternative splicing, 38% of reported genes were also reported by all other tools and 65% were shared with at least two other tools. C Similar to Cuffdiff, 36% of the top 100 genes with alternative splicing events predicted by DEXSeq were also reported by all other tools and 68% were detected by at least two other tools.

None of the genes exclusively reported by Cuffdiff showed signs of alternative splicing upon visual inspection. Most results pointed to genes with only 1 isoform or genes with single exon isoforms, thus these might be gene annotation specific false positive results. The remaining result pointed towards *COX19* that shows a switch from a large to a short isoform affecting a larger number of exons. However, the additional exons are shared with an overlapping gene in sense direction and no split-reads were detected that support a connection to COX19, therefore this result is very likely a false positive.

The exclusive DEXSeq results included genes with extended 5’ or 3’ UTRs (*CAPS*, *RPS23*,), alternative transcript starts (*SPTBN1*, *SET*, *CUX1*, *COMT*, MYO5B, ), alternative transcript end (*BICC1*), exon extension (*PFN2*) and a false positive due to an overlapping anti-senes transcript (ENSG00000267577, *EML5*). Due to a very suspicious coverage plot it is impossible to tell whether a splicing event in *POM121B* is differential between the samples. Therefore, most exclusive DEXSeq results represent valid AS events, they were simply not exon skipping events.

**Supplementary Table S7 – rMATS potential false positive exon skipping events.** Visual inspection of the top 20 (of 243) exon skipping events detected exclusively by rMATS. Most results point towards exon skipping events that are mainly supported by small differences in junction spanning read counts.

| **gene** | **p-value** | **FDR** | **incl. Level difference** | **exon position** | **valid after vis. inspection valid** | **comment** |
| --- | --- | --- | --- | --- | --- | --- |
| ***CAMK1*** | 0.00E+00 | 0.00E+00 | 0.094 | chr3:9802339-9802452 | no | overlapping antisense transcript |
| ***ATP5I*** | 0.00E+00 | 0.00E+00 | -0.050 | chr4:667700-667755 | (**yes**) | Small change |
| ***DYNLL2*** | 0.00E+00 | 0.00E+00 | -0.037 | chr17:56164442-56164583 | (**yes**) | Small change |
| ***ABHD14A-ACY1*** | 0.00E+00 | 0.00E+00 | -0.103 | chr3:52019222-52019287 | no |  |
| ***TEX22*** | 0.00E+00 | 0.00E+00 | 0.215 | chr14:105915695-105915746 | (no) | Exon belongs to *MTA1* |
| ***DUSP6*** | 8.88E-16 | 2.54E-13 | 0.052 | chr12:89744364-89744802 | (**yes**) | Small change |
| ***ETHE1*** | 2.89E-15 | 7.85E-13 | -0.104 | chr19:44030352-44030501 | **yes** |  |
| ***SLC7A6*** | 2.61E-14 | 6.53E-12 | 0.092 | chr16:68330529-68330713 | (**yes**) | Small change |
| ***CNN2*** | 5.17E-14 | 1.25E-11 | -0.133 | chr19:1036065-1036245 | no |  |
| ***B4GALT3*** | 2.23E-13 | 5.09E-11 | -0.054 | chr1:161146701-161146896 | **yes** |  |
| ***APEX2*** | 7.35E-13 | 1.60E-10 | 0.100 | chrX:55028683-55028864 | (no) | Very small change |
| ***RETSAT*** | 9.68E-13 | 2.07E-10 | 0.042 | chr2:85578802-85578985 | **yes** |  |
| ***SLC52A2*** | 1.30E-11 | 2.49E-09 | 0.037 | chr8:145584249-145584373 | (no) | Very small change |
| ***ZSCAN16-AS1*** | 3.10E-11 | 5.75E-09 | -0.168 | chr6:28093400-28093597 | no | Antisense transcript |
| ***PIGF*** | 9.93E-11 | 1.76E-08 | -0.112 | chr2:46818941-46819064 | **yes** |  |
| ***CDC45*** | 1.84E-10 | 3.16E-08 | 0.090 | chr22:19502487-19502571 | (**yes**) | Small change |
| ***ZNF532*** | 7.24E-10 | 1.14E-07 | 0.222 | chr18:56532535-56532617 | (no) | Very small change |
| ***R3HDM2*** | 1.69E-09 | 2.55E-07 | 0.089 | chr12:57691110-57691170 | (**yes**) | Small change |
| ***SEMA7A*** | 1.83E-09 | 2.74E-07 | 0.063 | chr15:74710608-74710650 | (**yes**) | Small change |
| ***COX16*** | 2.85E-09 | 4.19E-07 | -0.067 | chr14:70809374-70809446 | (**yes**) | Small change |


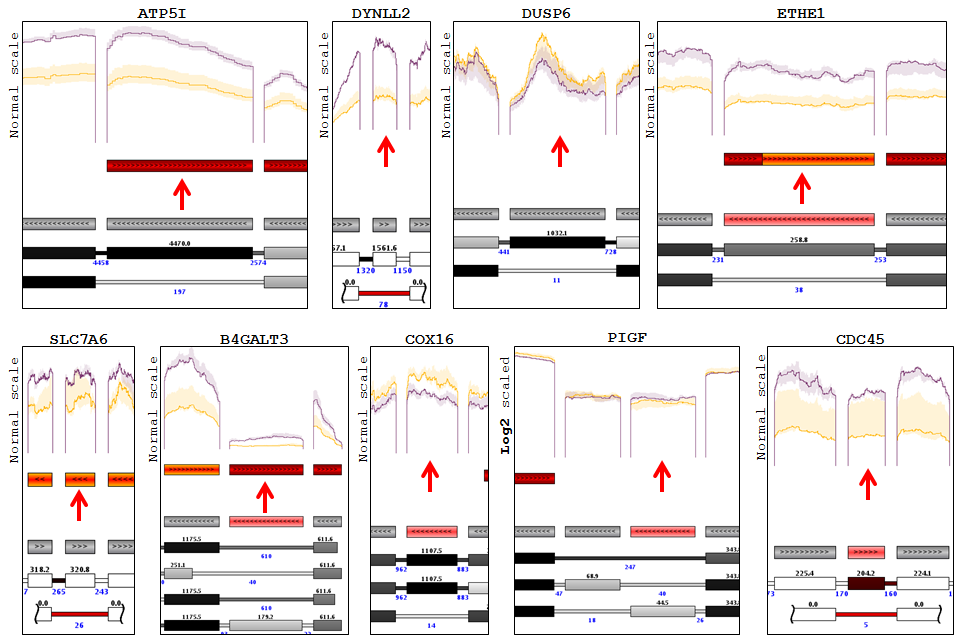


Supplementary Figure S13 - rMATS potential false positive exon skipping events. Shown are 9 exon skipping events exclusively reported by rMATS (considering only conservative results) that might refer to real exon skipping events. Most events show no visible change in coverage and require detailed inspection of junction spanning reads. *DYNLL2*, *CDC45* and *SLC7A6* include novel junctions (i.e. the splicing event is not annotated yet).

**Supplementary Table S8 - Cuffdiff potential false positive alternative splicing events.** The top 20 (of 84) genes (excluding hits with multiple gene identifiers and sorted by p-value) were manually investigated. Most results did not pass visual inspection, with many events pointing to genes with only one or two exons and differentially expressed genes.

| **gene** | **p-value** | **significant** | **valid after vis. inspection** | **comment** |
| --- | --- | --- | --- | --- |
| *AC004448.5* | 0.00005 | Yes | No | not expressed |
| *TPPP* | 0.00005 | yes | No | Overlapping antisense transcript |
| *AC005481.5* | 0.00005 | Yes | No | 2-exon gene |
| *HNRNPA3P3* | 0.00005 | Yes | No | 1-exon gene |
| *HLA-Z* | 0.00005 | Yes | No | 1-exon gene |
| *TFF3* | 0.00005 | Yes | No |  |
| *MUM1L1* | 0.00005 | Yes | No |  |
| *ASCL5* | 0.00005 | Yes | No | 2-exon gene |
| *RP11-909N17.2* | 0.00005 | Yes | No |  |
| *RHOV* | 0.00005 | Yes | No | differential expression |
| *HIPK3* | 0.00005 | Yes | **Yes** | weak candidate |
| *GNAZ* | 0.00005 | Yes | No |  |
| *CTF1* | 0.00005 | Yes | No |  |
| *GRB14* | 0.00005 | Yes | No | differential expression |
| *LINC00327* | 0.00005 | Yes | No |  |
| *RYR2* | 0.00005 | Yes | No | Not expressed |
| *SYT14* | 0.00005 | Yes | **Yes** | Exon extension |
| *GPRIN2* | 0.00005 | Yes | No | differential expression |
| *NUDT19* | 0.00005 | Yes | No |  |
| *SWSAP1* | 0.00005 | Yes | No | 2-exon gene |


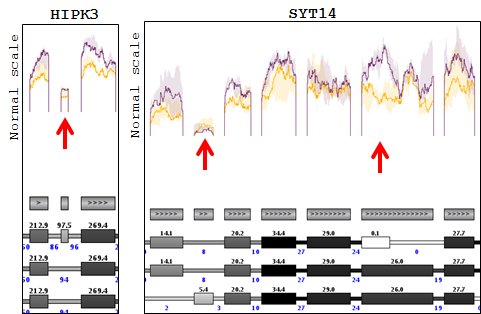


Supplementary Figure S14 - Cuffdiff potential false positive exon skipping events. Shown are 2 exon skipping events exclusively reported by Cuffdiff (considering conservative only results) that might refer to real exon skipping events.

**Supplementary Table S9 - Alternative splicing events exclusively reported by DEXSeq.** Most of the inspected top 20 alternative splicing events (ordered by adjusted p-value) detected exclusively by DEXSeq turned out to be good alternative splicing candidates. However, none of them represented an exon skipping event. Some false positives are included that are usually caused by overlapping transcripts in antisense direction.

| **gene_symbol** | **exon** | **pvalue** | **padj** | **valid after vis. inspection** | **comment** |
| --- | --- | --- | --- | --- | --- |
| *SPTBN1* | chr2:54753509-54753703 | 0.00E+00 | 0.00E+00 | **Yes** | alternative transcript start |
| *MUC5AC* | chr11:1221664-1222364 | 2.8575E-279 | 2.3486E-275 | **Yes** | Novel transcripts |
| *EPHA1* | chr7:143087382-143087738 | 3.2099E-277 | 2.5844E-273 | No | Antisense transcript |
| *FKBP1A* | chr20:1352422-1352719 | 1.4373E-263 | 1.0904E-259 | (**Yes**) | 3’-UTR extension |
| *CTD-2587H24.5* | chr19:55688452-55688908 | 1.0549E-218 | 6.3056E-215 | No | Antisense transcript |
| *CUX1* | chr7:101926313-101926770 | 1.279E-218 | 7.531E-215 | **Yes** | alternative transcript start |
| *BAG1* | chr9:33252477-33255022 | 6.7809E-212 | 3.6646E-208 | (**Yes**) | 3’-UTR extension |
| *GPRC5C* | chr17:72445649-72446719 | 2.9958E-209 | 1.5759E-205 | **Yes** | Alt. start/end |
| *CAPS* | chr19:5911718-5914231 | 1.4275E-205 | 7.3139E-202 | (**Yes**) | 5’-UTR extension |
| *SET* | chr9:131446059-131446270 | 1.7715E-180 | 8.1267E-177 | **Yes** | alternative transcript start |
| *CTPS2* | chrX:16606429-16607671 | 4.1087E-170 | 1.8011E-166 | **Yes** | An unannotated start exon creates a shorter transcript. |
| *COMT* | chr22:19950050-19950069 | 9.3581E-168 | 3.9276E-164 | **Yes** | alternative transcript start |
| *COL1A1* | chr17:48277309-48277552 | 3.657E-165 | 1.5187E-161 | **Yes** | 5’ exon extension |
| *CSNK1A1* | chr5:148885010-148885045 | 1.0184E-164 | 4.0999E-161 | **Yes** | 3’ exon extension |
| *C2orf68* | chr2:85833777-85835406 | 3.25E-153 | 1.1551E-149 | **Yes** |  |
| *KRT8* | chr12:53298837-53298856 | 3.2062E-147 | 1.0999E-143 | (**Yes**) |  |
| *POM121B* | chr7:72710927-72712437 | 2.6278E-144 | 8.7859E-141 | No | Mapping problems |
| *RPS23* | chr5:81569177-81570342 | 9.3091E-143 | 3.0103E-139 | **Yes** | 3’-UTR extension |
| *BZW1* | chr2:201677079-201677216 | 2.6894E-140 | 8.2893E-137 | **Yes** | alternative transcript starts |
| *EML5* | chr14:89080065-89081173 | 2.2317E-132 | 6.5218E-129 | No | Antisense transcript |

**Supplementary Table S10 – Manananggal potential false positive exon skipping events.** We visually inspected all 17 exon skipping candidate genes detected exclusively by Manananggal. Most results point towards small changes in the splicing pattern. Because the coverage and read numbers supporting these events are low, we expect most of them to be false positives.

| **Gene** | **PSI**  **p-value** | **Coverate**  **Ratio**  **change** | **position** | **Novel junction** | **valid** | **comment** |
| --- | --- | --- | --- | --- | --- | --- |
| ***POLB*** | 2.26E-03 | 0.17 | chr8:42218614-42218720 | yes | no | Low exon expression |
| ***EXTL2*** | 2.52E-03 | -0.15 | chr1:101343950-101344003 |  | (**yes**) | Small change |
| ***PANX2*** | 2.72E-03 | 0.22 | chr22:50613861-50613911 | yes | **yes** |  |
| ***ICT1*** | 5.42E-03 | 0.16 | chr17:73014681-73014789 | yes | (no) | Very small change |
| ***RP11-73M18.2*** | 5.70E-03 | -0.2 | chr14:104037960-104038157 | yes | No |  |
| ***COPB1*** | 9.94E-03 | -0.15 | chr11:14516119-14516273 |  | No |  |
| ***CBWD7*** | 1.14E-02 | -0.16 | chr9:42712550-42712618 | yes | No |  |
| ***RP1-309F20.3*** | 1.15E-02 | -0.17 | chr20:57442541-57442627 | yes | No |  |
| ***ZNF79*** | 1.18E-02 | -0.18 | chr9:130190831-130190939 |  | **yes** | Small change |
| ***CMC2*** | 1.21E-02 | -0.2 | chr16:81031890-81031956 |  | (No) | Very small change |
| ***AP000347.2*** | 1.59E-02 | -0.16 | chr22:24028977-24029182 |  | No | Overlapping transcripts |
| ***ARL6IP6*** | 2.36E-02 | 0.18 | chr2:153589045-153589094 | yes | ? |  |
| ***PHF20*** | 3.10E-02 | -0.3 | chr20:34388016-34388095 |  | **yes** | Small change |
| ***CPEB2*** | 3.44E-02 | -0.18 | chr4:15042088-15042111 |  | **yes** |  |
| ***LATS1*** | 3.57E-02 | 0.15 | chr6:150018228-150018322 |  | **yes** |  |
| ***MECR*** | 3.79E-02 | 0.16 | chr1:29527886-29528030 | yes | (**yes**) | Small change |
| ***OR2A1-AS1*** | 4.49E-02 | -0.23 | chr7:143951527-143951789 |  | no | Overlapping transcripts |


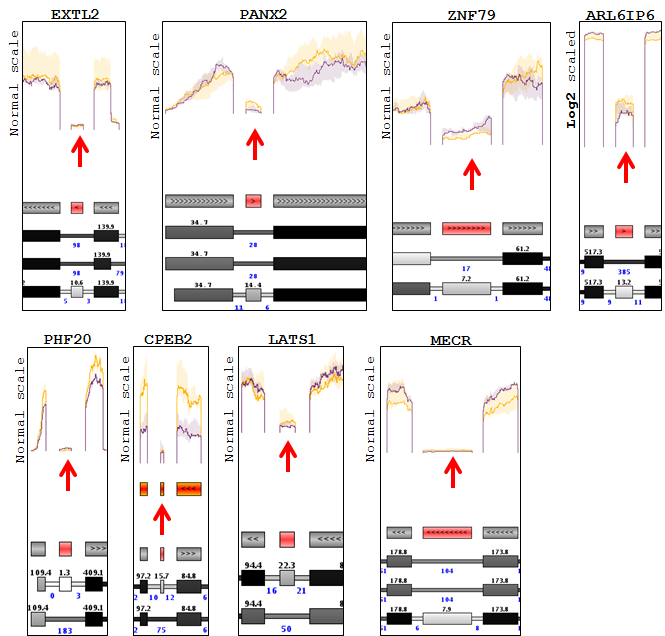


Supplementary Figure S15 - Manananggal potential false positive exon skipping events. Shown are 8 exon skipping events exclusively reported by Manananggal (considering only conservative results) that might refer to real exon skipping events, although they usually show only small coverage differences.

**3.5 False negative exon skipping events based on gene symbols**

To identify false negatives we used the list of genes that were reported by all but one tool as potential false positives (Supplementary Figure S11 C). These lists of potential false negatives are enriched for exon skipping events, because they always include the results from either rMATS or Manananggal that were filtered for exon skipping events.

3.5.1 – rMATS false negative exon skipping results

We manually inspected all 12 candidate genes detected by all tools but rMATS (Supplementary Table S11, Supplementary Figure S17). All genes showed signs of alternative splicing. However, three of them did not refer to an exon skipping event but to other types of alternative splicing. Several candidates showed strong differences and represent clear false negative results (*EYA4*, *FYB*, *OGDH*, *SLC37A2*, *STXBP5*, and *GK*)*.*

3.5.2 – Cuffdiff false negative exon skipping results

Cuffdiff did not include 250 AS candidate genes that were reported by all other tools. We sorted these genes by the Manananggal PSI p-value for results with combined evidence (coverage ratio change and PSI score change) and visually inspected the top 30 candidates (Supplementary Table S12, Supplementary Figure S18). Except for three results, all events appear to be valid alternative splicing events and actually refer to exon skipping events. Most of these genes had large p-values in the Cuffdiff result list.

3.5.3 – Manananggal false negative exon skipping results

Regarding the number of AS candidate genes missed, Manananggal lies in between rMATS and Cuffdiff (105 potentially false negative results). For several genes, rMATs and DEXSeq reported different exon positions. To get the highest chance of finding false negatives we therefore filtered the list of missed AS candidate genes by matching rMATS and DEXSeq position first and then ordered the list by rMATS p-value and DEXSeq p-value. Afterwards, we visually inspected the best 29 candidate genes (Supplementary Table S13, Supplementary Figure S19). All of the investigated genes appear to be valid alternative splicing candidates.

Except for two, all of them are also reported by Manananggal but did not appear in the conservative result list that was generated using a coverage ratio threshold of 15% (Missed results had a coverage ratio difference between 7% and 14.8%).

3.5.4 – DEXSeq false negative exon skipping results

DEXSeq did not miss any of the AS candidate genes, but this does not necessarily mean that it detected all exon skipping events. DEXSeq reported the largest number of results, thus also included the largest number of candidate genes. It is possible that a gene was included in the result list because of an exon extension or alternative transcript start or end exon, and not because it was referring to an exon skipping event.

3.5.5 - Summary

These above results indicate that rMATS and Manananggal (considering that it finds most of the ‘missed’ events using lower cutoffs) have low false negative rates concerning the detection of exon skipping events. In contrast, Cuffdiff appears to have a relatively high false negative rate because it missed many events that showed clear signs of alternative splicing.

3.6 – Comparison of exon skipping events based on chromosomal coordinates

To better understand the false negative rate of exon skipping events in DEXSeq we compared its results to those of Manananggal and rMATS based on base positions (Cuffdiff does not provide this information and was thus left out). Supplementary Figure S16 shows the pair-wise comparison of all result lists based on the exon positions. This was done once for overlapping events (because DEXSeq reports only exonic part positions) and once for ‘precise’ events (e.g. events that were reported with the exact same coordinates).

Most of the events (2130/2357) detected by Manananggal were included in the complete result list of rMATS (p-value < 0.05) and 945 of them were also included in the rMATS results with FDR < 0.05. Almost all hits were also matching the same coordinates. In comparison to the DEXSeq results, these numbers were slightly smaller. 1829 of 2357 events detected by Manananggal were overlapping events reported by DEXSeq (p-value <0.05) and 1572 of them were included in the conservative result list (p-adj < 0.1).About half of them were also matching to the exact same coordinates. Similarly, the overlap of significant (p-adj. < 0.1) DEXSeq hits with rMATS (p-value < 0.05) was 2145 and with significant rMATS results (FDR < 0.05) 1399. About 60% of these were also ‘precise’ hits.

3.6.1 – DEXSeq false negative exon skipping events based on chromosomal coordinates

Based on the above results, we generated a list of exon skipping events that were missed by DEXSeq to assess the false negative rate. 523 events were missed by DEXSeq compared to Manananggal (‘combined’ results) and 900 in the comparison of DEXSeq to rMATS (“FDR < 0.05”). This resulted in 55 events that were identified via the same coordinates by rMATS and Manananggal, but were missed by DEXSeq. The top 20 events (ordered by rMATS p-value) were visually investigated (Supplementary Table S14, Supplementary Figure S20). Most events show signs of alternative splicing, but several are referring to small changes that can only be identified by inspection of junction spanning reads. Therefore it is not surprising that DEXSeq missed most of them.


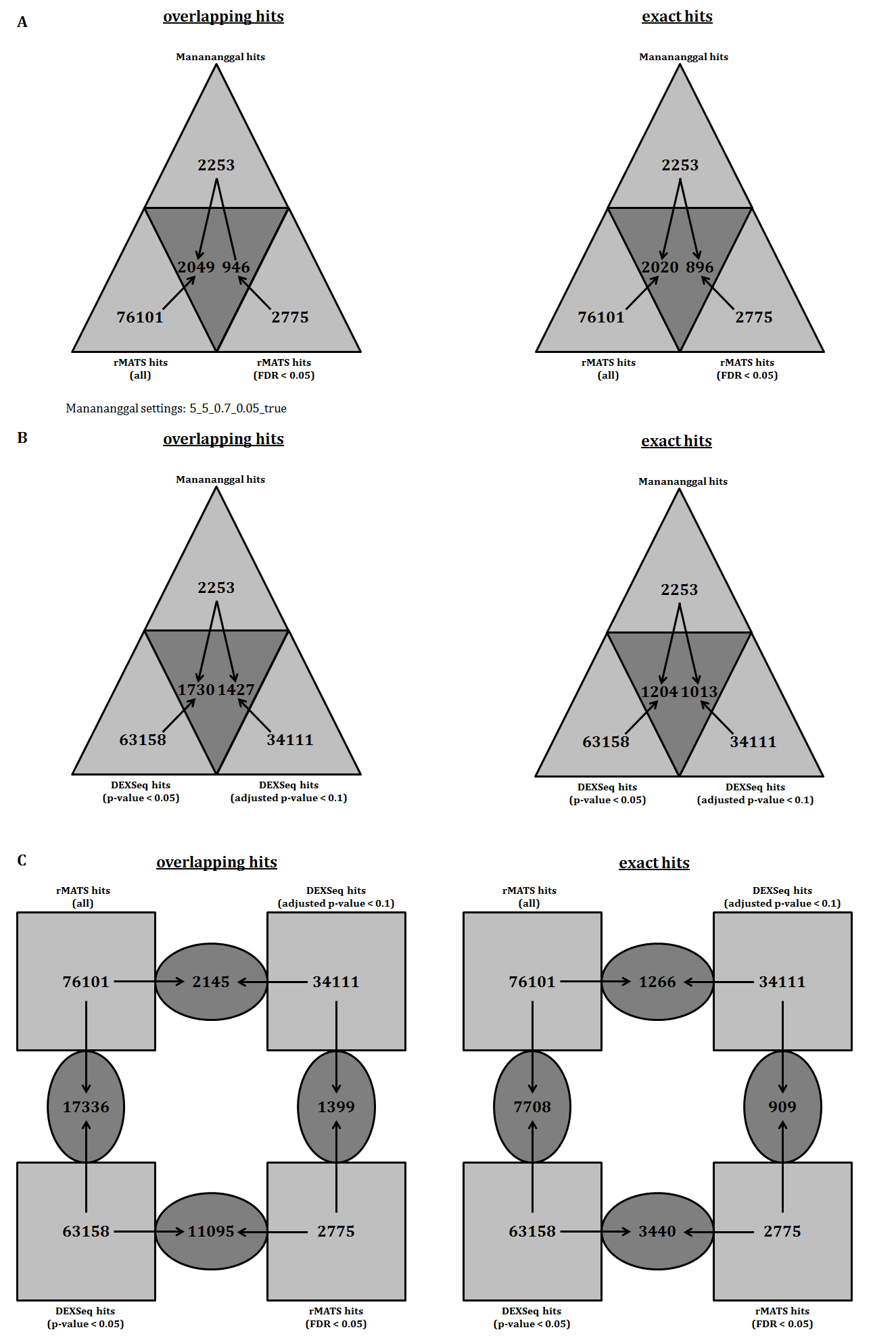


Supplementary Figure S16 – Venn diagrams showing the overlap of antisense events detected by Manananggal, rMATS and DEXSeq based on the position of reported AS events. Alternative splicing candidate lists of all three tools were compared in a pair-wise manner. We investigated the overlap of reported events using lax parameters (i.e. ignoring the FDR and adjusted p-value) and strict parameters (FDR < 0.05 for rMATS and adjusted p-value < 0.1 for DEXSeq). We used only Manananggal results that had coverage ratio support and a p-value for the PSI change < 0.05. We investigated how many events reported the exact same exon coordinates (right side) and how many were simply overlapping (left side).

A The overlap of rMATS and Manananggal reported AS candidates is very high considering all rMATS results. 90.9% (2049/2253) of Manananggal AS candidates were matching at least one AS candidate reported by rMATS and 98.6% (2020/2049) of them reported the exact same alternatively spliced exon. However, only 53.4% (1204/2253) of Manananggal AS candidates were reported with a FDR < 0.05 in rMATS. B About 76.8% (1730/2253) of Manananggal AS candidates were also detected by DEXSeq, which are fewer AS candidates than were also detected by rMATS. 69.6% (1204/1730) of overlapping events were referring to the exact same exons. 53.4% of overlapping events and 45.0% of exact matches had a p-adjusted value of <0.1 in DEXSeq. C The numbers in ellipses correspond to the number of matching rMATS AS candidates. Comparing all rMATS AS candidates to DEXSeq AS candidates with p-value < 0.05 resulted in an overlap of about 27.4% (17336/63158). 44.4% (7708/17336) of these were reporting the exact same exon, a value similar to the one achieved by Manananggal. Using the more conservative AS candidate lists from both tools (rMATS FDR < 0.05; DEXSeq adj. p-value < 0.01) resulted in an overlap of 50.4% (1399/2775), which is slightly less than are shared by Manananggal and DEXSeq (1730). The number of exact matches (909/2775; 32.8%) in the list of significant results from both tools is also very similar to the overlap of either tool to Manananggal. However, Manananggal reports fewer results in total.

Supplementary Table S11 – rMATS false negatives. We visually inspected 12 (of 12) genes with potential exon skipping events (SE) detected by all tools but rMATS. Most of them are good candidates of alternative splicing, however, some of them probably do not represent classical exon skipping events and therefore it is not surprising they were not included in the rMATS “skipped-exon” result list. However, several of these events are very likely exon skipping events that were missed by rMATS (e.g. *EYA4, FYB, OGDH, SLC37A2, STXBP5,* and *GK*).

| **gene symbol** | **exon position** | **valid after**  **visual inspection** | **comment** | **rMATS p‑value** |
| --- | --- | --- | --- | --- |
| *TH* | chr11:2187232-2187288 | (No) | Valid alternative splicing event, although it rather concerns expression of one short and one long isoform rather than an exon skipping event. | 1.00 |
| *PRR16* | chr5:119816864-119816988 | **Yes** | Weaker candidate | 0.44 |
| *SLC37A2* | chr11:124956100-124956156 | **Yes** |  | 1.00 |
| *OPRL1* | chr20:62723319-62723469 | **Yes** | Weaker candidate | 2.55E-03 |
| *GK* | chrX:30715849-30715866 | **Yes** | Weaker candidate | - |
| *EYA4* | chr6:133846153-133846253 | **Yes** |  | - |
| *EPB41L5* | chr2:120861636-120861664 | (No) | Alternative transcript end, not exon skipping | - |
| *OGDH* | chr7:44687043-44687133 | **Yes** |  | 0.61 |
| *FYB* | chr5:39126100-39126237 | **Yes** |  | - |
| *MAPK8IP3* | chr16:1794979-1795104 | (No) | Retained intron | 1.00 |
| *LIMA1* | chr12:50643466-50643569 | **Yes** | Weak candidate | -0.28 |
| *STXBP5* | chr6:147660315-147660374 | **Yes** |  | 0.03 |


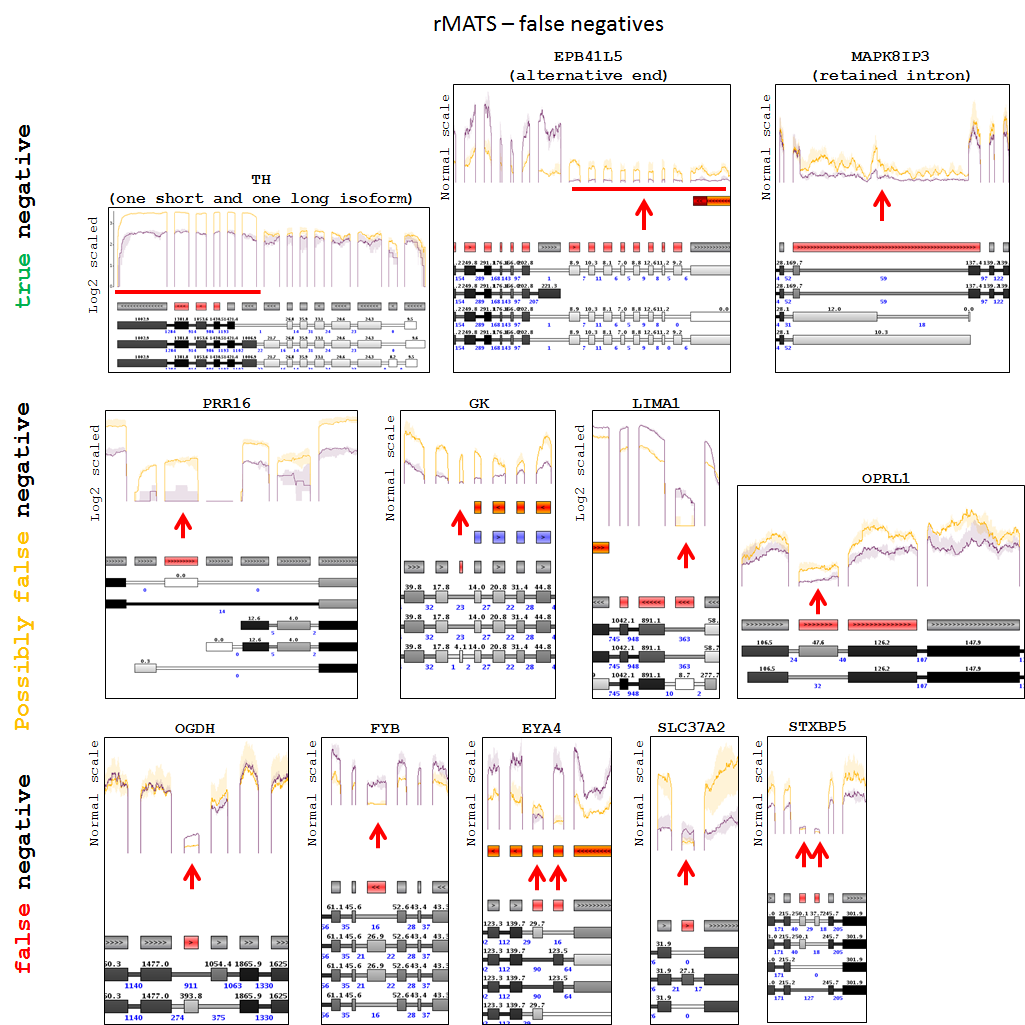


Supplementary Figure S17 – rMATS potential false negative results. Coverage profiles of 12 visually inspected genes with potential alternative splicing events only detected by all tools but using conservative results (Supplementary Table S6). *TH*, *EPB41L5* and *MAPK8IP3* are valid alternative splicing events, but they are not representing exon skipping events and therefore are true negative results. *PRR16* and *LIMA1* required looking at log-scaled coverage profiles to identify coverage changes. In these cases the isoform containing the alternatively spliced exon is much lower expressed than the isoform without the exon, and thus these results should be considered with care. *GK* shows also coverage changes in the exons surrounding the AS exon, which decreases our confidence in the result and *OPRL1* shows only a very small change. *OGDH*, *FYB*, *EYA4*, *SLC37A2* and *STXBP5* show very clear coverage changes and represent very likely false negative results.

Supplementary Table S12 – Cuffdiff potential false negative results. We visual inspected 30 (of 250) genes that are potential false negatives because they were detected by all tools but Cuffdiff. Genes were filtered by Manananggal evidence type (“combined” evidence) and sorted by Manananggal PSI p-value before selecting the top 30 genes. Most of the candidates are strong candidates for alternative splicing and only few of them were detected by Cuffdiff with low p-values (but not flagged as significant).

| **gene symbol** | **exon position** | **valid after**  **visual inspection** | **comment** | **Cuffdiff p‑value** |
| --- | --- | --- | --- | --- |
| ***SCRIB*** | chr8:144889722-144889784 | **Yes** |  | 0.66 |
| ***MYO1B*** | chr2:192265475-192265561 | **Yes** |  | 0.61 |
| ***RALGPS2*** | chr1:178861365-178861442 | **Yes** |  | **0.00** |
| ***DNM1*** | chr9:131010203-131010214 | **Yes** |  | 0.85 |
| ***PKM*** | chr15:72495363-72495529 | **Yes** |  | 1.00 |
| ***PCYT2*** | chr17:79865080-79865133 | **Yes** |  | **0.03** |
| ***MPDU1*** | chr17:7490217-7490335 | **Yes** |  | 0.43 |
| ***RABGAP1L*** | chr1:174846530-174846743 | **Yes** | Isoform switch | 0.62 |
| ***ARHGEF11*** | chr1:156908210-156908305 | **Yes** |  | 0.80 |
| ***SPIRE1*** | chr18:12459754-12459927 | **Yes** |  | 0.38 |
| ***ABI3BP*** | chr3:100537369-100537425 | **Yes** |  | **0.01** |
| ***SEC24C*** | chr10:75521856-75521924 | **Yes** |  | 0.42 |
| ***CLSTN1*** | chr1:9797556-9797612 | **Yes** |  | **0.01** |
| ***INPP5K*** | chr17:1419182-1419412 | **Yes** |  | 0.15 |
| ***HGSNAT*** | chr8:43051573-43051683 | **Yes** |  | 0.16 |
| ***POMZP3*** | chr7:76254839-76255000 | **?** | Antisense transcript | **0.04** |
| ***MACF1*** | chr1:39946592-39946702 | **Yes** |  | 0.44 |
| ***HSPD1*** | chr2:198363866-198364162 | **Yes** |  | 0.19 |
| ***PRC1*** | chr15:91512309-91512350 | **?** | Unlikely candidate | **0.02** |
| ***PNPLA8*** | chr7:108161920-108161965 | **Yes** |  | 0.21 |
| ***NDUFB2*** | chr7:140400670-140400759 | **Yes** | (requires log2 scale) | 0.13 |
| ***MGLL*** | chr3:127434696-127434773 | **Yes** |  | 0.10 |
| ***GPR56*** | chr16:57662549-57662714 | (No) | Alt. start | **0.02** |
| ***MICAL3*** | chr22:18309220-18309282 | **Yes** |  | 0.47 |
| ***PTK2*** | chr8:141958765-141958822 | **Yes** |  | **0.00** |
| ***RP11-43F13.1*** | chr5:1630524-1630681 | **Yes** |  | 0.50 |
| ***KLC1*** | chr14:104153418-104153548 | **Yes** |  | 0.37 |
| ***PUF60*** | chr8:144909199-144909337 | **?** | Low coverage exon | 0.12 |
| ***EVI5L*** | chr19:7921978-7922010 | **Yes** |  | 1.00 |
| ***NBEAL2*** | chr3:47033965-47034045 | **Yes** |  | 0.26 |


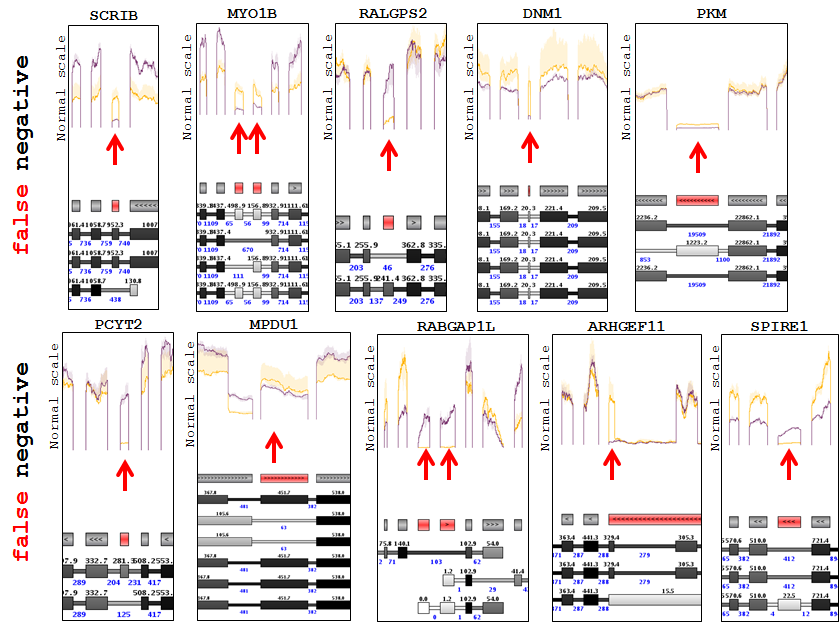


Supplementary Figure S18 – Cuffdiff potential false negative exon skipping events. Shown are exemplary coverage profiles of 10 visually inspected genes with potential alternative splicing events only detected by all tools but using conservative results (Supplementary Table S12). Most genes show strong signs of alternative splicing that were not picked up by Cuffdiff.

Supplementary Table S13 – Manananggal potential false negative results. We ordered the list of 105 genes that were identified as AS genes by rMATS, DEXSeq and Cuffdiff by coordinates first to get those events to the top that reported the exact same exon in rMATS and DEXSeq because they are more likely to be real events that were missed by Manananggal. We then sorted the results by the rMATS p-value and then DEXSeq p-value before we investigated the top 29 (all hits with rMATS p-value of 0). If an exon skipping event was reported via multiple exonic parts (e.g. the exon skipping events in DDR1 was split into 7 exonic parts) in DEXSeq, we put the best available p-value into the table. Most events would also be identified by Manananggal using a lower coverage ratio change threshold (below 15%).

| **gene** | **Position** | **p-val rMATS**  **(FDR)** | **DEXSeq p-value**  **(p-adj)** | **Manananggal**  **ratio** | **valid** | **comment** |
| --- | --- | --- | --- | --- | --- | --- |
| ***ANXA6*** | chr5:150490188-150490205 | 0 (0) | 1.17E-141 (3.69E-138) | **0.12** | Yes |  |
| ***CD99L2*** | chrX:149996779-149997769 | 0 (0) | 6.52E-94 (9.89E-91) | - | Yes | Unannotated exon, largely influence by an adjacent retained intron |
| ***MYOF*** | chr10:95152674-95152712 | 0 (0) | 9.35E-72 (9.22E-69) | 0.09 | Yes |  |
| ***YAP1*** | chr11:102080248-102080295 | 0 (0) | 5.56E-63 (4.58E-60) | -**0.13** | Yes |  |
| ***QKI*** | chr6:163899812-163899928 | 0 (0) | 4.46E-60 (3.42E-57) | -0.09 | Yes |  |
| ***PLOD2*** | chr3:145795649-145795711 | 0 (0) | 1.15E-50 (7.03E-48) | **0.13** | Yes |  |
| ***ECT2*** | chr3:172473273-172473365 | 0 (0) | 1.30E-41 (6.02E-39) | **0.148** | Yes |  |
| ***ZMIZ2*** | chr7:44799750-44799827 | 0 (0) | 2.30E-34 (8.40E-32) | **-0.10** | Yes |  |
| ***ACLY*** | chr17:40052873-40052902 | 0 (0) | 8.41E-32 (2.73E-29) | -0.07 | Yes |  |
| ***UAP1*** | chr1:162562525-162562572 | 0 (0) | 1.01E-30 (3.15E-28) | -0.08 | Yes |  |
| ***TUFT1*** | chr1:151534567-151534641 | 0 (0) | 4.70E-29 (1.36E-26) | **-0.13** | Yes |  |
| ***GOLGA2*** | chr9:131035064-131035144 | 0 (0) | 4.80E-26 (1.18E-23) | **0.148** | Yes |  |
| ***PPP3CB*** | chr10:75199630-75199659 | 0 (0) | 1.72E-23 (3.65E-21) | **-0.10** | Yes |  |
| ***DDR1*** | chr6:30852314-30852487 | 0 (0) | 1.10E-22 (2.23E-20) | **0.13** | Yes |  |
| ***BIN1*** | chr2:127815049-127815177 | 0 (0) | 1.34E-22 (2.71E-20) | **0.10** | Yes |  |
| ***PHPT1*** | chr9:139744958-139745012 | 0 (0) | 1.86E-17 (2.69E-15) | - | Yes | Largely influenced by an adjacent retained intron |
| ***CAP2*** | chr6:17507872-17507957 | 0 (0) | 3.83E-16 (5.00E-14) | 0.08 | Yes |  |
| ***LRP8*** | chr1:53738276-53738314 | 0 (0) | 5.44E-16 (7.01E-14) | **0.147** | Yes |  |
| ***TRERF1*** | chr6:42231058-42231306 | 0 (0) | 4.09E-15 (4.90E-13) | **-0.13** | Yes |  |
| ***TBC1D23*** | chr3:100030677-100030721 | 0 (0) | 1.48E-14 (1.68E-12) | **0.13** | Yes |  |
| ***INCENP*** | chr11:61908972-61908983 | 0 (0) | 5.86E-09 (3.60E-07) | -0.07 | Yes |  |
| ***C9orf89*** | chr9:95874161-95874220 | 0 (0) | 1.12E-07 (5.62E-06) | - | Yes | Manual selection of isoforms in the viewer allows identification with a coverage ratio change of -0.05 |
| ***DNM2*** | chr19:10919245-10919256 | 0 (0) | 5.14E-07 (2.30E-05) | 0.04 | Yes | There is a better exon skipping candidate in the same gene (coverage ratio change: 0.14) |
| ***TMPO*** | chr12:98938729-98938839 | 0 (0) | 1.79E-06 (7.26E-05) | -0.05 | (Yes) | Weak candidate |
| ***GRHL1*** | chr2:10102584-10102660 | 0 (0) | 4.92E-06 (1.82E-04) | -0.06 | (Yes) | Weak candidate |
| ***DROSHA*** | chr5:31531557-31531632 | 0 (0) | 7.90E-05 (2.15E-03) | -0.05 | Yes |  |
| ***ZDHHC16*** | chr10:99213556-99213603 | 0 (0) | 2.45E-04 (5.73E-03) | -0.06 | Yes |  |
| ***PLAA*** | chr9:26917095-26917163 | 0 (0) | 3.98E-03 (5.59E-02) | 0.05 | (Yes) | Weak candidate |
| ***CDS2*** | chr20:5166404-5166472 | 0 (0) | 4.16E-02 (2.86E-01) | 0.04 | (Yes) | Weak candidate |


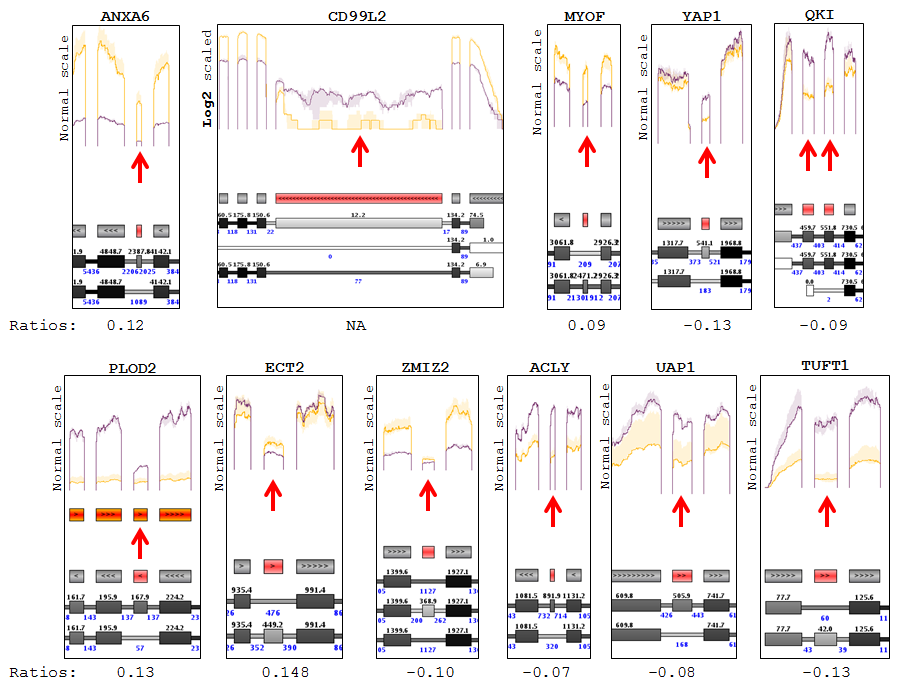


Supplementary Figure S19 – Manananggal potential false negative exon skipping events. Coverage profiles of 11 visually inspected genes with potential alternative splicing events detected by all tools but Manananggal using conservative results (Supplementary Table S13). Most, if not all of the investigated events show signs of alternative splicing. However, most of the visually inspected events are also detected by Manananggal, but were discarded because of our strict coverage ratio change threshold (15%). If applicable, coverage ratio changes detected by Manananggal were added below each exon skipping event.

Supplementary Table S14 – DEXSeq potential false negative exon skipping events. We visually inspected 20 (of 55, sorted by rMATS p-value) exon skipping events that were reported by Manananggal and rMATS with the same chromosomal positions. Most events show signs of alternative splicing. However, many events show only small changes and require a closer look to the splice junction read coverage. Please note that several events are overlapping antisense exons, but in most cases these do not seem to influence the coverage at the exon skipping event.

| **gene** | **exon** | **rMATS pvalue**  **(FDR)** | **valid** | **comment** |
| --- | --- | --- | --- | --- |
| *FMN2* | chr1:240341221-240341368 | 0 (0) | **Yes** | DEXSeq identifies on of the two alternative exons |
| *CTNND1* | multiple | 0 (0) | **Yes** |  |
| *AP1G2* | chr14:24035771-24035895 | 7.99E-15 (2.10E-12) | **Yes** | (Overlapping Antisense transcript) |
| *MTSS1L* | chr16:70699369-70699443 | 4.92E-14 (1.19E-11) | **Yes** | Unannotated junction |
| *CPNE1* | chr20:34243124-34243266 | 2.23E-13 (5.09E-11) | (**Yes**) | Overlapping sense transcript, *RBM1* |
| *DCAF15* | chr19:14063842-14064120 | 2.26E-12 (4.68E-10) | No | Overlapping antisense transcript |
| *B4GALT3* | chr1:161146702-161146896 | 2.30E-10 (3.87E-08) | (**Yes**) | Small change |
| *IGF2BP3* | chr7:23383337-23383472 | 9.29E-09 (1.28E-06) | (**Yes**) | Small change |
| *AK2* | chr1:33497145-33497262 | 3.24E-08 (4.06E-06) | **Yes** |  |
| *BTBD3* | chr20:11899734-11899824 | 3.85E-08 (4.77E-06) | **Yes** |  |
| *FAM49B* | chr8:130908918-130909100 | 1.21E-06 (1.17E-04) | (**Yes**) | small change |
| *RSRC2* | chr12:123007346-123007453 | 2.72E-06 (2.43E-04) | (**Yes**) | Small change |
| *ZC3H11A* | chr1:203765579-203765729 | 3.17E-06 (2.79E-04) | (**Yes**) | Small change |
| *PDLIM2* | chr8:22449064-22449181 | 5.48E-06 (4.56E-04) | (No) | Very small change |
| *TATDN2* | chr3:10320348-10320444 | 6.52E-06 (5.35E-04) | **Yes** |  |
| *MAZ* | chr16:29820861-29821085 | 2.04E-05 (1.45E-03) | (**Yes**) | Small change |
| *ACSF2* | chr17:48541887-48541960 | 2.11E-05 (1.49E-03) | (**Yes**) | Small change (antisense transcript) |
| *PPT2* | chr6:32121973-32122181 | 2.64E-05 (1.81E-03) | (**Yes**) | Rather an alternative start |
| *OSGEP* | chr14:20919416-20919611 | 7.99E-15 (2.10E-12) | (No) | Very small change |
| *AC024560.3* | chr3:197349058-197349201 | 4.92E-14 (1.19E-11) | **Yes** | Could be an alternative start exon |


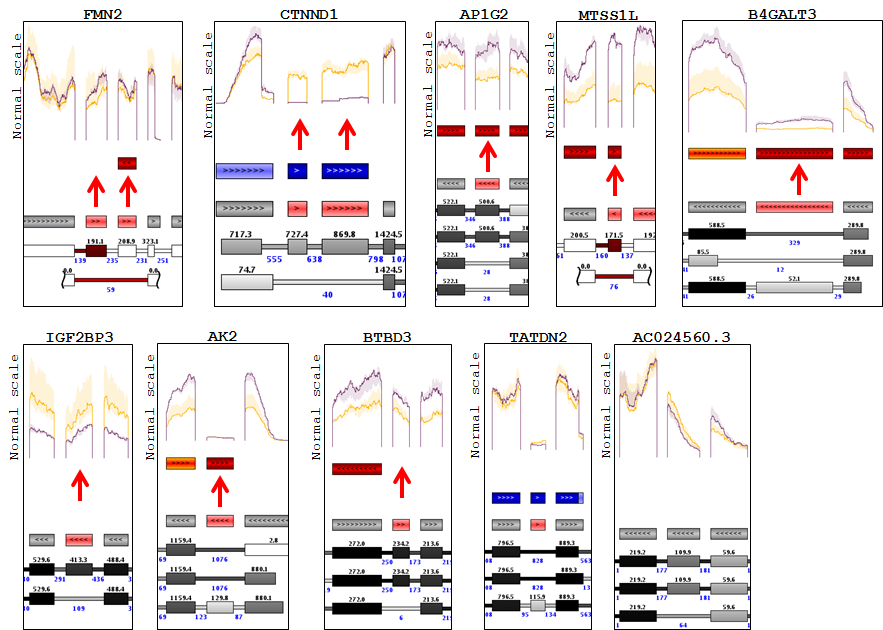


Supplementary Figure S20 – DEXSeq potential false negative exon skipping events. Shown are 10 examples of potential exon skipping events that were missed by DEXSeq. Please note that several of these events overlap exons in antisense directions, but this does not seem to interfere with the expression at the exon skipping event. While some examples show changes in the coverage (*FMN2, CTNND1, AP1G2, MTSS1L, TATDN2*) other examples require a closer look at the junction read counts that can be reviewed in the interactive web application.

**Chapter IV – Comparison of detected alternative splicing events including all results but retained introns**

4.1. Introduction

Comparing only the exon skipping events from rMATS and Manananggal to the result lists of DEXSeq and rMATS has the advantage that more information is available to detect these events. However, because the results from DEXSeq and Cuffdiff cannot be filtered for these events they easily generate potential false positive results (see Chapter III) for rMATS and Manananggal. Therefore we decided to also compare the whole result lists based on gene symbols including all types of alternative splicing events from rMATS (SE, A3SS, A5SS, MXE) and Manananggal (SE, ATE, EE), excluding retained introns.

Again, we compared two sets of results, one with lenient and one with strict criteria. For the lenient set we selected the Manananggal results from the conservative score list (5_5_0.7_0.15_true). We selected exon skipping events (SEs) and alternative terminal exon (ATE) events that had ‘combined’ support by junction reads and coverage ratio change. ATEs were selected that had at least one “unique_junction” (i.e. events that have a junction that is unique to a start or end exon) or were characterized as “double” class (events that identified two start or end exons with coverage ratio changes). Exon extension (EEs) events were filtered for events that use annotated junctions only. rMATS results included all skipped exon, alternative 3’ and 5’ acceptor sites and mixed event AS types. The lenient set also included all DEXSeq and Cuffdiff results with p-value < 0.05.

For the strict set we used a more strict coverage ratio threshold for Manananggal (15% from the 5_5_0.7_0.15_true result list) and additionally required that exon extensions used known (annotated) junctions. Further, we used only rMATS results with a FDR < 0.05, DEXSeq results with an adjusted p-value < 0.1 and Cuffdiff results that were flagged as significant in this data set.

4.2 Venn diagram

The Venn diagrams look very similar to the Venn diagrams focused on exon skipping events, with a low overall overlap (Figure 4 from the main article, also included below) and an additional large overlap between rMATS, DEXSeq and Manananggal. DEXSeq also shows a larger overlap to rMATS or Manananggal.

4.3 Potential false positives

To get lists of potential false positive results, we used the conservative result list of one tool and compared it to the lenient result lists of all other tools. Thereby, we obtained 81 (3.7% of conservative results) potential false positives for rMATS, 58 (12.8%) for Cuffdiff, 3531 (40.87%) for DEXSeq and 18 (1.3%) for Manananggal. The top 20 potential false positive results from each tool were visually inspected.

4.3.1 rMATS potential false positives

Twelve out of 20 potentially false positive results from rMATS showed signs of alternative splicing. Half of these twelve events showed only small changes, while the other half are probably true positive results. The remaining 8 results are likely real false positives and were mainly attributable to overlapping antisense transcripts and annotated fusion/read-through transcripts.

4.3.2 Cuffdiff potential false positives

Cuffdiff specified multiple gene symbols for some hits that thus represented outliers. If multiple identifiers were given, we visually inspected all of them and accepted them as true positives if at least one of the genes showed signs of alternative splicing. In principle this should artificially decrease the false positive rate of Cuffdiff. However, 16 out of 20 still represented false positive results, two are likely also false positives (*NBEAP1,RP11-403B2.7* and *BMP8B,OXCT2*), and the remaining two events (*CCDC15,SLC37A2* and *MTA1,TEX22*) were also detected by the other tools, referring to *SLC37A2* and *MTA1*.

4.3.3. DEXSeq potential false positives

Ten of the 20 genes inspected for DEXSeq were indeed false positives although many were among the best 300 DEXSeq hits. This can mainly be attributed to the expression of overlapping antisense transcripts. The remaining results are probably valid alternative splicing events, mostly referring to UTR extensions that cannot be detected by rMATS and Manananggal.

4.3.4 Manananggal potential false positives

Ten of 18 potential false positives reported by Manananggal are indeed false positives and a further two are likely false positives. Of the remaining six potential false positives, three appear to be true positives and three might be true positives with smaller effect.

4.3.5 Potential false positive results based on the overlap of only two tools

We also investigated how the results detected exclusively by two tools behaved. For this, we extracted the genes detected by DEXSeq and Manananggal, rMATs and Manananggal, rMATS and DEXSeq, Cuffdiff and rMATS and Cuffdiff and DEXSeq from the Venn diagram (Error: Reference source not found). The top 10 genes of each group were visually inspected.

4.4.1 rMATS & DEXSeq potential false positives

Most of the investigated events reported by rMATS and DEXSeq show signs of alternative splicing. However, most of them show only small effects.

4.4.2 rMATS & Manananggal potential false positives

Events reported by rMATS and Manananggal were more diffuse. About half of them might point towards alternative splicing events with small effects.

4.4.3 rMATS & Cuffdiff potential false positives

None of the five events reported by rMATS and Cuffdiff appear to be alternatively spliced.

4.4.4 DEXSeq & Manananggal potential false positives

The events detected by DEXSeq and Manananggal were all true positives and concerned almost exclusively alternative transcript start and end exons.

4.4.5 DEXSeq & Cuffdiff potential false positives

All events detected by DEXSeq and Cuffdiff appear to be valid alternative splicing events, including several retained introns (which we did not include for rMATS and Manananggal) and alternative transcript start and end exons.

4.4.6 Summary

The above results show that all tools suffer from overlapping antisense transcripts and annotated fusion/read-through transcripts, which leads to false positive results. One solution to this problem is to exclude fusion transcripts/read through transcripts before running the tools and to use strand specific RNA sequencing.

Considering that rMATs and Mananangal had only few potential false positive candidates to begin with, we believe that the false positive rate for these tools is rather low although most of the false positive candidates were indeed false positives. Only about 50% of the investigated false positive candidates of DEXSeq were actually false positives, the other half appears to be true positive results that were missed by all other tools. However, the other 50% are very likely real false positives even though they were among the best 300 results from DEXSeq. Considering the relatively large number of potential false positive results from DEXSeq (3531) we assume that the false positive rate is slightly higher. As has been shown for the exon skipping events before, Cuffdiff seems to produce a large number of false positive results in our setup.

4.5 Potential false negatives

To get a list of potential false negative results we used the lenient result list of one tool and compared it to the conservative result lists of all other tools. Thereby, we obtained 14 genes for rMATS, 368 for Cuffdiff, 27 for Manananggal and none for DEXSeq that were missed by these tools, but reported by all other tools (Venn diagrams not shown).

4.5.1 rMATS potential false negatives

All events that were detected by all tools but rMATS show signs of alternative splicing. Most of them represent alternative start or end exons that cannot be detected by rMATS.

4.5.2 Cuffdiff potential false negatives

Almost all events detected by all tools but Cuffdiff show signs of alternative splicing. Many of them were also very strong candidates.

4.5.3 DEXSeq potential false negatives

DEXSeq included all genes that were reported by all the other tools, thus we did not examine the false negative rate of DEXSeq.

4.5.4 Manananggal potential false negatives

Almost all genes that were reported by all tools but Manananggal showed signs of alternative splicing. Several of them showed only small changes, but some were also good candidates for alternative splicing.

4.5.5 Summary

All tools except DEXSeq, for which we could not identify genes that were reported by all the other tools, missed several alternative splicing events that were detected by the other tools. For example, rMATS cannot detect alternative terminal exons and thus its list of false negative results mainly included alternative splicing events of this type. Common for rMATS and Manananggal is that both missed several genes that showed only small signs of alternative splicing. Cuffdiff missed a larger number of very strong alternative splicing candidates, similar of what has been observed when we focused on exon skipping events.


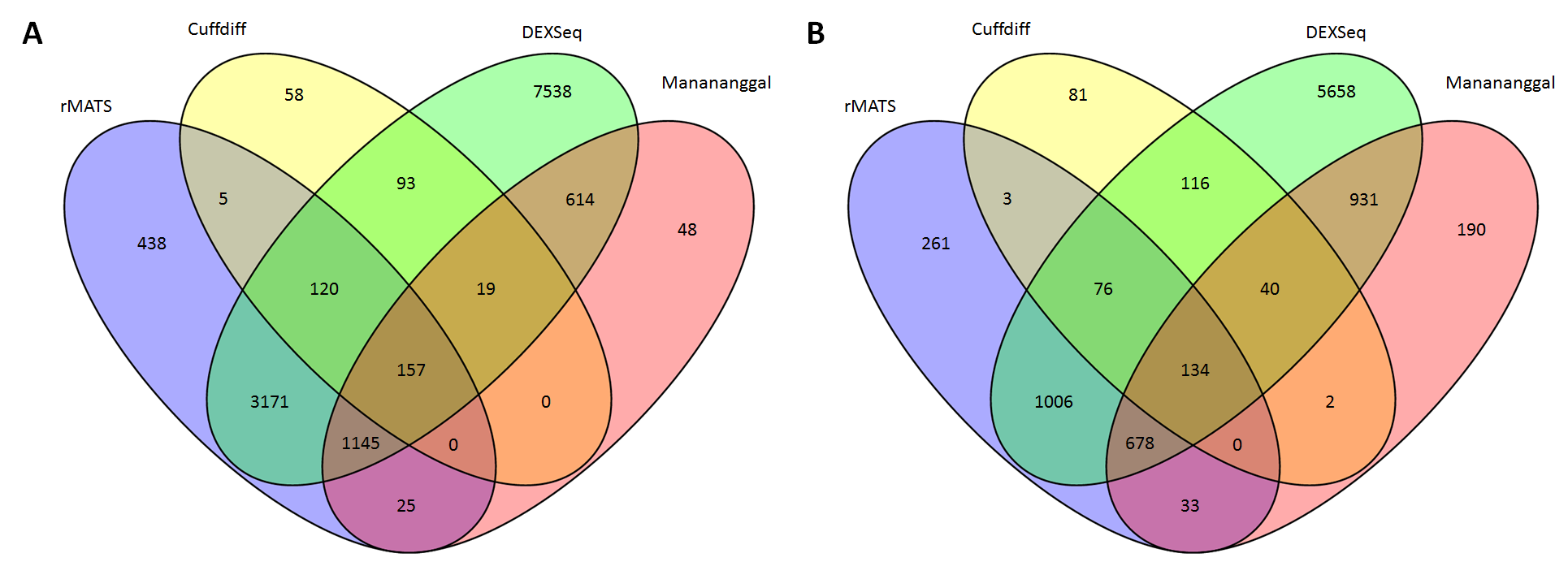


**Figure 4 - Comparison of genes predicted to be subject to alternative splicing using combined lists of exon skipping event, alternative terminal exons and exon extensions.** Genes reported by Manananggal were selected from the conservative score list (5_5_0.7_0.15_true). We selected exon skipping events and alternative terminal exon (ATE) events that had ‘combined’ support by junction reads and coverage ratio change. Further, ATEs required at least one “unique_junction” (i.e. events that have a junction that is unique to a start or end exon) or were characterized as “double” class (events that identified two start or end exons with coverage ratio changes). Exon extension events were filtered for events that use annotated junctions only. rMATS results included all skipped exon, alternative 3’ and 5’ acceptor sites and mixed event AS types. We selected events with a simple p-value < 0.05 (**A**) and events with a FDR < 0.05 (**B**). Similar, we used all DEXSeq results with p-value < 0.05 (**A**) and adjusted p-value < 0.1 (**B**). For Cufflinks we used all AS events flagged as “significant” in both cases.


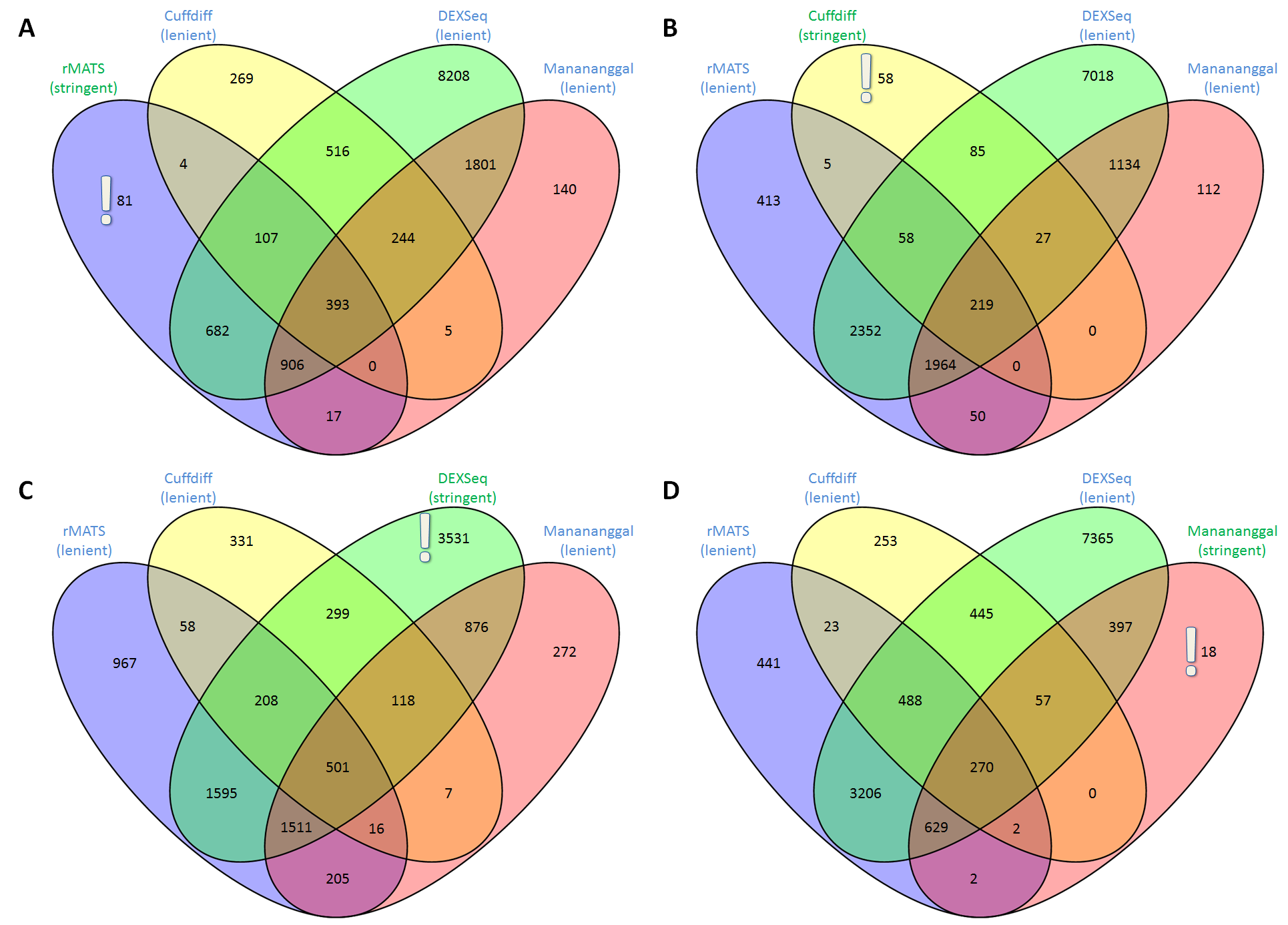


**Supplementary Figure S21 – Venn diagrams for identification of potential false positives.** Result lists of all tools were filtered by stringent and lenient criteria. We compared the stringent results of one tool to the lenient results of all other tools to keep the number of potential false positives as small as possible. Genes only reported by a single tool (marked by exclamation marks) were then selected as potential false positive candidates.

**Supplementary Table S15 – rMATS potential false positive results.** Genes with potential exon skipping events only reported by rMATS were sorted by p-value and the top 20 (of 81 candidates) were visually inspected. Only *PILRB* showed convincing evidence of alternative splicing. It actually has two equally good hits, one categorized as MXE and the other as A5SS. The skipped exon detected in *TEX22* is also a good candidate, but it actually belongs to the gene *MTA1* and both Cuffdiff (p-value 5.00E-05) and Manananggal (coverage ratio change 0.11) identify this exon in *MTA1* as well.

| **Gene** | **AS type** | **p-value (FDR)** | **Valid upon visual inspection** | **Comment** |
| --- | --- | --- | --- | --- |
| ***PILRB*** | A5SS | 0 (0) | **yes** | Uses a novel junction |
| ***RP1-309K20.6*** | MXE | 0 (0) | no | Readthrough/Fusion transcript |
| ***DYNLL2*** | SE | 0 (0) | no |  |
| ***ABHD14A-ACY1*** | MXE | 0 (0) | no | Readthrough/Fusion transcript |
| ***RP11-1021N1.1*** | MXE | 0 (0) | no | Readthrough/Fusion transcript |
| ***TEX22*** | SE | 0 (0) | (no) | Fusion transcript, event belongs to *MTA1* (identified by all tools). |
| ***APEX2*** | SE | 7.35E-13 (1.6E-10) | (**yes**) | Maybe a very small change |
| ***ZSCAN16-AS1*** | SE | 3.1E-11 (5.75E-09) | no | Overlapping antisense transcript |
| ***RP1-130H16.18*** | MXE | 6.45413E-11 (1.03E-08) | no | Readthrough/Fusion transcript |
| ***RP11-458D21.5*** | MXE | 7.83403E-10 (1.14E-07) | no | Readthrough/Fusion transcript |
| ***SEMA7A*** | SE | 1.83E-09 (2.74E-07) | (**yes**) | Maybe a very small change |
| ***AC006014.7*** | SE | 1.02E-08 (1.40E-06) | **yes** | Single isoform gene |
| ***ADCY3*** | SE | 1.24E-08 (1.68E-06) | (**yes**) | Maybe a very small change |
| ***TIMMDC1*** | SE | 1.86E-08 (2.44E-06) | (**yes**) | Maybe a very small change |
| ***TMX2-CTNND1*** | SE | 1.13E-07 (1.30E-05) | no | Readthrough/Fusion transcript |
| ***CADM4*** | SE | 3.37E-07 (3.60E-05) | **yes** | Small change |
| ***ZNF445*** | SE | 5.57E-07 (5.70E-05) | **yes** | Uses novel junction, small change |
| ***RNASEK*** | SE | 1.25E-06 (1.20E-04) | **yes** |  |
| ***IFFO2*** | SE | 1.38E-06 (1.32E-04) | (**yes**) | Maybe a very small change |
| ***DCAF10*** | SE | 1.74E-06 (1.60E-04) | (**yes**) | Maybe a very small change |

**Supplementary Table S16 – Cuffdiff potential false positives. Almost none of the visually inspected genes showed signs of alternative splicing. Several false positives included genes that do not seem to be expressed, genes that overlap other genes on the opposite strand and 1-2 exon genes. There are two exceptions, which are true positive results (*SLC37A2* and *MTA1*) that were included in the list of potential false positives because Cuffdiff reported them together with a different gene. Both genes seem to be alternatively spliced, but those were also reported by other tools.**

| **Gene*** | **AS type** | **p-value** | **Valid upon visual inspection** | **Comment** |
| --- | --- | --- | --- | --- |
| ***ZNF222,ZNF223*** | ? | 5.00E-05 | no |  |
| ***AC004448.5*** | ? | 5.00E-05 | no | Gene not expressed |
| ***MED28P3,RP11-410E4.1*** | ? | 5.00E-05 | no | Gene not expressed / single exon genes |
| ***TPPP*** | ? | 5.00E-05 | no | Overlapping antisense transcript |
| ***NBEAP1,RP11-403B2.7*** | ? | 5.00E-05 | (no) | Possibly some difference in *NBEAP1* |
| ***CCDC15,SLC37A2*** | Exon skipping | 5.00E-05 | **yes** | *SLC37A2* has an exon skipping event (also included in the conservative result lists of DEXSeq and Manananggal. rMATs identified the event with FDR = ~0.5) |
| ***MTA1,TEX22*** | Exon Skipping | 5.00E-05 | **yes** | MTA1 has an exon skipping event (identified by all tools) |
| ***HNRNPA3P3*** | ? | 5.00E-05 | No | Single exon gene |
| ***HLA-Z*** | ? | 5.00E-05 | no | Single exon gene |
| ***MUM1L1*** | ? | 5.00E-05 | no |  |
| ***CCDC141,TTN*** | ? | 5.00E-05 | no | Overlapping antisense transcripts |
| ***ASCL5*** | ? | 5.00E-05 | no |  |
| ***RP11-909N17.2*** | ? | 5.00E-05 | no |  |
| ***RHOV*** | ? | 5.00E-05 | no |  |
| ***IRF6,RP3-434O14.8*** | ? | 5.00E-05 | no |  |
| ***HIST2H2BF,***  ***RP11-196G18.21,***  ***RP11-196G18.3*** | ? | 5.00E-05 | no |  |
| ***LINC00327*** | ? | 5.00E-05 | no |  |
| ***BMP8B,OXCT2*** | ? | 5.00E-05 | (no) | Maybe there is some change in the 3’UTR |
| ***NUDT19*** | ? | 5.00E-05 | no |  |
| ***SWSAP1*** | ? | 5.00E-05 | no |  |

*If more than one gene is specified, we visually inspected all of them.

Affects especially very short (1-3 exon) transcripts

Supplementary Table S17 - DEXSeq false positives. We took the list of alternative splicing events only reported by DEXSeq (Error: Reference source not found) and sorted them by adjusted p-value before we manually investigated the top 20 events. About half of these events are very likely false positives, although they are among the top 300 results reported by DEXSeq. This is largely contributed to overlapping transcripts in antisense direction. Lacking information from junction reads it is much more difficult to exclude this type of event for DEXSeq. Those events that are likely true positives are mostly events that concern an extension of the 3’UTR. Because rMATS and Manananggal both use junction reads to identify exon extensions they cannot find this type of alternative splicing. However, Manananggal reports some of these events in categories that we excluded because they often lead to false positives (e.g. the alternative end exon in *FKBP1A* was classified as alt_end_shared_jun with combined support of split reads and coverage ratio change, the exon in *RPS23* and *C2orf68* were identified by ratio_only), but these will be difficult to find among the large number of false positives reported by ratio_only.

| ***Gene**** | **AS type** | **p-value (p.adj)** | **Valid upon visual inspection** | **Comment** |
| --- | --- | --- | --- | --- |
| ***CTD-2587H24.5*** |  | 1.05E-218 (6.31E-215) | no | Overlapping antisense transcript |
| ***POM121B*** |  | 2.63E-144 (8.79E-141) | no | Weird coverage pattern, probably a region with mapping problems |
| ***RPS23*** | Exon extension | 9.31E-143 (3.01E-139) | **yes** | 3‘ UTR extension |
| ***EML5*** |  | 2.23E-132 (6.52E-129) | no | Overlapping antisense transcript |
| ***MRPS30*** | Exon extension | 1.45E-78 (1.65E-75) | **yes** | 3’ UTR extension |
| ***RPL31*** |  | 1.44E-74 (1.51E-71) | no | Overlapping antisense transcript |
| ***S100A16*** | Exon extension | 1.33E-70 (1.28E-67) | (**yes**) | 3’ UTR extension |
| ***SLC51A*** |  | 1.22E-69 (1.17E-66) | no | Overlapping antisense transcript |
| ***STMN3*** | Multiple | 6.45E-63 (5.31E-60) | **yes** | Unannotated short isoform |
| ***LINC00973*** |  | 1.09E-62 (8.85E-60) | no |  |
| ***FAM110A*** | Alt. start | 1.55E-60 (1.20E-57) | **yes** | Alternative start exon |
| ***SRSF9*** |  | 9.42E-60 (7.18E-57) | no | Overlapping antisense transcript appears as retained intron |
| ***C4orf3*** | Exon extension | 3.99E-59 (2.99E-56) | **yes** | 3’ UTR extension |
| ***TP53BP1*** |  | 1.72E-57 (1.24E-54) | no | Overlapping antisense transcript |
| ***NRG4*** |  | 3.64E-57 (2.59E-54) | no | Only expressed in one cell line |
| ***ETNK1*** | Exon extension | 6.86E-57 (4.84E-54) | (**yes**) | Possible changes in the 3’ UTR |
| ***SMIM12*** | Exon extension | 3.56E-56 (2.47E-53) | **yes** | 3’ UTR extension |
| ***AC137932.6*** |  | 1.81E-55 (1.23E-52) | no |  |
| ***TP53TG5*** |  | 2.97E-55 (2.01E-52) | no | Overlapping antisense transcript |
| ***SNX1*** |  | 4.57E-55 (3.07E-52) | no |  |

**Supplementary Table S18 - Manananggal potential false positives.** Most genes that were detected by all tools but Manananggal showed only very small changes. Some exceptions were *TAP2*, *GEMIN8* and *CYP51A1* which might be real alternative splicing events. Further, most of the false positives were alternative start or end exon events where only one exon had a sufficient change in the coverage ratio, therefore they have a higher probability of being false positives.

| **gene** | **type** | **p-value (PSI)** | **ratio change** | **Valid upon visual inspection** | **Comment** |
| --- | --- | --- | --- | --- | --- |
| ***NPIPB5*** | alt_end_unique_jun | 7.76E-03 | 0.15 | no |  |
| ***AC069368.3*** | alt_end_shared_jun_double | 4.89E-02 | -0.42 | no | Overlapping sense transcript |
| ***TAP2*** | alt_start_unique_jun | 1.61E-04 | 0.23 | **yes** | Alternative start exon |
| ***STAMBPL1*** | alt_start_unique_jun | 3.41E-02 | -0.21 | no |  |
| ***CD68*** | alt_start_unique_jun | 2.73E-02 | -0.19 | no | Incomplete UTR coverage |
| ***TIRAP*** | alt_5_prime_exon_end | 6.48E-03 | NA | (**yes**) | Maybe a very small change |
| ***ZNF780A*** | alt_start_unique_jun | 2.25E-02 | -0.23 | (**yes**) | Maybe a very small change |
| ***GEMIN8*** | alt_start_unique_jun | 3.13E-02 | 0.19 | **yes** | Small change |
| ***RP1-309F20.3*** | exn_skipping | 1.15E-02 | -0.17 | no |  |
| ***CYP51A1*** | alt_start_shared_jun_double | 1.52E-02 | -0.19 | **yes** |  |
| ***SOX7*** | alt_start_unique_jun | 8.41E-04 | 0.27 | no | Readthrough/Fusion transcript |
| ***MMP19*** | alt_end_unique_jun | 4.05E-02 | 0.15 | no | Overlapping antisense transcript |
| ***RP11-73M18.2*** | exn_skipping | 5.70E-03 | -0.2 | no | Readthrough/Fusion transcript |
| ***CREB5*** | alt_start_unique_jun | 2.51E-03 | -0.57 | (no) | Maybe some weak change |
| ***SHCBP1*** | alt_start_unique_jun | 2.09E-02 | -0.21 | (**yes**) | Maybe a very small change |
| ***BCL2L2-PABPN1*** | alt_end_unique_jun | 3.35E-02 | 0.23 | no | Readthrough/Fusion transcript |
| ***TTI2*** | alt_start_unique_jun | 4.64E-02 | -0.24 | (no) | Maybe some weak change |
| ***AP000347.2*** | exn_skipping | 1.59E-02 | -0.16 | no |  |


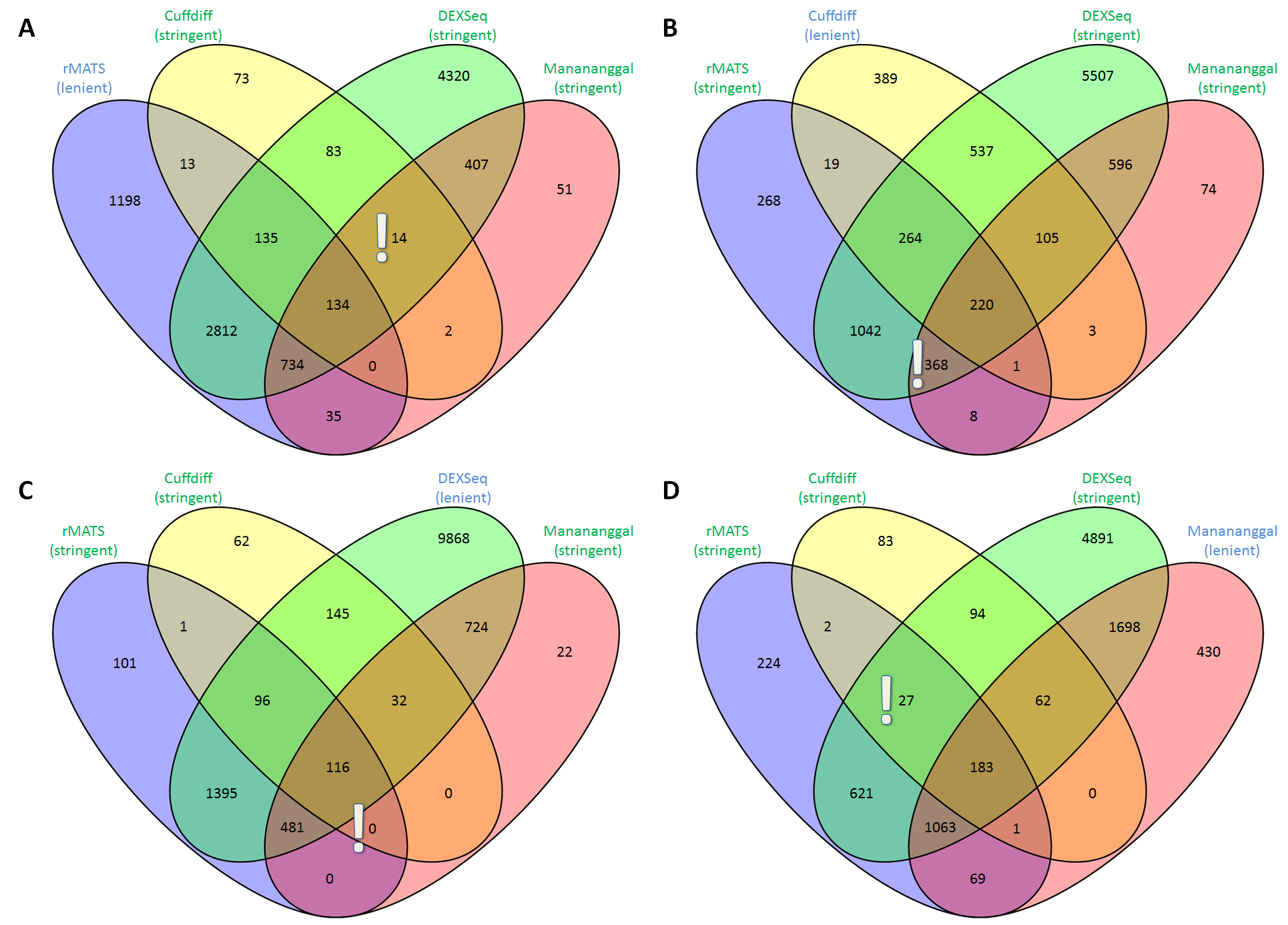


**Supplementary Figure S22 – Venn diagrams for identification of potential false negatives.** Result lists of all tools were filtered by stringent and lenient criteria. We compared the lenient results of one tool to the stringent results of all other tools. Genes that were reported by a three out of four tools (marked by exclamation marks) were then selected as potential false negative candidates.

**Supplementary Table S19 – rMATS potential false negatives. All events that were detected by all tools but rMATS show signs of alternative splicing. Most of them represent alternative start or end exons that cannot be detected by rMATS.**

| **Gene** | **Valid upon visual inspection** | **Comment** |
| --- | --- | --- |
| ***CDK18**** | yes | One short and one long isoform |
| ***CCDC85C**** | yes | Alternative start exon |
| ***TH*** | yes | One short and one long isoform |
| ***PRR16**** | yes | Exon skipping |
| ***GNAS**** | yes | Alternative start exon |
| ***HSPA14**** | yes | Alternative end exon |
| ***GNAI2*** | (yes) | Possibly an alternative start exon and/or 5’ UTR extension (weaker candidate) |
| ***MARVELD2**** | yes | Alternative start exon |
| ***JPH2**** | yes | One short and one long isoform |
| ***ZNF846*** | yes | Alternative start and/or end exons |
| ***FYB**** | yes | Exon skipping |
| ***RHBDL1*** | yes | Alternative start exon (lowly expressed genes) |
| ***LIMA1**** | yes | Alternative start exon or UTR extension |
| ***TRABD**** | yes | Alternative start exon |

* marked events are strong candidates

**Supplementary Table S20 - Cuffdiff false negatives.** Almost all events detected by all tools but Cuffdiff show signs of alternative splicing. Many of them were also very strong candidates.

| **Gene** | **PSI**  **p-value** | **Ratio**  **change** | **Valid upon visual inspection** | **Comment** |
| --- | --- | --- | --- | --- |
| ***SCRIB**** | 3.77E-07 | 0.47 | **yes** | Exon skipping |
| ***MYO1B**** | 4.72E-07 | 0.46 | **yes** | Exon skipping |
| ***DNM1**** | 9.73E-07 | 0.32 | **yes** | Exon skipping |
| ***PKM**** | 1.1E-06 | 0.23 | **yes** | Exon skipping |
| ***MPDU1**** | 2.38E-06 | 0.16 | **yes** | Exon skipping (+exon extension) |
| ***RABGAP1L**** | 2.63E-06 | -0.54 | **yes** | Multiple (exon skipping + alt. start) |
| ***ARHGEF11**** | 3.73E-06 | 0.46 | **yes** | Exon skipping |
| ***SPIRE1**** | 4.7E-06 | -0.55 | **yes** | Exon skipping |
| ***SEC24C**** | 8.02E-06 | 0.21 | **yes** | Exon skipping |
| ***ZDHHC3*** | 8.09E-06 | -0.24 | **yes** | Alt. end exon |
| ***EPN3*** | 1.04E-05 | 0.23 | (no) | Very weak alt. start exon |
| ***HGSNAT**** | 1.33E-05 | -0.32 | **yes** | Exon skipping |
| ***MACF1**** | 2E-05 | -0.32 | **yes** | Exon skipping |
| ***HSPD1*** | 2.05E-05 | -0.16 | (**yes**) | Weak exon skipping |
| ***RP11-35G9.3*** | 2.6E-05 | -0.29 | no | Overlapping transcripts in both direction |
| ***C1orf109**** | 2.7E-05 | NA | **yes** | alt_5_prime_exon_end |
| ***NDUFB2*** | 2.78E-05 | -0.37 | **yes** | Exon skipping |
| ***MGLL*** | 3.42E-05 | -0.68 | **yes** | Exon skipping |
| ***PFDN5*** | 4.07E-05 | 0.18 | (**yes**) | Weak alt. start/exon skipping |
| ***MICAL3**** | 4.67E-05 | 0.31 | **yes** | Exon skipping |

* marked events are strong candidates

**Supplementary Table S21 – Manananggal false negatives.** Almost all genes that were reported by all tools but Manananggal showed signs of alternative splicing. Several of them showed only small changes, but some were also good candidates for alternative splicing.

| ***gene*** | **rMATS**  **p-value** | **Valid upon visual inspection** | **Comment** |
| --- | --- | --- | --- |
| ***TMPO*** | 0.00E+00 | (**yes**) | Weak skipped exon |
| ***PSAP*** | 0.00E+00 | (**yes**) | Weak skipped exon |
| ***PHPT1**** | 0.00E+00 | **yes** | Exon skipping |
| ***PLAA*** | 0.00E+00 | **yes** | Exon skipping |
| ***C9orf89**** | 0.00E+00 | **yes** | Exon skipping |
| ***RPAP3**** | 1.98E-12 | **yes** | alt. start exon (and weak skipped exon) |
| ***SNPH**** | 2.80E-12 | **yes** | Exon extension |
| ***PARP2**** | 1.13E-11 | **yes** | Exon extension |
| ***RREB1*** | 1.45E-11 | **yes** | Retained intron |
| ***DENND4C*** | 1.24E-08 | **(yes)** | Exon skipping, junction support lacking |
| ***ALS2CL*** | 3.18E-08 | **yes** | Exon skipping |
| ***FHL1**** | 5.08E-07 | **yes** | Alternative start exons |
| ***QSOX1**** | 2.89E-06 | **yes** | 3’ UTR extension and (very weak) exon skipping |
| ***MIR29B1*** | 6.42E-06 | **yes** | Multiple changes, one alternative end exon is not annotated |
| ***ASIC1**** | 9.55E-06 | **yes** | Exon skipping |
| ***DDX46*** | 1.50E-05 | **yes** | Exon skipping |
| ***DNAJC16*** | 2.79E-05 | **yes** | Exon skipping/Exon extension or alt. start |
| ***SLC25A37*** | 8.43E-05 | (no) | Maybe a weak exon extension |
| ***PTPRH*** | 2.18E-04 | (**yes**) | Possibly an alternative transcript end exon that |
| ***RNF216*** | 2.22E-04 | **yes** | Exon extension |

* marked events are strong candidates

**Supplementary Table S22 - Events reported only by DEXSeq and Manananggal.** All events that we visually inspected and that were detected by DEXSeq and Manananggal showed signs of alternative splicing. Most of them were alternative start exons that cannot be detected by rMATS.

| **gene** | **Type**  **(Manananggal)** | **p-value (PSI)**  **Manananggal** | **p-val**  **DEXSeq** | **valid** | **Comment** |
| --- | --- | --- | --- | --- | --- |
| ***SMAGP*** | alt_start_unique_jun_double | 3.4744E-4 | 7.49645E-96 | yes |  |
| ***UBB*** | alt_3_prime_exon_end  (also an alternative start) | 2.7083E-2 | 5.54126E-43 | yes | Alternative start exon with alternative splice donor. Manananggal |
| ***DDX10*** | alt_start_shared_jun_double | 1.3583E-2 | 2.42931E-50 | yes |  |
| ***RP11-166D19.1*** | alt_5_prime_exon_end  (actually an alternative start) | 5.9398E-3 | 2.46812E-92 | yes | The event reported by Manananggal points to the correct exon, but the event is actually an alternative start exon (this event type is lost after automatic isoform removal) |
| ***FURIN*** | alt_start_shared_jun_double | 5.2769E-4 | 3.7619E-37 | yes |  |
| ***SET*** | alt_start_unique_jun_double | 2.4384E-2 | 1.7715E-180 | yes |  |
| ***CCDC120*** | alt_start_unique_jun_double | 3.5678E-5 | 4.2939E-39 | yes |  |
| ***ANP32E*** | alt_start_unique_jun_double | 2.9902E-5 | 2.27035E-19 | yes |  |
| ***MYADM*** | exon extension | 6.7074E-5 | 3.56222E-38 | yes |  |
| ***VGLL4*** | alt_start_unique_jun_double | 4.5991E-4 | 4.95475E-54 | yes |  |

Supplementary Table S23 - Events reported only by rMATS and Manananggal. Almost all of the reported events showed only very small changes and thus have a higher risk of being false positives. Most of them were referring to alternative 3’ or 5’ exon extensions. These extensions are only identified by split reads by both tools, lacking further evidence from changes in the exon coverage for example.

| **gene** | **Type**  **(Manananggal)** | **p-value (PSI)**  **Manananggal** | **p-val**  **rMATS** | **valid** | **Comment** |
| --- | --- | --- | --- | --- | --- |
| ***RP11-438J1.1*** | exn_skipping  &  (exon extension) | 3.1132E-3 | 1.02241E-05 | yes/? | rMATS reports this as A3SS event affecting a different exon. Manananggal reports an exon skipping event (that seems valid) and the same A3SS rMATS reported (showing a small change). |
| ***GSTCD*** | alt_3_prime_exon_end | 1.3861E-2 | 2.75718E-05 | ? | Small change (rMats: 9%, Manananggal: 7.7%) |
| ***ZFPL1*** | alt_3_prime_exon_end | 1.0246E-2 | 3.26E-04 | ? | Very small change (rMATS: , Manananggal: 1.7%) |
| ***AIF1L*** | alt_5_prime_exon_end | 3.0265E-3 | 2.66E-02* | yes/no | The extension concerning exon 2 reported by Manananggal seems valid. rMATS reported the 2^nd^ to last exon that does not seem to be different. |
| ***TCN1*** | alt_5_prime_exon_end | 4.9876E-4 | 8.34E-03* | no | Manananggal reported an exon extension and rMATS reported an exon skipping event. Both events seem rather unlikely. |
| ***PHF5A*** | alt_3_prime_exon_end | 2.4321E-2 | 7.10E-04 | ? | Very small change (rMATS: 1.1%, Manananggal: 2%) |
| ***TOE1*** | alt_5_prime_exon_end &  (alt_start_unique_jun) | 3.5841E-2 | 9.10E-04* | yes |  |
| ***RP11-345J4.5*** | alt_5_prime_exon_end | 4.0716E-2 | 6.57E-03* | no | Overlapping sense and antisense transcripts. Change would is small. rMATS points to the same exon as Manananggal, but predicted an exon skipping event. |
| ***MRE11A*** | alt_start_unique_jun | 1.2156E-3 | 3.09E-02* | yes/no | The alternative start exon seems valid, but the A3SS event reported by rMATS does not. |
| ***PANX2*** | exn_skipping | 2.7231E-3 | 6.34E-02* | yes |  |

*FDR > 0.05

Supplementary Table S24 - Events reported only by rMATS and DEXSeq. Most events show signs of alternative splicing, although several appear only as small changes.

| **gene** | **Type**  **(rMATS)** | **p-value**  **rMATS** | **p-value**  **DEXSeq** | **valid** | **Comment** |
| --- | --- | --- | --- | --- | --- |
| ***PXN-AS1*** | SE | 0 | 2.11E-04 | (no) | Overlapping antisense transcript. There might still be an exon skipping event with small change. |
| ***EHBP1L1*** | SE | 0 | 2.48384E-07 | ? | Complex case, might be caused by single exon transcript. Exon exclusion junction is novel (chr11:65349436 - 65351712) |
| ***FAM122B*** | SE | 0 | 2.42773E-19 | no/yes | rMATS reports this as exon skipping event, which actually points to an alternative end exon. |
| ***EPN1*** | SE | 0 | 7.2599E-245 | yes | Refers to two unannotated exons. Might include an alternative start exon. |
| ***C17orf62*** | SE | 0 | 5.28044E-10 | yes | Small change. |
| ***CA5B*** | SE | 0 | 2.1844E-10 | yes | Has an alt. Start and/or UTR extension. The exon skipping event could be an artifact of stacked junction reads. |
| ***SMARCC2*** | SE | 0 | 2.26963E-23 | yes |  |
| ***MPZL1*** | SE | 0 | 9.83257E-10 | yes | Small change. |
| ***MCAM*** | SE | 0 | 7.80465E-08 | yes | Small change. |
| ***TPRA1*** | SE | 0 | 1.69496E-07 | yes | Junction skips 3 exons. DEXSeq reports an 3’-UTR extension that might also be valid. |

**Supplementary Table S25 - Events reported by Cuffdiff and rMATS.** None of the events reported only by Cuffdiff and rMATS was a confident alternative splicing event. One candidate (*PID1*) could be alternatively spliced, but the gene shows a high degree of differential expression, which makes it hard to tell whether this is a true positive.

| **gene** | **Type**  **(rMATS)** | **p-value**  **rMATS** | **p-value**  **Cuffdiff** | **valid** | **Comment** |
| --- | --- | --- | --- | --- | --- |
| ***TFF3*** | SE | 1.38E-02* | 5.00E-05 | no |  |
| ***RP1-92O14.3*** | SE | 2.26E-02* | 6.50E-04 | no |  |
| ***PDZD8*** | SE | 1.37E-02* | 4.50E-04 | no |  |
| ***RASAL2*** | A3SS | 5.62E-04* | 5.00E-05 | no |  |
| ***PID1*** | SE | 2.31E-02* | 5.00E-05 | ? | Probably false positive, genes have strong differential expression. |

**Supplementary Table S26 - Events reports by Cuffdiff and DEXSeq**. All of the visually inspected events showed signs of alternative splicing.

| **gene** | **p-value**  **Cuffdiff** | **p-value**  **DEXSeq** | **valid** | **Comment** |
| --- | --- | --- | --- | --- |
| ***DNAJB2*** | 5.00E-05 | 7.1807E-117 | yes | Retained intron |
| ***PTPRN2*** | 5.00E-05 | 6.28271E-93 | yes | Novel, shorter isoform |
| ***EHD4*** | 5.00E-05 | 9.48467E-66 | yes | Exon skipping or alternative transcript end |
| ***ALPK3*** | 5.00E-05 | 3.34444E-56 | yes | Novel, shorter isoform |
| ***EPB41L4B*** | 5.00E-05 | 7.05279E-39 | yes | Alternative Transcript end |
| ***CIAO1*** | 1.00E-03 | 5.18634E-37 | (yes) | Retained intron |
| ***COMMD7*** | 5.00E-05 | 1.06603E-34 | yes | Retained intron |
| ***TRAK1*** | 1.05E-03 | 6.68482E-34 | yes | Alternative transcript end |
| ***AKR1C1*** | 5.00E-05 | 2.41128E-30 | yes | Alternative transcript start |
| ***TEKT4P2*** | 5.00E-05 | 4.81614E-30 | yes | Alternative transcript end |

1 Anders, S. & Huber, W. Differential expression analysis for sequence count data. *Genome biology* **11**, R106, doi:10.1093/bioinformatics/bts452

10.1186/gb-2010-11-10-r106 (2010).

2 Lin, L. *et al.* Alternative isoform regulation in human tissue transcriptomes. *Proceedings of the National Academy of Sciences of the United States of America* **456**, 470-476, doi:10.1073/pnas.1419161111

10.1038/nature07509 (2008).
