# Supplementary figures and images for: Manananggal - a novel viewer for alternative splicing events
Source: BMC Bioinformatics. 2017 Feb 21;18:120. doi: 10.1186/s12859-017-1548-5 (PMC5319012; doi:10.1186/s12859-017-1548-5)

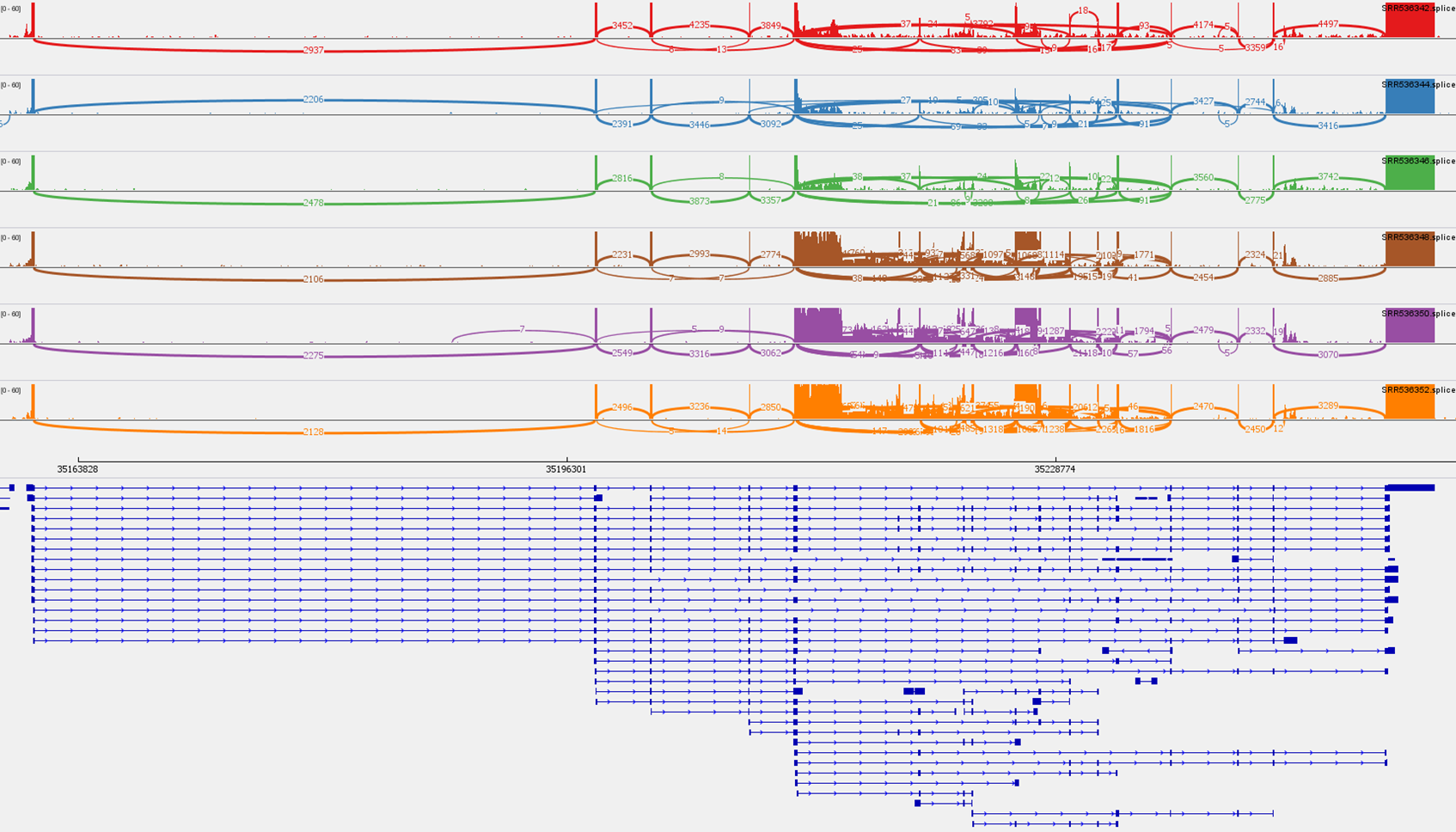

Supplement: Additional file 2: Figure S1. — Additional figure showing the example of CD44 alternative splicing visualized in IGV. (PNG 430 kb) [file 12859_2017_1548_MOESM2_ESM.png]

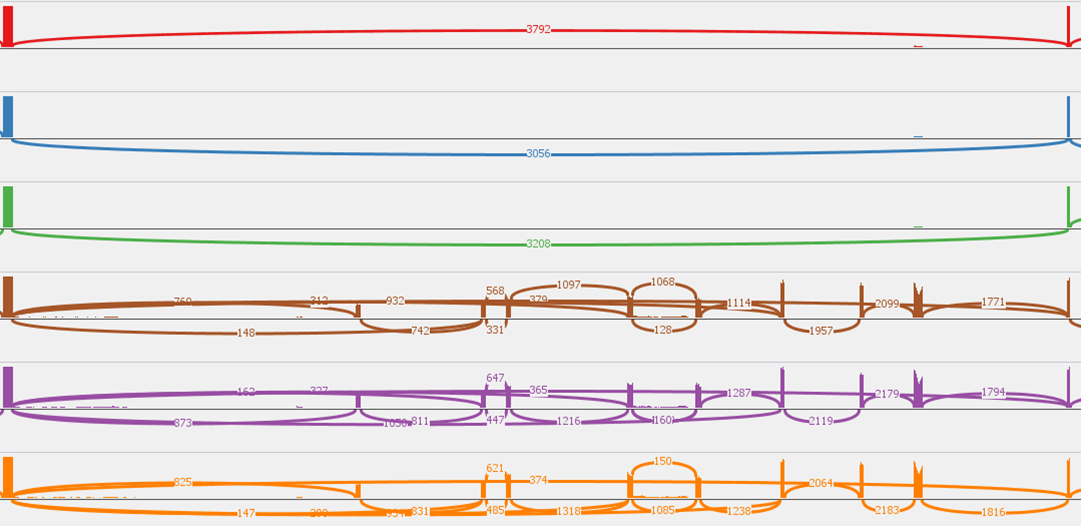

Supplement: Additional file 3: Figure S2. — Additional figure showing the example of CD44 alternative splicing visualized in IGV (zoom in). (PNG 125 kb) [file 12859_2017_1548_MOESM3_ESM.png]

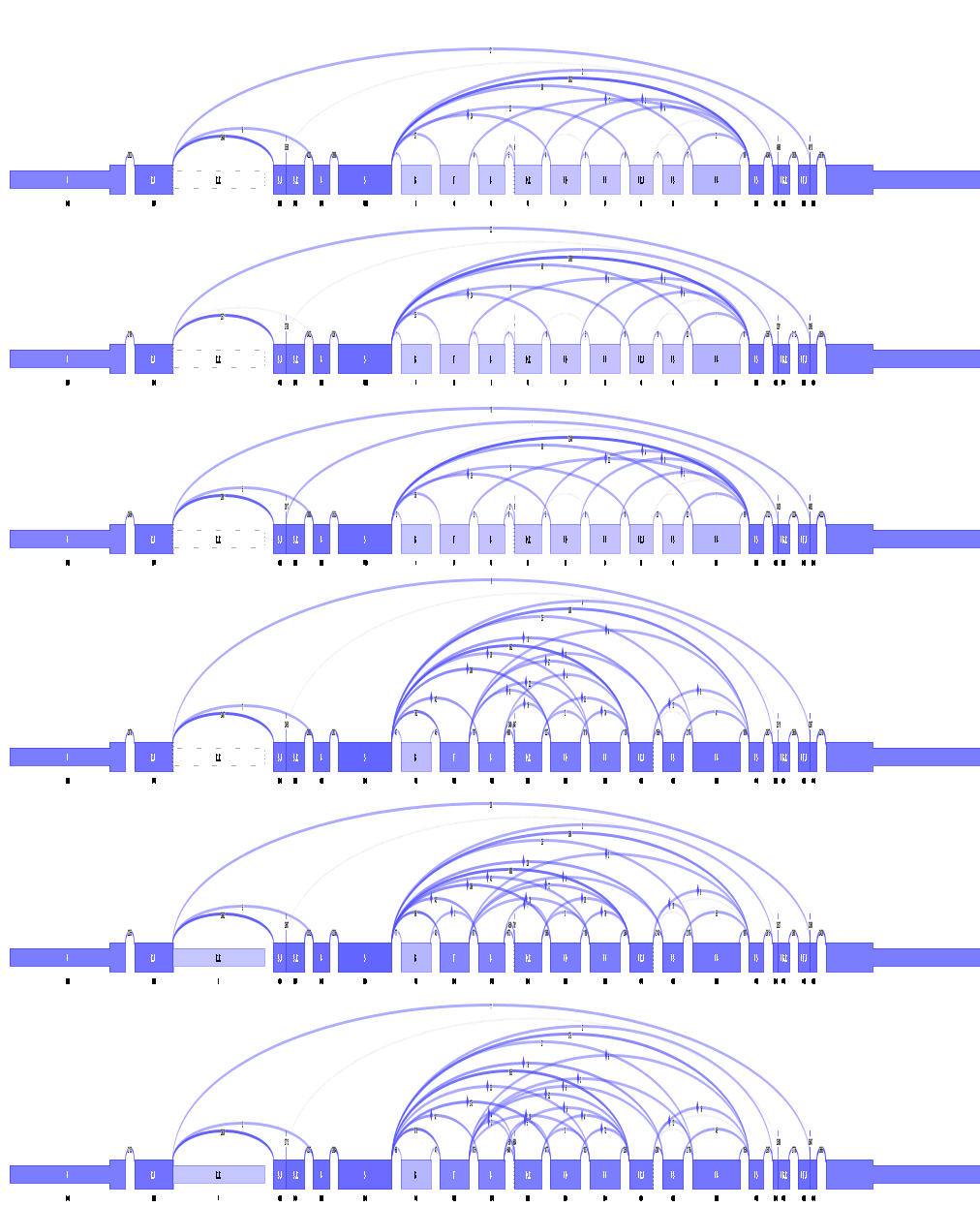

Supplement: Additional file 4: Figure S3. — Additional figure showing the example of CD44 alternative splicing visualized using SpliceSeq. (PNG 212 kb) [file 12859_2017_1548_MOESM4_ESM.png]

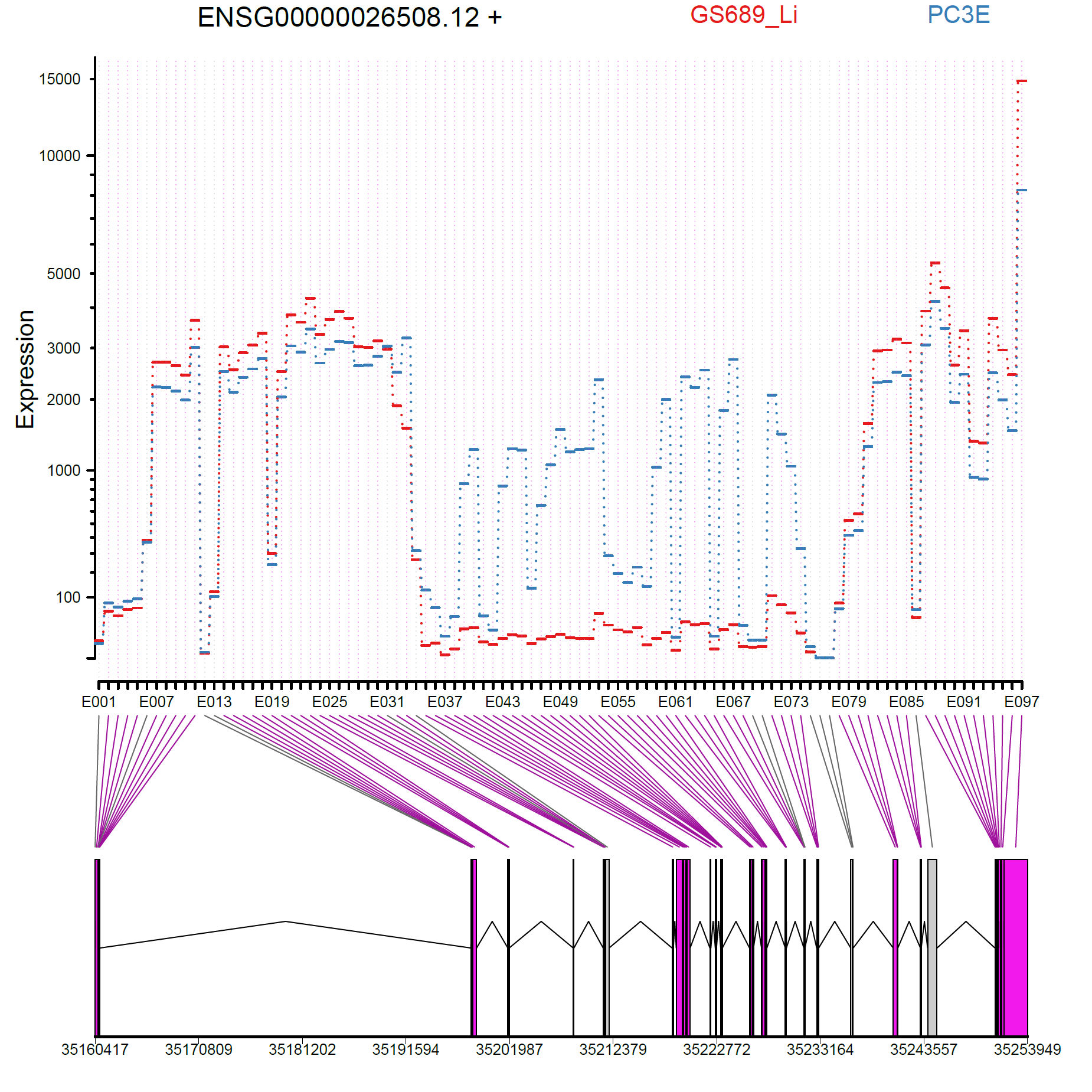

Supplement: Additional file 5: Figure S4. — Additional figure showing the example of CD44 alternative splicing visualized in DEXSeq. (PNG 303 kb) [file 12859_2017_1548_MOESM5_ESM.png]
